# Species conservation profiles of spiders (Araneae) endemic to mainland Portugal

**DOI:** 10.3897/BDJ.7.e39315

**Published:** 2019-10-08

**Authors:** Vasco Veiga Branco, Sergio Henriques, Carla Rego, Pedro Cardoso

**Affiliations:** 1 Laboratory for Integrative Biodiversity Research (LIBRe), Finnish Museum of Natural History, University of Helsinki, Helsinki, Finland Laboratory for Integrative Biodiversity Research (LIBRe), Finnish Museum of Natural History, University of Helsinki Helsinki Finland; 2 FCUL - Faculty of Sciences of the University of Lisbon, Lisbon, Portugal FCUL - Faculty of Sciences of the University of Lisbon Lisbon Portugal; 3 Institute of Zoology, Zoological Society of London, Regent's Park, London NW1 4RY, London, United Kingdom Institute of Zoology, Zoological Society of London, Regent's Park, London NW1 4RY London United Kingdom; 4 Centre for Biodiversity & Environment Research, Department of Genetics, Evolution and Environment, University College London, Gower Street, London, WC1E 6BT, London, United Kingdom Centre for Biodiversity & Environment Research, Department of Genetics, Evolution and Environment, University College London, Gower Street, London, WC1E 6BT London United Kingdom; 5 IUCN SSC Spider & Scorpion Specialist Group, Helsinki, Finland IUCN SSC Spider & Scorpion Specialist Group Helsinki Finland; 6 Centro de Ecologia, Evolução e Alterações Ambientais (cE3c), Faculdade de Ciências da Universidade de Lisboa, Campo Grande, Lisboa, Portugal Centro de Ecologia, Evolução e Alterações Ambientais (cE3c), Faculdade de Ciências da Universidade de Lisboa, Campo Grande Lisboa Portugal

**Keywords:** Arachnida, Arthropoda, Species distribution modelling, extinction risk, IUCN, Red List, Iberian Peninsula

## Abstract

**Background:**

The Iberian Peninsula is a diverse region that contains several different bioclimatic areas within one confined space, leading to high biodiversity. Portugal distinguishes itself in this regard by having a high count of spider species (829) and a remarkable number of endemic spider species (42) for its size (approximately 88,890 km2). However, only one non-endemic species (*Macrothele calpeiana*) is currently protected by the Natura 2000 network and no endemic spider species (aside from *Anapistula ataecina*) has been assessed according to the IUCN Red List criteria. The objective of this paper is to assess all non-assessed endemic species (41) as well as *M. calpeiana*.

**New information:**

The 43 assessed species belong to 15 families, the richest being Zodariidae, Dysderidae, Linyphiidae and Gnaphosidae. In general and despite the lack of information on more than half the species, general patterns and trends could be found.

Only 18 species (including *M. calpeiana* and *A. ataecina*) had enough data to allow their EOO (extent of occurrence) and AOO (area of occurrence) to be quantified. Of these, we modelled the distribution of 14 epigean species, eight of which were found to be widespread. The remaining six fulfilled at least one of the criteria for threatened species. Four species are troglobiont, all of which meet the EOO and AOO thresholds for threatened species. The remaining 25 Portuguese endemics had no reliable information on their range. Only nine species out of the 43 are estimated to be in decline and 11 are stable, with the majority of species having no information on trends (23 species).

Forest areas, sand dunes, shrublands and caves host the majority of species. As such, the threats to Portuguese endemics reflect the diversity of habitats they occupy. Urbanisation and climate change seem to be the most important threats to these species, although other factors are also important and represented across the data.

A considerable proportion of the currently known Portuguese endemic species can be found in national protected areas, with higher prominence to the Serras de Aire e Candeeiros, Douro Internacional, Vale do Guadiana, Sudoeste Alentejano e Costa Vicentina and Arrábida Natural Parks. These correspond mostly to areas that have been particularly well sampled during the last two decades.

## Introduction

Portugal is a small country with a large coastal area that occupies the majority of the western coast of the Iberian Peninsula and is separated into two biogeographic regions, Mediterranean and Atlantic (European Environment Agency 2017). While there are still a few relatively pristine areas, the country is mostly covered by a permanently changing landscape. Portugal is currently investing heavily in tourism, one of the causes of unregulated urbanisation and coastal development, factors that lead to fragmentation and loss of coastal habitats (Freire et al. 2009). Wildfires are a significant threat over an increasingly long dry season, a factor that, although natural and long standing, has been exacerbated in recent years by both poor land management and climate change (IPCC 2014), posing a challenge to invertebrate conservation (Pryke and Samways 2011). Finally, unsustainable land use management practices further threaten the stability of the country's ecosystems and long term co-existence between man and nature through activities that lead to soil erosion and introduction of exotic species (Nunes et al. 2011).

Despite ongoing disturbances, many of Portugal's natural characteristics still contribute towards a rich, unique fauna, spiders included (Arachnida: Araneae). The latest data show that 1488 spider species are known to occur in the Iberian Peninsula, of which 825 of them are present in mainland Portugal, 42 of those considered endemic to the country (Branco et al. 2019). While much of the Mediterranean and the Iberian Peninsula is considered a biodiversity hotspot, Portugal stands out for having a greater percentage of newly recorded endemic species (in the last decade) than Spain, despite representing only 15% of all new records for the Iberian Peninsula. The province of Faro in particular, Portugal's southernmost province and one of the country's most urbanised regions, at least along the coastal areas, is the province with the highest richness of new endemic species found during the last decade (16) as well as the province with the second highest richness (39) of Iberian endemics (after Illes Balears).

Portugal's duality as both highly speciose and highly under pressure along most of its territory makes it imperative to conduct studies that bridge both the gaps in our knowledge of spiders, as well as those between researchers and decision-makers. Both are being tentatively reduced by initiatives such as the Iberian Spider checklist and catalogue (Branco et al. 2019, Morano et al. 2019) and now through the conservation status assessments of all national endemics. Of the 42 Portuguese endemic species, only one, the troglobiont *Anapistula ataecina* Cardoso & Scharff, 2009, has been assessed according to the IUCN Red List criteria (Cardoso 2010). In addition to endemics, the only Iberian species protected by law in the mainland (Portuguese Republic 1999, Anex B-IV), *Macrothele calpeiana*, is also assessed, given its legal status.

## Methods

All analyses were computed in R (version 3.5.1) using the package "red - IUCN redlisting tools" (Cardoso 2017), with the record data from published papers, grey literature and other databases, all compiled in the Iberian Spider Catalogue (Morano et al. 2019). This package performs a number of spatial analyses, based on either observed occurences or estimated ranges, some of which are needed for correctly following the IUCN Red List criteria. Its functions include calculating the Extent of Occurrence (EOO), Area of Occupancy (AOO), mapping species ranges, species distribution modelling using climate and land cover, calculating the Red List Index for groups of species, amongst others. The calculation of confidence limits is possible for all measures (see further details below). Maps and data on geographical ranges, elevation and others can be exported in a variety of formats used for the assessments themselves, as well as visual presentation and safe-keeping.

Species with less than five georeferrenced records were considered to be insufficiently known and classified as Data Deficient (DD). When it was possible to reliably due so, i.e. for species with sufficient distribution data, EOO and AOO were calculated in one of two ways:

1. For troglobiont species, we assumed that we knew well enough the full range of the species, based on the fact that Portuguese cave systems are relatively well explored. We then classified these values as observed and used our occurrence records to:

- Calculate EOO by building a minimum convex polygon that encompassed all observations.
- Calculate AOO by summing the area of all 2 x 2 km cells known to be occupied.

2. For non-troglobiont species with at least five records, species distribution modelling (SDM) was performed.

This was done using the environmental data present in Worldclim 2.0 (Fick and Hijmans 2017) and the function map.sdm in the R package red to build ensemble models (Breiner et al. 2018, Lomba et al. 2010). One hundred models were run per species using both coordinates and the associated spatial error. This ensemble modelling was made with the Maxent method (Phillips et al. 2006). No variable subsets were used. Normally, only a subset of two variables from the total set would be used for better output predictions in rarer species as it mitigates overfitting (Breiner et al. 2018, Lomba et al. 2010), but it was found during execution that this resulted in possible overestimations of the range. Following the precautionary principle, we opted to use the full set of variables, even if risking overfitting for some species. Ensembles were weighted-summed using the Area Under the Curve (AUC) values to weight each of the 100 runs as:

weight_run_ = max(0, (AUC_run_ - 0.5))^2^

These probabilistic models were then processed with the map.habitat function, which further restricted them to patches, including observation points, thus often reducing the range and consequently the EOO and AOO values. We present for each assessment the EOO and AOO for both the consensus maps and their lower confidence limits (in reverse order in the assessments themselves), calculated respectively as the areas found suitable in at least 50% and 97.5% of the 100 models created per species (after weighting of individual models). All final maps and values were checked and validated by our own expert opinion. All data, presented in the Results section, use the lower confidence limits as per the precautionary principle and includes the previously assessed *Anapistula ataecina*.

## Species Conservation Profiles

### Eratigena barrientosi

#### Species information

Scientific name: Eratigena barrientosi

Species authority: Bolzern, Crespo & Cardoso, 2009

Common names: Funileira-de-Barrientos

Kingdom: Animalia

Phylum: Arthropoda

Class: Arachnida

Order: Araneae

Family: Agelenidae

Region for assessment: Global

#### Geographic range

Biogeographic realm: Palearctic

Countries: Portugal

Map of records (Google Earth): Suppl. material 1

Basis of EOO and AOO: Unknown

Basis (narrative): There are only two records for the species (Bolzern et al. 2009). The true range is therefore unknown and not possible to model with confidence.

Min Elevation/Depth (m): 50

Max Elevation/Depth (m): 320

Range description: This spider is known from only two sites in central Portugal (Bolzern et al. 2009), one in the University of Coimbra's Botanical Garden, Coimbra and the other one in Bairro, Santarém.

#### New occurrences

#### Extent of occurrence

EOO (km2): Unknown

Trend: Unknown

Causes ceased?: Unknown

Causes understood?: Unknown

Causes reversible?: Unknown

Extreme fluctuations?: Unknown

#### Area of occupancy

Trend: Unknown

Causes ceased?: Unknown

Causes understood?: Unknown

Causes reversible?: Unknown

Extreme fluctuations?: Unknown

AOO (km2): Unknown

#### Locations

Number of locations: Unknown

Justification for number of locations: Data available (2 records) are not enough to estimate the number of locations.

Trend: Unknown

Extreme fluctuations?: Unknown

#### Population

Number of individuals: Unknown

Trend: Unknown

Causes ceased?: Unknown

Causes understood?: Unknown

Causes reversible?: Unknown

Extreme fluctuations?: No

Population Information (Narrative): No estimates of population size exist.

#### Subpopulations

Number of subpopulations: Unknown

Trend: Unknown

Justification for trend: Data available (2 records) are not enough to estimate the number of subpopulations.

Extreme fluctuations?: No

Severe fragmentation?: Unknown

#### Habitat

System: Terrestrial

Habitat specialist: Unknown

Habitat (narrative): This spider is known from only two sites, one a botanical garden and the other a pinewood plantation.

Trend in extent, area or quality?: Unknown

##### Habitat

Habitat importance: Suitable

Habitats: 14.3. Artificial/Terrestrial - Plantations14.4. Artificial/Terrestrial - Rural Gardens16. Introduced vegetation

#### Habitat

Habitat importance: Suitable

Habitats: 14.3. Artificial/Terrestrial - Plantations14.4. Artificial/Terrestrial - Rural Gardens16. Introduced vegetation

#### Ecology

Size: 2.32 - 3.32 mm

Generation length (yr): 1

Dependency of single sp?: No

Ecology and traits (narrative): A ground-dwelling species that builds a sheet web to catch a variety of small prey. They also build small tube web retreats at one end of the sheet. Given the habitat types where the species was found, it seems to be tolerant to humans.

#### Threats

Justification for threats: Existence of threats is unknown for this species.

##### Threats

Threat type: Past

Threats: 12. Other options - Other threat

#### Threats

Threat type: Past

Threats: 12. Other options - Other threat

#### Conservation

Justification for conservation actions: This spider was collected at two sites, one of which makes it fair to assume that its true range might be completely or at least partially covered by the Natura 2000 network (PTCON0015) and the Serras de Aires e Candeeiros Natural Park.

##### Conservation actions

Conservation action type: In Place

Conservation actions: 1.1. Land/water protection - Site/area protection1.2. Land/water protection - Resource & habitat protection

#### Conservation actions

Conservation action type: In Place

Conservation actions: 1.1. Land/water protection - Site/area protection1.2. Land/water protection - Resource & habitat protection

#### Other

##### Use and trade

Use type: International

##### Ecosystem services

Ecosystem service type: Less important

##### Research needed

Research needed: 1.2. Research - Population size, distribution & trends1.3. Research - Life history & ecology1.5. Research - Threats

Justification for research needed: Research is needed on basic information such as distribution, ecology, life cycle and possible threats throughout the range.

#### Use and trade

Use type: International

#### Ecosystem services

Ecosystem service type: Less important

#### Research needed

Research needed: 1.2. Research - Population size, distribution & trends1.3. Research - Life history & ecology1.5. Research - Threats

Justification for research needed: Research is needed on basic information such as distribution, ecology, life cycle and possible threats throughout the range.

#### Viability analysis

### Eratigena incognita

#### Species information

Scientific name: Eratigena incognita

Species authority: Bolzern, Crespo & Cardoso, 2009

Common names: Funileira-incógnita

Kingdom: Animalia

Phylum: Arthropoda

Class: Arachnida

Order: Araneae

Family: Agelenidae

Region for assessment: Global

#### Geographic range

Biogeographic realm: Palearctic

Countries: Portugal

Map of records (Google Earth): Suppl. material 2

Basis of EOO and AOO: Unknown

Basis (narrative): Largely unknown, as there is only one record for the species (Bolzern et al. 2009). The species' true range is therefore unknown and not possible to model with confidence.

Min Elevation/Depth (m): 200

Max Elevation/Depth (m): 200

Range description: This spider is known from only one heavily urbanised mixed forest in Parque Florestal de Monsanto, close to Lisbon (Bolzern et al. 2009).

#### New occurrences

#### Extent of occurrence

EOO (km2): Unknown

Trend: Unknown

Causes ceased?: Unknown

Causes understood?: Unknown

Causes reversible?: Unknown

Extreme fluctuations?: Unknown

#### Area of occupancy

Trend: Unknown

Causes ceased?: Unknown

Causes understood?: Unknown

Causes reversible?: Unknown

Extreme fluctuations?: Unknown

AOO (km2): Unknown

#### Locations

Number of locations: Unknown

Justification for number of locations: Data available (1 record) are not enough to estimate the number of locations.

Trend: Unknown

Extreme fluctuations?: Unknown

#### Population

Number of individuals: Unknown

Trend: Unknown

Causes ceased?: Unknown

Causes understood?: Unknown

Causes reversible?: Unknown

Extreme fluctuations?: No

Population Information (Narrative): No estimates of population size exist.

#### Subpopulations

Number of subpopulations: Unknown

Trend: Unknown

Justification for trend: Data available (1 record) are not enough to estimate the number of subpopulations.

Extreme fluctuations?: Unknown

Severe fragmentation?: Unknown

#### Habitat

System: Terrestrial

Habitat specialist: Unknown

Habitat (narrative): This spider's only record comes from Parque Florestal de Monsanto, a small forest area that is isolated by human infrastructure, the closest natural area being located in Sintra, ca. 20 km away.

Trend in extent, area or quality?: Unknown

##### Habitat

Habitat importance: Major Importance

Habitats: 1.4. Forest - Temperate14.4. Artificial/Terrestrial - Rural Gardens14.5. Artificial/Terrestrial - Urban Areas

#### Habitat

Habitat importance: Major Importance

Habitats: 1.4. Forest - Temperate14.4. Artificial/Terrestrial - Rural Gardens14.5. Artificial/Terrestrial - Urban Areas

#### Ecology

Size: 2.04 mm

Generation length (yr): 1

Dependency of single sp?: No

Ecology and traits (narrative): A ground-dwelling species that builds a sheet web to catch a variety of small prey. They also build small tube web retreats at one end of the sheet.

#### Threats

Justification for threats: The existence of threats is unknown for this species.

##### Threats

Threat type: Past

Threats: 12. Other options - Other threat

#### Threats

Threat type: Past

Threats: 12. Other options - Other threat

#### Conservation

Justification for conservation actions: This spider has not been recorded inside or adjacent to protected areas. More records are needed in order to confirm or disprove this for the species' true range.

##### Conservation actions

Conservation action type: Needed

Conservation actions: 1.1. Land/water protection - Site/area protection1.2. Land/water protection - Resource & habitat protection

#### Conservation actions

Conservation action type: Needed

Conservation actions: 1.1. Land/water protection - Site/area protection1.2. Land/water protection - Resource & habitat protection

#### Other

##### Use and trade

Use type: International

##### Ecosystem services

Ecosystem service type: Very important

##### Research needed

Research needed: 1.2. Research - Population size, distribution & trends1.3. Research - Life history & ecology1.5. Research - Threats

Justification for research needed: Research is needed on basic information such as distribution, ecology, life cycle and possible threats throughout the range.

#### Use and trade

Use type: International

#### Ecosystem services

Ecosystem service type: Very important

#### Research needed

Research needed: 1.2. Research - Population size, distribution & trends1.3. Research - Life history & ecology1.5. Research - Threats

Justification for research needed: Research is needed on basic information such as distribution, ecology, life cycle and possible threats throughout the range.

#### Viability analysis

### Malthonica oceanica

#### Species information

Scientific name: Malthonica oceanica

Species authority: Barrientos & Cardoso, 2007

Common names: Tecedeira-de-funil-do-litoral

Kingdom: Animalia

Phylum: Arthropoda

Class: Arachnida

Order: Araneae

Family: Agelenidae

Region for assessment: Global

#### Geographic range

Biogeographic realm: Palearctic

Countries: Portugal

Map of records (Google Earth): Suppl. material 3

Basis of EOO and AOO: Species Distribution Model

Basis (narrative): Multiple collection sites are recorded for this species (20 records), mostly recent and in sand dunes (Barrientos and Cardoso 2007, Crespo et al. 2009, Crespo et al. 2010, Carvalho et al. 2011, Lissner 2017b). It was possible to perform species distribution modelling to predict its potential range with confidence limits. See methods for details.

Min Elevation/Depth (m): 0

Max Elevation/Depth (m): 569

Range description: This spider has been recorded in coastal areas all across Portugal, from its southernmost record in Monchique to its northernmost in Viana do Castelo (Barrientos and Cardoso 2007, Crespo et al. 2009, Crespo et al. 2010, Carvalho et al. 2011, Lissner 2017b).

#### New occurrences

#### Extent of occurrence

EOO (km2): 68995 - 98036

Trend: Stable

Justification for trend: There are no currently known threats to the species.

Causes ceased?: Yes

Causes understood?: Yes

Causes reversible?: Yes

Extreme fluctuations?: No

#### Area of occupancy

Trend: Stable

Justification for trend: There are no currently known threats to the species.

Causes ceased?: Yes

Causes understood?: Yes

Causes reversible?: Yes

Extreme fluctuations?: No

AOO (km2): 30080 - 48628

#### Locations

Number of locations: Not applicable

Justification for number of locations: There are no currently known threats to the species.

Trend: Stable

Extreme fluctuations?: Unknown

#### Population

Number of individuals: Unknown

Trend: Stable

Justification for trend: There are no currently known threats to the species.

Causes ceased?: Yes

Causes understood?: Yes

Causes reversible?: Yes

Extreme fluctuations?: No

Population Information (Narrative): No estimates of population size exist.

#### Subpopulations

Number of subpopulations: Unknown

Trend: Stable

Justification for trend: There are no currently known threats to the species.

Extreme fluctuations?: No

Severe fragmentation?: No

#### Habitat

System: Terrestrial

Habitat specialist: No

Habitat (narrative): Recorded in sand dunes, heathlands, rocky steppes, oak forests (*Quercus* spp.) and plantations (*Eucalyptus* sp., *Pinus* spp.) from north and central Portugal, occupying coastal or sometimes mountainous regions throughout the country. Recorded once in a marshland.

Trend in extent, area or quality?: Stable

Justification for trend: There are no currently known major threats to the species' habitat.

##### Habitat

Habitat importance: Major Importance

Habitats: 1.4. Forest - Temperate5.4. Wetlands (inland) - Bogs, Marshes, Swamps, Fens, Peatlands13.3. Marine Coastal/Supratidal - Coastal Sand Dunes

##### Habitat

Habitat importance: Suitable

Habitats: 16. Introduced vegetation

#### Habitat

Habitat importance: Major Importance

Habitats: 1.4. Forest - Temperate5.4. Wetlands (inland) - Bogs, Marshes, Swamps, Fens, Peatlands13.3. Marine Coastal/Supratidal - Coastal Sand Dunes

#### Habitat

Habitat importance: Suitable

Habitats: 16. Introduced vegetation

#### Ecology

Size: 3.27 - 4.64 mm

Generation length (yr): 1

Dependency of single sp?: No

Ecology and traits (narrative): This spider is a ground-dwelling species found in areas with relatively dense vegetation cover. It builds a sheet web often under the leaf litter and eats a variety of small crawling invertebrates.

#### Threats

Justification for threats: The existence of threats is unknown for this species.

##### Threats

Threat type: Past

Threats: 12. Other options - Other threat

#### Threats

Threat type: Past

Threats: 12. Other options - Other threat

#### Conservation

Justification for conservation actions: This spider has been found in a variety of protected areas, namely the Litoral Norte Natural Park, the Paul de Arzila Natural Reserve, the Serras de Aire e Candeeiros Natural Park, the Paul do Boquilobo Natural Reserve and the Serra de São Mamede Natural Park. Given how widespread the species distribution modelling seems to predict this species to be, it is not unreasonable to assume that it may occupy further protected areas, as well as a variety of areas covered by the Natura 2000 network (most distinct being Monchique, its southernmost tip).

##### Conservation actions

Conservation action type: In Place

Conservation actions: 1.1. Land/water protection - Site/area protection1.2. Land/water protection - Resource & habitat protection

#### Conservation actions

Conservation action type: In Place

Conservation actions: 1.1. Land/water protection - Site/area protection1.2. Land/water protection - Resource & habitat protection

#### Other

##### Use and trade

Use type: International

##### Ecosystem services

Ecosystem service type: Very important

##### Research needed

Research needed: 1.5. Research - Threats3.1. Monitoring - Population trends3.4. Monitoring - Habitat trends

Justification for research needed: Monitoring of population and habitat are important to confirm inferred trends.

#### Use and trade

Use type: International

#### Ecosystem services

Ecosystem service type: Very important

#### Research needed

Research needed: 1.5. Research - Threats3.1. Monitoring - Population trends3.4. Monitoring - Habitat trends

Justification for research needed: Monitoring of population and habitat are important to confirm inferred trends.

#### Viability analysis

### Dysdera alentejana

#### Species information

Scientific name: Dysdera alentejana

Species authority: Ferrández, 1996

Common names: Aranha-tenaz-alentejana

Kingdom: Animalia

Phylum: Arthropoda

Class: Arachnida

Order: Araneae

Family: Dysderidae

Region for assessment: Global

#### Geographic range

Biogeographic realm: Palearctic

Countries: Portugal

Map of records (Google Earth): Suppl. material 4

Basis of EOO and AOO: Species Distribution Model

Basis (narrative): Multiple collection sites are recorded for this species (10 records), mostly recent and in a variety of habitats (Ferrández 1996, Cardoso 2004, Cardoso et al. 2009). It was possible to perform species distribution modelling to predict its potential range with confidence limits. See Methods for details.

Min Elevation/Depth (m): 0

Max Elevation/Depth (m): 856

Range description: This spider has been recorded several times, all of them in the province of Beja, Alentejo. Nevertheless, species distribution modelling predicts that it might be widespread throughout the southwest of the Iberian Peninsula.

#### New occurrences

#### Extent of occurrence

EOO (km2): 71231 - 82399

Trend: Stable

Justification for trend: There are no currently known threats to the species.

Causes ceased?: Yes

Causes understood?: Yes

Causes reversible?: Yes

Extreme fluctuations?: No

#### Area of occupancy

Trend: Stable

Justification for trend: There are no currently known threats to the species.

Causes ceased?: Yes

Causes understood?: Yes

Causes reversible?: Yes

Extreme fluctuations?: No

AOO (km2): 39548 - 47804

#### Locations

Number of locations: Not applicable

Justification for number of locations: There are no currently known threats to the species.

Trend: Stable

Extreme fluctuations?: Unknown

#### Population

Number of individuals: Unknown

Trend: Stable

Justification for trend: There are no currently known threats to the species.

Causes ceased?: Yes

Causes understood?: Yes

Causes reversible?: Yes

Extreme fluctuations?: No

Population Information (Narrative): No estimates of population size exist.

#### Subpopulations

Number of subpopulations: Unknown

Trend: Stable

Justification for trend: There are no currently known threats to the species.

Extreme fluctuations?: No

Severe fragmentation?: No

#### Habitat

System: Terrestrial

Habitat specialist: No

Habitat (narrative): This spider is found in a variety of habitats, from oak forests (*Quercus* spp.) to shrublands (*Cystus* sp., *Juniperus* sp.) and plantations (*Eucalyptus* sp., *Pinus* spp.).

Trend in extent, area or quality?: Stable

Justification for trend: There are no currently known major threats to the species' habitat.

##### Habitat

Habitat importance: Major Importance

Habitats: 1.4. Forest - Temperate3.8. Shrubland - Mediterranean-type Shrubby Vegetation

##### Habitat

Habitat importance: Suitable

Habitats: 16. Introduced vegetation

#### Habitat

Habitat importance: Major Importance

Habitats: 1.4. Forest - Temperate3.8. Shrubland - Mediterranean-type Shrubby Vegetation

#### Habitat

Habitat importance: Suitable

Habitats: 16. Introduced vegetation

#### Ecology

Size: 4.2 mm

Generation length (yr): 1

Dependency of single sp?: Unknown

Ecology and traits (narrative): This spider is a ground-dwelling species that builds no web, actively hunting for the woodlice of which it is presumably a specialist.

#### Threats

Justification for threats: The existence of threats is unknown for this species.

##### Threats

Threat type: Past

Threats: 12. Other options - Other threat

#### Threats

Threat type: Past

Threats: 12. Other options - Other threat

#### Conservation

Justification for conservation actions: The vast majority of this species' records are located inside the Vale do Guadiana Natural Park, which is covered by the Natura 2000 network (PTCON0036; PTZPE0045; PTZPE0047).

##### Conservation actions

Conservation action type: In Place

Conservation actions: 1.1. Land/water protection - Site/area protection1.2. Land/water protection - Resource & habitat protection

#### Conservation actions

Conservation action type: In Place

Conservation actions: 1.1. Land/water protection - Site/area protection1.2. Land/water protection - Resource & habitat protection

#### Other

##### Use and trade

Use type: International

##### Ecosystem services

Ecosystem service type: Very important

##### Research needed

Research needed: 3.1. Monitoring - Population trends3.4. Monitoring - Habitat trends

Justification for research needed: Monitoring of population and habitat are important to confirm inferred trends.

#### Use and trade

Use type: International

#### Ecosystem services

Ecosystem service type: Very important

#### Research needed

Research needed: 3.1. Monitoring - Population trends3.4. Monitoring - Habitat trends

Justification for research needed: Monitoring of population and habitat are important to confirm inferred trends.

#### Viability analysis

### Harpactea algarvensis

#### Species information

Scientific name: Harpactea algarvensis

Species authority: Ferrández, 1990

Common names: Aranha-nómada-do-Algarve

Kingdom: Animalia

Phylum: Arthropoda

Class: Arachnida

Order: Araneae

Family: Dysderidae

Region for assessment: Global

#### Geographic range

Biogeographic realm: Palearctic

Countries: Portugal

Map of records (Google Earth): Suppl. material 5

Basis of EOO and AOO: Unknown

Basis (narrative): Largely unknown as there is only one record. The true range is therefore unknown and not possible to model with confidence.

Min Elevation/Depth (m): 500

Max Elevation/Depth (m): 500

Range description: Largely unknown as there is only one record (Ferrández 1990) for the species in the small village of Barranco do Velho, Faro, in an unspecified habitat.

#### New occurrences

#### Extent of occurrence

EOO (km2): Unknown

Trend: Unknown

Causes ceased?: Unknown

Causes understood?: Unknown

Causes reversible?: Unknown

Extreme fluctuations?: Unknown

#### Area of occupancy

Trend: Unknown

Causes ceased?: Unknown

Causes understood?: Unknown

Causes reversible?: Unknown

Extreme fluctuations?: Unknown

AOO (km2): Unknown

#### Locations

Number of locations: Unknown

Justification for number of locations: The data available (a single record) are not enough to estimate the number of locations.

Trend: Unknown

Extreme fluctuations?: Unknown

#### Population

Number of individuals: Unknown

Trend: Unknown

Causes ceased?: Unknown

Causes understood?: Unknown

Causes reversible?: Unknown

Extreme fluctuations?: No

Population Information (Narrative): No estimates of population size exist.

#### Subpopulations

Number of subpopulations: Unknown

Trend: Unknown

Justification for trend: The data available (a single record) are not enough to estimate the number of subpopulations.

Extreme fluctuations?: Unknown

Severe fragmentation?: Unknown

#### Habitat

System: Terrestrial

Habitat specialist: Unknown

Habitat (narrative): This spider is known from one site of unspecified habitat.

Trend in extent, area or quality?: Unknown

##### Habitat

Habitat importance: Major Importance

Habitats: 18. Unknown

#### Habitat

Habitat importance: Major Importance

Habitats: 18. Unknown

#### Ecology

Size: 4.6 mm

Generation length (yr): 1

Dependency of single sp?: No

Ecology and traits (narrative): A nocturnal ground-dwelling species which produces no web and eats a variety of small invertebrates.

#### Threats

Justification for threats: The existence of threats is unknown for this species.

##### Threats

Threat type: Past

Threats: 12. Other options - Other threat

#### Threats

Threat type: Past

Threats: 12. Other options - Other threat

#### Conservation

Justification for conservation actions: It is unknown exactly where this spider was collected, but it is fair to assume that its true range might be partially or completely covered by the Natura 2000 network (PTCON0057).

##### Conservation actions

Conservation action type: In Place

Conservation actions: 1.1. Land/water protection - Site/area protection1.2. Land/water protection - Resource & habitat protection

#### Conservation actions

Conservation action type: In Place

Conservation actions: 1.1. Land/water protection - Site/area protection1.2. Land/water protection - Resource & habitat protection

#### Other

##### Use and trade

Use type: International

##### Ecosystem services

Ecosystem service type: Very important

##### Research needed

Research needed: 1.2. Research - Population size, distribution & trends1.3. Research - Life history & ecology1.5. Research - Threats

Justification for research needed: Research is needed on basic information such as distribution, ecology, life cycle and possible threats throughout the range.

#### Use and trade

Use type: International

#### Ecosystem services

Ecosystem service type: Very important

#### Research needed

Research needed: 1.2. Research - Population size, distribution & trends1.3. Research - Life history & ecology1.5. Research - Threats

Justification for research needed: Research is needed on basic information such as distribution, ecology, life cycle and possible threats throughout the range.

#### Viability analysis

### Harpactea magnibulbi

#### Species information

Scientific name: Harpactea magnibulbi

Species authority: Machado & Ferrández, 1991

Common names: Aranha-nómada

Kingdom: Animalia

Phylum: Arthropoda

Class: Arachnida

Order: Araneae

Family: Dysderidae

Region for assessment: Global

#### Geographic range

Biogeographic realm: Palearctic

Countries: Portugal

Map of records (Google Earth): Suppl. material 6

Basis of EOO and AOO: Unknown

Basis (narrative): Largely unknown, as there are only four records for the species (Machado and Ferrández 1991).

Min Elevation/Depth (m): 50

Max Elevation/Depth (m): 800

Range description: This spider is known from only four sites in Algarve, Southernmost Portugal (Machado and Ferrández 1991). Its true range is however unknown and not possible to model with confidence given the scarcity of records.

#### New occurrences

#### Extent of occurrence

EOO (km2): Unknown

Trend: Unknown

Causes ceased?: Unknown

Causes understood?: Unknown

Causes reversible?: Unknown

Extreme fluctuations?: No

#### Area of occupancy

Trend: Unknown

Causes ceased?: Unknown

Causes understood?: Unknown

Causes reversible?: Unknown

Extreme fluctuations?: No

AOO (km2): Unknown

#### Locations

Number of locations: Unknown

Justification for number of locations: The data available (four records) are not enough to estimate the number of locations.

Trend: Unknown

Extreme fluctuations?: Unknown

#### Population

Number of individuals: Unknown

Trend: Unknown

Causes ceased?: Unknown

Causes understood?: Unknown

Causes reversible?: Unknown

Extreme fluctuations?: No

Population Information (Narrative): No estimates of population size exist.

#### Subpopulations

Number of subpopulations: Unknown

Trend: Unknown

Justification for trend: The data available (four records) are not enough to estimate the number of subpopulations.

Extreme fluctuations?: Unknown

Severe fragmentation?: Unknown

#### Habitat

System: Terrestrial

Habitat specialist: No

Habitat (narrative): This spider has been recorded at four different sites, one of them a cave. Remaining sites possess no habitat information.

Trend in extent, area or quality?: Unknown

##### Habitat

Habitat importance: Major Importance

Habitats: 7.1. Caves and Subterranean Habitats (non-aquatic) - Caves

#### Habitat

Habitat importance: Major Importance

Habitats: 7.1. Caves and Subterranean Habitats (non-aquatic) - Caves

#### Ecology

Size: 3.71 - 6.16 mm

Generation length (yr): 1

Dependency of single sp?: No

Ecology and traits (narrative): A nocturnal ground-dwelling species that produces no web and eats a variety of small invertebrates.

#### Threats

Justification for threats: The existence of threats is unknown for this species.

##### Threats

Threat type: Past

Threats: 12. Other options - Other threat

#### Threats

Threat type: Past

Threats: 12. Other options - Other threat

#### Conservation

Justification for conservation actions: This spider has not been recorded within or adjacent to national protected areas. However, the species' true range might be totally or at least partially covered by the Natura 2000 network (PTCON0037; PTCON0049).

##### Conservation actions

Conservation action type: In Place

Conservation actions: 1.1. Land/water protection - Site/area protection1.2. Land/water protection - Resource & habitat protection

#### Conservation actions

Conservation action type: In Place

Conservation actions: 1.1. Land/water protection - Site/area protection1.2. Land/water protection - Resource & habitat protection

#### Other

##### Use and trade

Use type: International

##### Ecosystem services

Ecosystem service type: Very important

##### Research needed

Research needed: 1.2. Research - Population size, distribution & trends1.3. Research - Life history & ecology1.5. Research - Threats

Justification for research needed: Research is needed on basic information such as distribution, ecology, life cycle and possible threats throughout the range.

#### Use and trade

Use type: International

#### Ecosystem services

Ecosystem service type: Very important

#### Research needed

Research needed: 1.2. Research - Population size, distribution & trends1.3. Research - Life history & ecology1.5. Research - Threats

Justification for research needed: Research is needed on basic information such as distribution, ecology, life cycle and possible threats throughout the range.

#### Viability analysis

### Harpactea proxima

#### Species information

Scientific name: Harpactea proxima

Species authority: Ferrández, 1990

Common names: Aranha-nómada

Kingdom: Animalia

Phylum: Arthropoda

Class: Arachnida

Order: Araneae

Family: Dysderidae

Region for assessment: Global

#### Geographic range

Biogeographic realm: Palearctic

Countries: Portugal

Map of records (Google Earth): Suppl. material 7

Basis of EOO and AOO: Species Distribution Model

Basis (narrative): Multiple collection sites are recorded for this species (four records), mostly recent but mostly without habitat information (Ferrández 1990, Cardoso 2004). It was possible to perform species distribution modelling to predict its potential range with confidence limits, albeit with great uncertainty. See methods for details.

Min Elevation/Depth (m): 0

Max Elevation/Depth (m): 169

Range description: This spider is known from only four sites in Beja and Setúbal in south Portugal. The species distribution model predicts it might be restricted to this region.

#### New occurrences

#### Extent of occurrence

EOO (km2): 5022 - 6864

Trend: Stable

Justification for trend: There are no currently known threats to the species.

Causes ceased?: Yes

Causes understood?: Yes

Causes reversible?: Yes

Extreme fluctuations?: No

#### Area of occupancy

Trend: Stable

Justification for trend: There are no currently known threats to the species.

Causes ceased?: Yes

Causes understood?: Yes

Causes reversible?: Yes

Extreme fluctuations?: No

AOO (km2): 1648 - 3088

#### Locations

Number of locations: Unknown

Justification for number of locations: There are no currently known threats to the species.

Trend: Unknown

Extreme fluctuations?: Unknown

#### Population

Number of individuals: Unknown

Trend: Stable

Justification for trend: There are no currently known threats to the species.

Causes ceased?: Yes

Causes understood?: Yes

Causes reversible?: Yes

Extreme fluctuations?: No

Population Information (Narrative): No estimates of population size exist.

#### Subpopulations

Number of subpopulations: Unknown

Trend: Stable

Justification for trend: There are no currently known threats to the species.

Extreme fluctuations?: Unknown

Severe fragmentation?: Unknown

#### Habitat

System: Terrestrial

Habitat specialist: Unknown

Habitat (narrative): This spider is known from four sites, only two of these possessing habitat information. The species seems to be associated with *Quercus* spp. and *Olea* sp.

Trend in extent, area or quality?: Unknown

##### Habitat

Habitat importance: Major Importance

Habitats: 3.8. Shrubland - Mediterranean-type Shrubby Vegetation

#### Habitat

Habitat importance: Major Importance

Habitats: 3.8. Shrubland - Mediterranean-type Shrubby Vegetation

#### Ecology

Size: 3.4 - 3.9 mm

Generation length (yr): 1

Dependency of single sp?: No

Ecology and traits (narrative): A nocturnal ground-dwelling species that produces no web and eats a variety of small invertebrates.

#### Threats

Justification for threats: The existence of threats is unknown for this species.

##### Threats

Threat type: Past

Threats: 12. Other options - Other threat

#### Threats

Threat type: Past

Threats: 12. Other options - Other threat

#### Conservation

Justification for conservation actions: One of the species records is attributed to the outskirts of Mértola, inside the Vale do Guadiana Natural Park. A second record in Ponte de Serpa is close to the same park. Additionally, considering the location of *Harpactea proxima*'s records and the SDM, the species' true range might be partially covered by the Natura 2000 network (PTZPE0047, PTCON0036).

##### Conservation actions

Conservation action type: In Place

Conservation actions: 1.1. Land/water protection - Site/area protection1.2. Land/water protection - Resource & habitat protection

#### Conservation actions

Conservation action type: In Place

Conservation actions: 1.1. Land/water protection - Site/area protection1.2. Land/water protection - Resource & habitat protection

#### Other

##### Use and trade

Use type: International

##### Ecosystem services

Ecosystem service type: Very important

##### Research needed

Research needed: 1.5. Research - Threats3.1. Monitoring - Population trends3.4. Monitoring - Habitat trends

Justification for research needed: Monitoring of population and habitat are important to confirm inferred trends.

#### Use and trade

Use type: International

#### Ecosystem services

Ecosystem service type: Very important

#### Research needed

Research needed: 1.5. Research - Threats3.1. Monitoring - Population trends3.4. Monitoring - Habitat trends

Justification for research needed: Monitoring of population and habitat are important to confirm inferred trends.

#### Viability analysis

### Harpactea stalitoides

#### Species information

Scientific name: Harpactea stalitoides

Species authority: Ribera, 1993

Common names: Aranha-nómada-das-estalactites

Kingdom: Animalia

Phylum: Arthropoda

Class: Arachnida

Order: Araneae

Family: Dysderidae

Region for assessment: Global

#### Geographic range

Biogeographic realm: Palearctic

Countries: Portugal

Map of records (Google Earth): Suppl. material 8

Basis of EOO and AOO: Observed

Basis (narrative): Few collection sites are recorded for this species (four records), mostly recent and all of them in caves in the Algarve (Ribera 1993, Reboleira et al. 2011). Due to its nature as a troglobiont species and the fact that Portuguese caves are relatively well sampled, the known record points should accurately reflect reality.

Min Elevation/Depth (m): 38

Max Elevation/Depth (m): 308

Range description: This spider is known from four isolated caves in the Maciço Calcário do Algarve (MCA) in southern Portugal.

#### New occurrences

#### Extent of occurrence

EOO (km2): 1469

Trend: Decline (inferred)

Justification for trend: This spider currently faces threats of habitat loss due to urbanisation, land use change on the surface altering the microclimate beneath and stone quarries.

Causes ceased?: No

Causes understood?: Yes

Causes reversible?: No

Extreme fluctuations?: No

#### Area of occupancy

Trend: Decline (inferred)

Justification for trend: This spider currently faces threats of habitat loss due to urbanisation, land use change on the surface altering the microclimate beneath and stone quarries.

Causes ceased?: No

Causes understood?: Yes

Causes reversible?: No

Extreme fluctuations?: No

AOO (km2): 16

#### Locations

Number of locations: 4

Justification for number of locations: This spider is known from four isolated caves in the Maciço Calcário do Algarve (MCA) in southern Portugal. Current threats do not imply a difference between the number of locations and number of subpopulations.

Trend: Decline (inferred)

Extreme fluctuations?: No

#### Population

Number of individuals: Unknown

Trend: Decline (inferred)

Justification for trend: This spider currently faces threats of habitat loss due to urbanisation, land use change on the surface altering the microclimate beneath and stone quarries.

Basis for decline: (c) a decline in area of occupancy, extent of occurrence and/or quality of habitat

Causes ceased?: No

Causes understood?: Yes

Causes reversible?: No

Extreme fluctuations?: No

Population Information (Narrative): No estimates of population size exist.

#### Subpopulations

Number of subpopulations: 4

Trend: Decline (inferred)

Justification for trend: This spider currently faces threats of habitat loss due to urbanisation, land use change on the surface altering the microclimate beneath and stone quarries.

Extreme fluctuations?: No

Severe fragmentation?: Unknown

Justification for fragmentation: No estimates of population size exist.

#### Habitat

System: Terrestrial

Habitat specialist: Yes

Habitat (narrative): This spider possesses a highly restricted habitat as it is known from only four sites, all located in caves.

Trend in extent, area or quality?: Unknown

##### Habitat

Habitat importance: Major Importance

Habitats: 7.1. Caves and Subterranean Habitats (non-aquatic) - Caves

#### Habitat

Habitat importance: Major Importance

Habitats: 7.1. Caves and Subterranean Habitats (non-aquatic) - Caves

#### Ecology

Size: 3.04 mm

Generation length (yr): 1

Dependency of single sp?: Unknown

Ecology and traits (narrative): So far, this spider is the only known troglobiont species of its genus. Its eyes are totally absent as well as its pigmentation and it shows elongated appendages as are typical for many troglobionts (Ribera 1993).

#### Threats

Justification for threats: This spider currently faces threats of habitat loss due to urbanisation, land use change on the surface altering the microclimate beneath and stone quarries.

##### Threats

Threat type: Ongoing

Threats: 3.2. Energy production & mining - Mining & quarrying

#### Threats

Threat type: Ongoing

Threats: 3.2. Energy production & mining - Mining & quarrying

#### Conservation

Justification for conservation actions: The species range is partially covered by the Natura 2000 network (PTCON0049, PTCON0050).

##### Conservation actions

Conservation action type: In Place

Conservation actions: 1.1. Land/water protection - Site/area protection1.2. Land/water protection - Resource & habitat protection

#### Conservation actions

Conservation action type: In Place

Conservation actions: 1.1. Land/water protection - Site/area protection1.2. Land/water protection - Resource & habitat protection

#### Other

##### Use and trade

Use type: International

##### Ecosystem services

Ecosystem service type: Very important

##### Research needed

Research needed: 3.1. Monitoring - Population trends3.4. Monitoring - Habitat trends

Justification for research needed: Monitoring of population and habitat are important to confirm inferred trends.

#### Use and trade

Use type: International

#### Ecosystem services

Ecosystem service type: Very important

#### Research needed

Research needed: 3.1. Monitoring - Population trends3.4. Monitoring - Habitat trends

Justification for research needed: Monitoring of population and habitat are important to confirm inferred trends.

#### Viability analysis

### Harpactea subiasi

#### Species information

Scientific name: Harpactea subiasi

Species authority: Ferrández, 1990

Common names: Aranha-nómada

Kingdom: Animalia

Phylum: Arthropoda

Class: Arachnida

Order: Araneae

Family: Dysderidae

Region for assessment: Global

#### Geographic range

Biogeographic realm: Palearctic

Countries: Portugal

Map of records (Google Earth): Suppl. material 9

Basis of EOO and AOO: Species Distribution Model

Basis (narrative): Multiple collection sites are recorded for this species (seven records), mostly recent and in a variety of habitats. It was possible to perform species distribution modelling to predict its potential range with confidence limits. See Methods for details.

Min Elevation/Depth (m): 0

Max Elevation/Depth (m): 335

Range description: This spider has been recorded in southern Portugal along the coast (Ferrández 1990, Cardoso et al. 2008a, Carvalho et al. 2011, Lissner 2017b, Morano et al. 2019). The species distribution modelling predicts that the species could be widespread along this area.

#### New occurrences

#### Extent of occurrence

EOO (km2): 9611 - 19871

Trend: Stable

Justification for trend: There are no currently known threats to the species.

Causes ceased?: Yes

Causes understood?: Yes

Causes reversible?: Yes

Extreme fluctuations?: No

#### Area of occupancy

Trend: Stable

Justification for trend: There are no currently known threats to the species.

Causes ceased?: Yes

Causes understood?: Yes

Causes reversible?: Yes

Extreme fluctuations?: No

AOO (km2): 2652 - 5736

#### Locations

Number of locations: Not applicable

Justification for number of locations: There are no currently known threats to the species.

Trend: Stable

Extreme fluctuations?: Unknown

#### Population

Number of individuals: Unknown

Trend: Stable

Causes ceased?: Yes

Causes understood?: Yes

Causes reversible?: Yes

Extreme fluctuations?: No

Population Information (Narrative): No estimates of population size exist.

#### Subpopulations

Number of subpopulations: Unknown

Trend: Stable

Extreme fluctuations?: No

Severe fragmentation?: No

#### Habitat

System: Terrestrial

Habitat specialist: No

Habitat (narrative): The records of this spider encompass multiple habitats. The species has been recorded so far on sand dunes, mediterranean woods, rocky steppes, forests (*Quercus suber*) and one polje, a particular karstic habitat in Terras do Risco in the Arrábida Natural Park.

Trend in extent, area or quality?: Stable

Justification for trend: There are no currently known major threats to the species habitat.

##### Habitat

Habitat importance: Major Importance

Habitats: 1.4. Forest - Temperate4.4. Grassland - Temperate13.3. Marine Coastal/Supratidal - Coastal Sand Dunes

#### Habitat

Habitat importance: Major Importance

Habitats: 1.4. Forest - Temperate4.4. Grassland - Temperate13.3. Marine Coastal/Supratidal - Coastal Sand Dunes

#### Ecology

Size: 4 - 4.7 mm

Generation length (yr): 1

Dependency of single sp?: No

Ecology and traits (narrative): A nocturnal ground-dwelling species that produces no web and eats a variety of small invertebrates.

#### Threats

Justification for threats: The existence of threats is unknown for this species.

##### Threats

Threat type: Past

Threats: 12. Other options - Other threat

#### Threats

Threat type: Past

Threats: 12. Other options - Other threat

#### Conservation

Justification for conservation actions: The vast majority of this spider's records are located within protected areas: the Arrábida Natural Park and the Lagoas de Santo André e Sancha Natural Reserve. The species distribution modelling predicts that it could also be present in the Sudoeste Alentejano e Costa Vicentina Natural Park, Estuário do Sado Natural Park and Ria Formosa Natural Park. The sites, from where it has been recorded, are inside the Natura 2000 network (PTCON0010; PTCON0034; PTZPE0014; PTZPE0013) and it could be present in further protected areas.

##### Conservation actions

Conservation action type: In Place

Conservation actions: 1.1. Land/water protection - Site/area protection1.2. Land/water protection - Resource & habitat protection

#### Conservation actions

Conservation action type: In Place

Conservation actions: 1.1. Land/water protection - Site/area protection1.2. Land/water protection - Resource & habitat protection

#### Other

##### Use and trade

Use type: International

##### Ecosystem services

Ecosystem service type: Very important

##### Research needed

Research needed: 3.1. Monitoring - Population trends3.4. Monitoring - Habitat trends

Justification for research needed: Monitoring of population and habitat are important to confirm inferred trends.

#### Use and trade

Use type: International

#### Ecosystem services

Ecosystem service type: Very important

#### Research needed

Research needed: 3.1. Monitoring - Population trends3.4. Monitoring - Habitat trends

Justification for research needed: Monitoring of population and habitat are important to confirm inferred trends.

#### Viability analysis

### Adonea algarvensis

#### Species information

Scientific name: Adonea algarvensis

Species authority: Wunderlich, 2017

Kingdom: Animalia

Phylum: Arthropoda

Class: Arachnida

Order: Araneae

Family: Eresidae

Region for assessment: Global

#### Geographic range

Biogeographic realm: Palearctic

Countries: Portugal

Map of records (Google Earth): Suppl. material 10

Basis of EOO and AOO: Unknown

Basis (narrative): Multiple collection sites are recorded for this species (seven records) from both published sources (Wunderlich 2017) and our own data, all recent and in coastal dune vegetation. It was possible to perform species distribution modelling to predict its potential range with confidence limits. See Methods for details.

Min Elevation/Depth (m): 0

Max Elevation/Depth (m): 121

Range description: This spider is known exclusively from dune sites scattered across Portugal's southern coasts (Wunderlich 2017). The species distribution modelling predicts that the species' true range probably does not stray far from known occurrence records.

#### New occurrences

#### Extent of occurrence

EOO (km2): 1503 - 1798

Trend: Decline (inferred)

Justification for trend: The sand dunes, from where this species is exclusively found, are delicate habitats threatened by habitat loss due to urbanisation and possible increase in number of extreme weather events due to climate change.

Causes ceased?: No

Causes understood?: Yes

Causes reversible?: No

Extreme fluctuations?: No

#### Area of occupancy

Trend: Decline (inferred)

Justification for trend: The sand dunes, from where this species is exclusively found, are delicate habitats threatened by habitat loss due to urbanisation and possible increase in number of extreme weather events due to climate change.

Causes ceased?: No

Causes understood?: Yes

Causes reversible?: No

Extreme fluctuations?: No

AOO (km2): 204 - 480

#### Locations

Number of locations: Unknown

Justification for number of locations: The data available is not enough to estimate the number of locations.

Trend: Unknown

Extreme fluctuations?: No

#### Population

Number of individuals: Unknown

Trend: Decline (inferred)

Justification for trend: The sand dunes, from where this species is exclusively found, are delicate habitats threatened by habitat loss due to urbanisation and possible increase in number of extreme weather events due to climate change.

Basis for decline: (c) a decline in area of occupancy, extent of occurrence and/or quality of habitat

Causes ceased?: No

Causes understood?: Yes

Causes reversible?: No

Extreme fluctuations?: No

Population Information (Narrative): No estimates of population size exist.

#### Subpopulations

Number of subpopulations: Unknown

Trend: Decline (inferred)

Justification for trend: The data available is not enough to estimate the number of subpopulations.

Extreme fluctuations?: Unknown

Severe fragmentation?: No

#### Habitat

System: Terrestrial

Habitat specialist: Yes

Habitat (narrative): This spider is known exclusively from dunes, often consolidated or on top of cliffs.

Trend in extent, area or quality?: Decline (observed)

##### Habitat

Habitat importance: Major Importance

Habitats: 13.3. Marine Coastal/Supratidal - Coastal Sand Dunes

#### Habitat

Habitat importance: Major Importance

Habitats: 13.3. Marine Coastal/Supratidal - Coastal Sand Dunes

#### Ecology

Size: 6.5 - 7 mm

Generation length (yr): 4

Dependency of single sp?: No

Ecology and traits (narrative): This spider is a ground-dwelling, sheet-web builder that constructs a simple vertical or inclined burrow and feeds on various small arthropods.

#### Threats

Justification for threats: The sand dunes, from where this species is exclusively found, are delicate habitats threatened by habitat loss due to urbanisation and possible increase in number of extreme weather events due to climate change.

##### Threats

Threat type: Ongoing

Threats: 1.1. Residential & commercial development - Housing & urban areas1.2. Residential & commercial development - Commercial & industrial areas1.3. Residential & commercial development - Tourism & recreation areas11.4. Climate change & severe weather - Storms & flooding

#### Threats

Threat type: Ongoing

Threats: 1.1. Residential & commercial development - Housing & urban areas1.2. Residential & commercial development - Commercial & industrial areas1.3. Residential & commercial development - Tourism & recreation areas11.4. Climate change & severe weather - Storms & flooding

#### Conservation

Justification for conservation actions: The species range is partially covered by the Natura 2000 network (PTZPE0017; PTCON0012; PTCON0013). It is also partially covered by the Ria Formosa Natural Park and the Sudoeste Alentejano e Costa Vicentina Natural Park. Additionally, all beaches in Portugal are governed by the European Water Framework Directive (directive 2000/60/EC), being protected by means of land-use plans that preserve coastal ecosystems (decree-law Nº 130/2012).

##### Conservation actions

Conservation action type: In Place

Conservation actions: 1.1. Land/water protection - Site/area protection1.2. Land/water protection - Resource & habitat protection

#### Conservation actions

Conservation action type: In Place

Conservation actions: 1.1. Land/water protection - Site/area protection1.2. Land/water protection - Resource & habitat protection

#### Other

##### Use and trade

Use type: International

##### Ecosystem services

Ecosystem service type: Very important

##### Research needed

Research needed: 3.1. Monitoring - Population trends3.4. Monitoring - Habitat trends

Justification for research needed: Monitoring of population and habitat are important to confirm inferred trends.

#### Use and trade

Use type: International

#### Ecosystem services

Ecosystem service type: Very important

#### Research needed

Research needed: 3.1. Monitoring - Population trends3.4. Monitoring - Habitat trends

Justification for research needed: Monitoring of population and habitat are important to confirm inferred trends.

#### Viability analysis

### Filistata pygmaea

#### Species information

Scientific name: Filistata pygmaea

Species authority: Zonstein, Marusik & Grabolle 2018

Kingdom: Animalia

Phylum: Arthropoda

Class: Arachnida

Order: Araneae

Family: Filistatidae

Region for assessment: Global

#### Geographic range

Biogeographic realm: Palearctic

Countries: Portugal

Map of records (Google Earth): Suppl. material 11

Basis of EOO and AOO: Unknown

Basis (narrative): There is only one record for the species (Zonstein et al. 2018). Its true range is therefore unknown and not possible to model with confidence.

Min Elevation/Depth (m): 69

Max Elevation/Depth (m): 69

Range description: This spider is known from only one site, in the remnants of a paleodune field in a subcoastal region near Sagres, Algarve, southern Portugal (Zonstein et al. 2018).

#### New occurrences

#### Extent of occurrence

EOO (km2): Unknown

Trend: Unknown

Causes ceased?: Unknown

Causes understood?: Unknown

Causes reversible?: Unknown

Extreme fluctuations?: Unknown

#### Area of occupancy

Trend: Unknown

Causes ceased?: Unknown

Causes understood?: Unknown

Causes reversible?: Unknown

Extreme fluctuations?: Unknown

AOO (km2): Unknown

#### Locations

Number of locations: Unknown

Justification for number of locations: Data available (a single record) are not enough to estimate the number of locations.

Trend: Unknown

Extreme fluctuations?: Unknown

#### Population

Number of individuals: Unknown

Trend: Unknown

Causes ceased?: Unknown

Causes understood?: Unknown

Causes reversible?: Unknown

Extreme fluctuations?: Unknown

Population Information (Narrative): No estimates of population size exist.

#### Subpopulations

Number of subpopulations: Unknown

Trend: Unknown

Justification for trend: Data available (a single record) are not enough to estimate the number of subpopulations.

Extreme fluctuations?: Unknown

Severe fragmentation?: Unknown

#### Habitat

System: Terrestrial

Habitat specialist: Unknown

Habitat (narrative): This spider is known from only one site in a dune field.

Trend in extent, area or quality?: Unknown

##### Habitat

Habitat importance: Major Importance

Habitats: 13.3. Marine Coastal/Supratidal - Coastal Sand Dunes

#### Habitat

Habitat importance: Major Importance

Habitats: 13.3. Marine Coastal/Supratidal - Coastal Sand Dunes

#### Ecology

Size: 4.07 mm

Generation length (yr): 1

Dependency of single sp?: No

Ecology and traits (narrative): A nocturnal, tube-web builder that presumably feeds on a variety of small invertebrates.

#### Threats

Justification for threats: No known threats.

##### Threats

Threat type: Past

Threats: 12. Other options - Other threat

#### Threats

Threat type: Past

Threats: 12. Other options - Other threat

#### Conservation

Justification for conservation actions: The single known site for this spider is currently protected by the Natura 2000 network (PTCON0012 and PTZPE0015). Additionally, it is also covered by the Sudoeste Alentejano e Costa Vicentina Natural Park.

##### Conservation actions

Conservation action type: In Place

Conservation actions: 1.1. Land/water protection - Site/area protection1.2. Land/water protection - Resource & habitat protection

#### Conservation actions

Conservation action type: In Place

Conservation actions: 1.1. Land/water protection - Site/area protection1.2. Land/water protection - Resource & habitat protection

#### Other

##### Use and trade

Use type: International

##### Ecosystem services

Ecosystem service type: Very important

##### Research needed

Research needed: 1.2. Research - Population size, distribution & trends1.3. Research - Life history & ecology1.5. Research - Threats

Justification for research needed: Research is needed on basic information such as distribution, ecology, life cycle and possible threats throughout the species range.

#### Use and trade

Use type: International

#### Ecosystem services

Ecosystem service type: Very important

#### Research needed

Research needed: 1.2. Research - Population size, distribution & trends1.3. Research - Life history & ecology1.5. Research - Threats

Justification for research needed: Research is needed on basic information such as distribution, ecology, life cycle and possible threats throughout the species range.

#### Viability analysis

### Scotophaeus dolanskyi

#### Species information

Scientific name: Scotophaeus dolanskyi

Species authority: Lissner, 2017

Kingdom: Animalia

Phylum: Arthropoda

Class: Arachnida

Order: Araneae

Family: Gnaphosidae

Region for assessment: Global

#### Geographic range

Biogeographic realm: Palearctic

Countries: Portugal

Map of records (Google Earth): Suppl. material 12

Basis of EOO and AOO: Unknown

Basis (narrative): There is only one record for the species (Lissner 2017a) in a maquis in Sobral da Adiça, Beja. Its true range is therefore unknown and not possible to model with confidence.

Min Elevation/Depth (m): 350

Max Elevation/Depth (m): 350

Range description: This spider is known from only one site in a maquis in Sobral da Adica, Beja, southern Portugal (Lissner 2017a).

#### New occurrences

#### Extent of occurrence

EOO (km2): Unknown

Trend: Unknown

Causes ceased?: Unknown

Causes understood?: Unknown

Causes reversible?: Unknown

Extreme fluctuations?: Unknown

#### Area of occupancy

Trend: Unknown

Causes ceased?: Unknown

Causes understood?: Unknown

Causes reversible?: Unknown

Extreme fluctuations?: Unknown

AOO (km2): Unknown

#### Locations

Number of locations: Unknown

Justification for number of locations: The data available (a single record) are not enough to estimate the number of locations.

Trend: Unknown

Extreme fluctuations?: Unknown

#### Population

Number of individuals: Unknown

Trend: Unknown

Causes ceased?: Unknown

Causes understood?: Unknown

Causes reversible?: Unknown

Extreme fluctuations?: Unknown

Population Information (Narrative): No estimates of population size exist.

#### Subpopulations

Number of subpopulations: Unknown

Trend: Unknown

Justification for trend: The data available (a single record) are not enough to estimate the number of subpopulations.

Extreme fluctuations?: Unknown

Severe fragmentation?: Unknown

#### Habitat

System: Terrestrial

Habitat specialist: Unknown

Habitat (narrative): This spider is known from only one site in a maquis.

Trend in extent, area or quality?: Unknown

##### Habitat

Habitat importance: Major Importance

Habitats: 3.8. Shrubland - Mediterranean-type Shrubby Vegetation

#### Habitat

Habitat importance: Major Importance

Habitats: 3.8. Shrubland - Mediterranean-type Shrubby Vegetation

#### Ecology

Size: 5.9 mm

Generation length (yr): 1

Dependency of single sp?: No

Ecology and traits (narrative): If similar to congeners, a nocturnal active hunter that is found at the understorey level, eating a variety of small invertebrates.

#### Threats

Justification for threats: The existence of threats is unknown for this species.

##### Threats

Threat type: Past

Threats: 12. Other options - Other threat

#### Threats

Threat type: Past

Threats: 12. Other options - Other threat

#### Conservation

Justification for conservation actions: The single record for this spider is in the Natura 2000 network and its true range could be totally or at least partially covered by this instrument (PTZPE0045 and PTCON0053).

##### Conservation actions

Conservation action type: In Place

Conservation actions: 1.1. Land/water protection - Site/area protection1.2. Land/water protection - Resource & habitat protection

#### Conservation actions

Conservation action type: In Place

Conservation actions: 1.1. Land/water protection - Site/area protection1.2. Land/water protection - Resource & habitat protection

#### Other

##### Use and trade

Use type: International

##### Ecosystem services

Ecosystem service type: Very important

##### Research needed

Research needed: 1.2. Research - Population size, distribution & trends1.3. Research - Life history & ecology1.5. Research - Threats

Justification for research needed: Research is needed on basic information such as distribution, ecology, life cycle and possible threats throughout the range of the species.

#### Use and trade

Use type: International

#### Ecosystem services

Ecosystem service type: Very important

#### Research needed

Research needed: 1.2. Research - Population size, distribution & trends1.3. Research - Life history & ecology1.5. Research - Threats

Justification for research needed: Research is needed on basic information such as distribution, ecology, life cycle and possible threats throughout the range of the species.

#### Viability analysis

### Scotophaeus nanoides

#### Species information

Scientific name: Scotophaeus nanoides

Species authority: Wunderlich, 2011

Kingdom: Animalia

Phylum: Arthropoda

Class: Arachnida

Order: Araneae

Family: Gnaphosidae

Region for assessment: Global

#### Geographic range

Biogeographic realm: Palearctic

Countries: Portugal

Map of records (Google Earth): Suppl. material 13

Basis of EOO and AOO: Unknown

Basis (narrative): Largely unknown, as there are only two records for the species (Lecigne 2017, Wunderlich 2011) from the Algarve. Its true range is therefore unknown and not possible to model with confidence.

Min Elevation/Depth (m): 50

Max Elevation/Depth (m): 50

Range description: This spider is known from only two sites in southern Portugal, one is the small village of Olhos de Água and the other is an unspecified site somewhere near the city of São Brás de Alportel, both in the province of Faro, Algarve (Lecigne 2017, Wunderlich 2011).

#### New occurrences

#### Extent of occurrence

EOO (km2): Unknown

Trend: Unknown

Causes ceased?: Unknown

Causes understood?: Unknown

Causes reversible?: Unknown

Extreme fluctuations?: Unknown

#### Area of occupancy

Trend: Unknown

Causes ceased?: Unknown

Causes understood?: Unknown

Causes reversible?: Unknown

Extreme fluctuations?: Unknown

AOO (km2): Unknown

#### Locations

Number of locations: Unknown

Justification for number of locations: The data available (two records) are not enough to estimate the number of locations.

Trend: Unknown

Extreme fluctuations?: Unknown

#### Population

Number of individuals: Unknown

Trend: Unknown

Causes ceased?: Unknown

Causes understood?: Unknown

Causes reversible?: Unknown

Extreme fluctuations?: No

Population Information (Narrative): No estimates of population size exist.

#### Subpopulations

Number of subpopulations: Unknown

Trend: Unknown

Justification for trend: The data available (two records) are not enough to estimate the number of subpopulations.

Extreme fluctuations?: Unknown

Severe fragmentation?: Unknown

#### Habitat

System: Terrestrial

Habitat specialist: Unknown

Habitat (narrative): This spider is known from only two sites. The habitat of both places is unspecified.

Trend in extent, area or quality?: Unknown

##### Habitat

Habitat importance: Major Importance

Habitats: 18. Unknown

#### Habitat

Habitat importance: Major Importance

Habitats: 18. Unknown

#### Ecology

Size: 5 mm

Generation length (yr): 1

Dependency of single sp?: No

Ecology and traits (narrative): If similar to congeners, this spider is a nocturnal active hunter that lives at the understorey level.

#### Threats

Justification for threats: The existence of threats is unknown for this species.

##### Threats

Threat type: Past

Threats: 12. Other options - Other threat

#### Threats

Threat type: Past

Threats: 12. Other options - Other threat

#### Conservation

Justification for conservation actions: It is unknown exactly where this spider was found in São Brás de Alportel. Future records might reveal its true range to be partially covered by the Natura 2000 network (PTCON0049 and PTCON0057) as it is within 5 km of known occurence points.

##### Conservation actions

Conservation action type: In Place

Conservation actions: 1.1. Land/water protection - Site/area protection1.2. Land/water protection - Resource & habitat protection

#### Conservation actions

Conservation action type: In Place

Conservation actions: 1.1. Land/water protection - Site/area protection1.2. Land/water protection - Resource & habitat protection

#### Other

##### Use and trade

Use type: International

##### Ecosystem services

Ecosystem service type: Very important

##### Research needed

Research needed: 1.2. Research - Population size, distribution & trends1.3. Research - Life history & ecology1.5. Research - Threats

Justification for research needed: Research is needed on basic information such as distribution, ecology, life cycle and possible threats throughout the range.

#### Use and trade

Use type: International

#### Ecosystem services

Ecosystem service type: Very important

#### Research needed

Research needed: 1.2. Research - Population size, distribution & trends1.3. Research - Life history & ecology1.5. Research - Threats

Justification for research needed: Research is needed on basic information such as distribution, ecology, life cycle and possible threats throughout the range.

#### Viability analysis

### Trachyzelotes minutus

#### Species information

Scientific name: Trachyzelotes minutus

Species authority: Crespo, 2010

Kingdom: Animalia

Phylum: Arthropoda

Class: Arachnida

Order: Araneae

Family: Gnaphosidae

Region for assessment: Global

#### Geographic range

Biogeographic realm: Palearctic

Countries: Portugal

Map of records (Google Earth): Suppl. material 14

Basis of EOO and AOO: Unknown

Basis (narrative): There are only two records for the species (Crespo and Mendes 2010). The true range is therefore unknown and not possible to model with confidence.

Min Elevation/Depth (m): 200

Max Elevation/Depth (m): 230

Range description: This spider is known from only two sites in south Portugal, one in Corval and the other one in Montoito, Évora (Crespo and Mendes 2010). They are also both in cork oak woodlands with scattered bushes.

#### New occurrences

#### Extent of occurrence

EOO (km2): Unknown

Trend: Unknown

Causes ceased?: Unknown

Causes understood?: Unknown

Causes reversible?: Unknown

Extreme fluctuations?: Unknown

#### Area of occupancy

Trend: Unknown

Causes ceased?: Unknown

Causes understood?: Unknown

Causes reversible?: Unknown

Extreme fluctuations?: Unknown

AOO (km2): Unknown

#### Locations

Number of locations: Unknown

Justification for number of locations: The data available (two records) are not enough to estimate the number of locations.

Trend: Unknown

Extreme fluctuations?: Unknown

#### Population

Number of individuals: Unknown

Trend: Unknown

Causes ceased?: Unknown

Causes understood?: Unknown

Causes reversible?: Unknown

Extreme fluctuations?: No

Population Information (Narrative): No estimates of population size exist.

#### Subpopulations

Number of subpopulations: Unknown

Trend: Unknown

Justification for trend: The data available (two records) are not enough to estimate the number of subpopulations.

Extreme fluctuations?: Unknown

Severe fragmentation?: Unknown

#### Habitat

System: Terrestrial

Habitat specialist: Unknown

Habitat (narrative): This spider is known from only two sites, both cork oak woodlands with scattered bushes.

Trend in extent, area or quality?: Unknown

##### Habitat

Habitat importance: Major Importance

Habitats: 1.4. Forest - Temperate

#### Habitat

Habitat importance: Major Importance

Habitats: 1.4. Forest - Temperate

#### Ecology

Size: 2.36 - 2.97 mm

Generation length (yr): 1

Dependency of single sp?: No

Ecology and traits (narrative): If similar to congeners, these spiders are active predators that consume a variety of invertebrates at ground level.

#### Threats

Justification for threats: The existence of threats is unknown for this species.

##### Threats

Threat type: Past

Threats: 12. Other options - Other threat

#### Threats

Threat type: Past

Threats: 12. Other options - Other threat

#### Conservation

Justification for conservation actions: This spider has not been recorded in areas inside or adjacent to protected areas. More records are needed in order to confirm or disprove this for the species true range.

##### Conservation actions

Conservation action type: Needed

Conservation actions: 1.1. Land/water protection - Site/area protection1.2. Land/water protection - Resource & habitat protection

#### Conservation actions

Conservation action type: Needed

Conservation actions: 1.1. Land/water protection - Site/area protection1.2. Land/water protection - Resource & habitat protection

#### Other

##### Use and trade

Use type: International

##### Ecosystem services

Ecosystem service type: Very important

##### Research needed

Research needed: 1.2. Research - Population size, distribution & trends1.3. Research - Life history & ecology1.5. Research - Threats

Justification for research needed: Research is needed on basic information such as distribution, ecology, life cycle and possible threats throughout the range.

#### Use and trade

Use type: International

#### Ecosystem services

Ecosystem service type: Very important

#### Research needed

Research needed: 1.2. Research - Population size, distribution & trends1.3. Research - Life history & ecology1.5. Research - Threats

Justification for research needed: Research is needed on basic information such as distribution, ecology, life cycle and possible threats throughout the range.

#### Viability analysis

### Zelotes fuzeta

#### Species information

Scientific name: Zelotes fuzeta

Species authority: Wunderlich, 2011

Kingdom: Animalia

Phylum: Arthropoda

Class: Arachnida

Order: Araneae

Family: Gnaphosidae

Region for assessment: Global

#### Geographic range

Biogeographic realm: Palearctic

Countries: Portugal

Map of records (Google Earth): Suppl. material 15

Basis of EOO and AOO: Unknown

Basis (narrative): Largely unknown, as there is only one record for the species (Wunderlich 2011). The true range is therefore unknown and not possible to model with confidence.

Min Elevation/Depth (m): 10

Max Elevation/Depth (m): 10

Range description: This spider is known from only one site in a sandy area near the beach east of Fuseta, Algarve, southern Portugal (Wunderlich 2011).

#### New occurrences

#### Extent of occurrence

EOO (km2): Unknown

Trend: Unknown

Causes ceased?: Unknown

Causes understood?: Unknown

Causes reversible?: Unknown

Extreme fluctuations?: Unknown

#### Area of occupancy

Trend: Unknown

Causes ceased?: Unknown

Causes understood?: Unknown

Causes reversible?: Unknown

Extreme fluctuations?: Unknown

AOO (km2): Unknown

#### Locations

Number of locations: Unknown

Justification for number of locations: The data available (a single record) are not enough to estimate the number of locations.

Trend: Unknown

Extreme fluctuations?: No

#### Population

Number of individuals: Unknown

Trend: Unknown

Causes ceased?: Unknown

Causes understood?: Unknown

Causes reversible?: Unknown

Extreme fluctuations?: Unknown

Population Information (Narrative): No estimates of population size exist.

#### Subpopulations

Trend: Unknown

Justification for trend: The data available (a single record) are not enough to estimate the number of subpopulations.

Extreme fluctuations?: Unknown

Severe fragmentation?: Unknown

#### Habitat

System: Terrestrial

Habitat specialist: Unknown

Habitat (narrative): This spider is known from only one site in a sandy beach.

Trend in extent, area or quality?: Unknown

##### Habitat

Habitat importance: Major Importance

Habitats: 13.3. Marine Coastal/Supratidal - Coastal Sand Dunes

#### Habitat

Habitat importance: Major Importance

Habitats: 13.3. Marine Coastal/Supratidal - Coastal Sand Dunes

#### Ecology

Size: 2.8 mm

Generation length (yr): 1

Dependency of single sp?: No

Ecology and traits (narrative): If similar to conspecifics, this spider is an active ground hunter that does not build a web and consumes a variety of small invertebrates.

#### Threats

Justification for threats: The existence of threats is unknown for this species.

##### Threats

Threat type: Past

Threats: 12. Other options - Other threat

#### Threats

Threat type: Past

Threats: 12. Other options - Other threat

#### Conservation

Justification for conservation actions: It is unknown exactly where this spider was collected but it is fair to assume that its true range might be completely or at least partially covered by the Natura 2000 network (PTZPE0017 and PTCON0013). It might also be totally or partially covered by the Ria Formosa Natural Park.

##### Conservation actions

Conservation action type: In Place

Conservation actions: 1.1. Land/water protection - Site/area protection1.2. Land/water protection - Resource & habitat protection

#### Conservation actions

Conservation action type: In Place

Conservation actions: 1.1. Land/water protection - Site/area protection1.2. Land/water protection - Resource & habitat protection

#### Other

##### Use and trade

Use type: International

##### Ecosystem services

Ecosystem service type: Very important

##### Research needed

Research needed: 1.2. Research - Population size, distribution & trends1.3. Research - Life history & ecology1.5. Research - Threats

Justification for research needed: Research is needed on basic information such as distribution, ecology, life cycle and possible threats throughout the range.

#### Use and trade

Use type: International

#### Ecosystem services

Ecosystem service type: Very important

#### Research needed

Research needed: 1.2. Research - Population size, distribution & trends1.3. Research - Life history & ecology1.5. Research - Threats

Justification for research needed: Research is needed on basic information such as distribution, ecology, life cycle and possible threats throughout the range.

#### Viability analysis

### Macrothele calpeiana

#### Species information

Scientific name: Macrothele calpeiana

Species authority: Walckenaer, 1805

Common names: Mígala-dos-montados

Kingdom: Animalia

Phylum: Arthropoda

Class: Arachnida

Order: Araneae

Family: Macrothelidae

Taxonomic notes: *Macrothele calpeiana*'s records form four distinct groups. Recent work (Arnedo unpublished) suggests that the individuals recorded in Portugal may belong to a species separate from those found in Spain.

Region for assessment: Global

#### Geographic range

Biogeographic realm: Palearctic

Countries: PortugalSpain

Map of records (Google Earth): Suppl. material 16

Basis of EOO and AOO: Species Distribution Model

Basis (narrative): Multiple collection sites are recorded for this species (174 records) from references both recent and otherwise, but with remarkably little habitat information (González-Moliné 2018, Bellvert and Arnedo 2016, Pulido and del Pozo 2010, Cardoso 2008). It was possible to perform species distribution modelling to predict its potential range with confidence limits. See Methods for details.

Min Elevation/Depth (m): 0

Max Elevation/Depth (m): 2000

Range description: This spider is a widespread species present throughout the majority of southern Iberian Peninsula (González-Moliné 2018, Bellvert and Arnedo 2016, Pulido and del Pozo 2010, Cardoso 2008). It has also been recorded in northern Spain and in Illes Balears, but these are suspected introductions, along with further records in Italy, Netherlands, Belgium and Switzerland. Species distribution modelling predicts that it might be widespread through Andalusia and the Algarve.

#### New occurrences

#### Extent of occurrence

EOO (km2): 75926-97837

Trend: Stable

Causes ceased?: Yes

Causes understood?: Yes

Causes reversible?: Yes

Extreme fluctuations?: No

#### Area of occupancy

Trend: Stable

Causes ceased?: Yes

Causes understood?: Yes

Causes reversible?: Yes

Extreme fluctuations?: No

AOO (km2): 34532-52868

#### Locations

Number of locations: Not applicable

Justification for number of locations: No known threats to the species.

Trend: Stable

Extreme fluctuations?: Unknown

#### Population

Number of individuals: Unknown

Trend: Unknown

Causes ceased?: Unknown

Causes understood?: Unknown

Causes reversible?: Unknown

Extreme fluctuations?: Unknown

Population Information (Narrative): No estimates of population size exist.

#### Subpopulations

Trend: Unknown

Justification for trend: No known threats to the species.

Extreme fluctuations?: Unknown

Severe fragmentation?: Unknown

#### Habitat

System: Terrestrial

Habitat specialist: No

Habitat (narrative): This spider is known primarily from mediterranean forests dominated by *Quercus suber* and was originally considered a bioindicator for this habitat. However, subsequent collections revealed that it could be found in a variety of habitats, including anthropic habitats such as pine and olive plantations and even road-sides, old walls and rubbish dumps (Bellvert and Arnedo 2016, Helsdingen and Decae 1992).

Trend in extent, area or quality?: Unknown

##### Habitat

Habitat importance: Major Importance

Habitats: 1.4. Forest - Temperate

##### Habitat

Habitat importance: Suitable

Habitats: 14.3. Artificial/Terrestrial - Plantations14.5. Artificial/Terrestrial - Urban Areas

#### Habitat

Habitat importance: Major Importance

Habitats: 1.4. Forest - Temperate

#### Habitat

Habitat importance: Suitable

Habitats: 14.3. Artificial/Terrestrial - Plantations14.5. Artificial/Terrestrial - Urban Areas

#### Ecology

Size: 30-60 mm

Generation length (yr): 1

Dependency of single sp?: No

Ecology and traits (narrative): This tube- and sheet-web builder creates small burrows on vertical walls, tree trunks or even at ground level, often adopting existing burrows. It is predominantly nocturnal and is reported as performing both sit-and-wait hunting, as well as active hunting.

#### Threats

Justification for threats: The existence of threats is unknown for this species.

##### Threats

Threat type: Past

Threats: 12. Other options - Other threat

#### Threats

Threat type: Past

Threats: 12. Other options - Other threat

#### Conservation

Justification for conservation actions: This spider is found in a variety of protected areas, namely the Fonte Benémola Local Protected Landscape and the Rocha da Pena Local Protected Landscape in Portugal and the Doñana National Park, La Breña y Marismas del Barbate Natural Park, the Sierra de Huétor Natural Park, Los Alcornocales Natural Park (and others) in Spain. Additionally, it is also present in a large variety of areas covered by the Natura 2000 network.

##### Conservation actions

Conservation action type: In Place

Conservation actions: 1.1. Land/water protection - Site/area protection1.2. Land/water protection - Resource & habitat protection

#### Conservation actions

Conservation action type: In Place

Conservation actions: 1.1. Land/water protection - Site/area protection1.2. Land/water protection - Resource & habitat protection

#### Other

##### Use and trade

Use type: International

##### Ecosystem services

Ecosystem service type: Very important

##### Research needed

Research needed: 3.1. Monitoring - Population trends3.4. Monitoring - Habitat trends

Justification for research needed: Monitoring of population and habitat are important to confirm inferred trends.

#### Use and trade

Use type: International

#### Ecosystem services

Ecosystem service type: Very important

#### Research needed

Research needed: 3.1. Monitoring - Population trends3.4. Monitoring - Habitat trends

Justification for research needed: Monitoring of population and habitat are important to confirm inferred trends.

#### Viability analysis

### Leptoneta berlandi

#### Species information

Scientific name: Leptoneta berlandi

Species authority: Machado & Ribera, 1986

Kingdom: Animalia

Phylum: Arthropoda

Class: Arachnida

Order: Araneae

Family: Leptonetidae

Region for assessment: Global

#### Geographic range

Biogeographic realm: Palearctic

Countries: Portugal

Map of records (Google Earth): Suppl. material 17

Basis of EOO and AOO: Unknown

Basis (narrative): There are only two old records for the species. Its true range is therefore unknown and not possible to model with confidence.

Min Elevation/Depth (m): 50

Max Elevation/Depth (m): 50

Range description: This spider is known from only two sites of unspecified habitat in the province of Porto, one from Monte Pedral and another from Entre-os-Rios (Machado 1937, Morano et al. 2019).

#### New occurrences

#### Extent of occurrence

EOO (km2): Unknown

Trend: Unknown

Causes ceased?: Unknown

Causes understood?: Unknown

Causes reversible?: Unknown

Extreme fluctuations?: Unknown

#### Area of occupancy

Trend: Unknown

Causes ceased?: Unknown

Causes understood?: Unknown

Causes reversible?: Unknown

Extreme fluctuations?: Unknown

AOO (km2): Unknown

#### Locations

Number of locations: Unknown

Justification for number of locations: The data available (two records) are not enough to estimate the number of locations.

Trend: Unknown

Extreme fluctuations?: Unknown

#### Population

Number of individuals: Unknown

Trend: Unknown

Causes ceased?: Unknown

Causes understood?: Unknown

Causes reversible?: Unknown

Extreme fluctuations?: No

Population Information (Narrative): No estimates of population size exist.

#### Subpopulations

Number of subpopulations: Unknown

Trend: Unknown

Justification for trend: The data available (two records) are not enough to estimate the number of subpopulations.

Extreme fluctuations?: Unknown

Severe fragmentation?: Unknown

#### Habitat

System: Terrestrial

Habitat specialist: No

Habitat (narrative): This spider is known from only two sites of unspecified habitat.

Trend in extent, area or quality?: Unknown

##### Habitat

Habitat importance: Suitable

Habitats: 18. Unknown

#### Habitat

Habitat importance: Suitable

Habitats: 18. Unknown

#### Ecology

Size: 1.92 - 2.08 mm

Generation length (yr): 1

Dependency of single sp?: No

Ecology and traits (narrative): If similar to congeners, a nocturnal ground-dwelling species that hides under rocks and forest litter and captures prey through the use of a space web.

#### Threats

Justification for threats: The existence of threats is unknown for this species.

##### Threats

Threat type: Past

Threats: 12. Other options - Other threat

#### Threats

Threat type: Past

Threats: 12. Other options - Other threat

#### Conservation

Justification for conservation actions: This spider has not been recorded in areas inside or adjacent to protected areas. More records are needed in order to confirm or disprove this for the species' true range.

##### Conservation actions

Conservation action type: Needed

Conservation actions: 1.1. Land/water protection - Site/area protection1.2. Land/water protection - Resource & habitat protection

#### Conservation actions

Conservation action type: Needed

Conservation actions: 1.1. Land/water protection - Site/area protection1.2. Land/water protection - Resource & habitat protection

#### Other

##### Use and trade

Use type: International

##### Ecosystem services

Ecosystem service type: Very important

##### Research needed

Research needed: 1.2. Research - Population size, distribution & trends1.3. Research - Life history & ecology1.5. Research - Threats

Justification for research needed: Research is needed on basic information such as distribution, ecology, life cycle and possible threats throughout the range.

#### Use and trade

Use type: International

#### Ecosystem services

Ecosystem service type: Very important

#### Research needed

Research needed: 1.2. Research - Population size, distribution & trends1.3. Research - Life history & ecology1.5. Research - Threats

Justification for research needed: Research is needed on basic information such as distribution, ecology, life cycle and possible threats throughout the range.

#### Viability analysis

### Leptoneta conimbricensis

#### Species information

Scientific name: Leptoneta conimbricensis

Species authority: Machado & Ribera, 1986

Kingdom: Animalia

Phylum: Arthropoda

Class: Arachnida

Order: Araneae

Family: Leptonetidae

Region for assessment: Global

#### Geographic range

Biogeographic realm: Palearctic

Countries: Portugal

Map of records (Google Earth): Suppl. material 18

Basis of EOO and AOO: Unknown

Basis (narrative): Largely unknown, as there are only four old records for the species. Its true range is therefore unknown and not possible to model with confidence.

Min Elevation/Depth (m): 50

Max Elevation/Depth (m): 400

Range description: This spider is known from only four sites in central Portugal, two of them in caves in Coimbra, two more in sites of unspecified habitat in Aveiro (Machado 1941, Machado 1945, Machado and Ribera 1986).

#### New occurrences

#### Extent of occurrence

EOO (km2): Unknown

Trend: Unknown

Justification for trend: There are no currently known threats to the species.

Causes ceased?: Unknown

Causes understood?: Unknown

Causes reversible?: Unknown

Extreme fluctuations?: No

#### Area of occupancy

Trend: Unknown

Justification for trend: There are no currently known threats to the species.

Causes ceased?: Unknown

Causes understood?: Unknown

Causes reversible?: Unknown

Extreme fluctuations?: No

AOO (km2): Unknown

#### Locations

Number of locations: Unknown

Justification for number of locations: Data available (4 records) are not enough to estimate the number of locations.

Trend: Unknown

Extreme fluctuations?: Unknown

#### Population

Number of individuals: Unknown

Trend: Unknown

Justification for trend: There are no currently known threats to the species.

Causes ceased?: Unknown

Causes understood?: Unknown

Causes reversible?: Unknown

Extreme fluctuations?: No

Population Information (Narrative): No estimates of population size exist.

#### Subpopulations

Number of subpopulations: Unknown

Trend: Unknown

Justification for trend: Data available (4 records) are not enough to estimate the number of subpopulations.

Extreme fluctuations?: Unknown

Severe fragmentation?: Unknown

#### Habitat

System: Terrestrial

Habitat specialist: Unknown

Habitat (narrative): There are only four records for the species, two of them in caves in Coimbra, two more in sites of unspecified habitat in Aveiro.

Trend in extent, area or quality?: Unknown

##### Habitat

Habitat importance: Major Importance

Habitats: 7.1. Caves and Subterranean Habitats (non-aquatic) - Caves

#### Habitat

Habitat importance: Major Importance

Habitats: 7.1. Caves and Subterranean Habitats (non-aquatic) - Caves

#### Ecology

Size: 1.79 - 2.01 mm

Generation length (yr): 1

Dependency of single sp?: No

Ecology and traits (narrative): Troglophile. A nocturnal ground-dwelling species that hides under rocks and forest litter and captures prey through the use of a space web.

#### Threats

Justification for threats: The existence of threats is unknown for this species.

##### Threats

Threat type: Past

Threats: 12. Other options - Other threat

#### Threats

Threat type: Past

Threats: 12. Other options - Other threat

#### Conservation

Justification for conservation actions: This spider has not been recorded in areas inside or adjacent to protected areas. More records are needed in order to confirm or disprove this for the species' true range.

##### Conservation actions

Conservation action type: Needed

Conservation actions: 1.1. Land/water protection - Site/area protection1.2. Land/water protection - Resource & habitat protection

#### Conservation actions

Conservation action type: Needed

Conservation actions: 1.1. Land/water protection - Site/area protection1.2. Land/water protection - Resource & habitat protection

#### Other

##### Use and trade

Use type: International

##### Ecosystem services

Ecosystem service type: Very important

##### Research needed

Research needed: 1.2. Research - Population size, distribution & trends1.3. Research - Life history & ecology1.5. Research - Threats

Justification for research needed: Research is needed on basic information such as distribution, ecology, life cycle and possible threats throughout the range.

#### Use and trade

Use type: International

#### Ecosystem services

Ecosystem service type: Very important

#### Research needed

Research needed: 1.2. Research - Population size, distribution & trends1.3. Research - Life history & ecology1.5. Research - Threats

Justification for research needed: Research is needed on basic information such as distribution, ecology, life cycle and possible threats throughout the range.

#### Viability analysis

### Teloleptoneta synthetica

#### Species information

Scientific name: Teloleptoneta synthetica

Species authority: Machado, 1951

Kingdom: Animalia

Phylum: Arthropoda

Class: Arachnida

Order: Araneae

Family: Leptonetidae

Region for assessment: Global

#### Geographic range

Biogeographic realm: Palearctic

Countries: Portugal

Map of records (Google Earth): Suppl. material 19

Basis of EOO and AOO: Observed

Basis (narrative): Multiple collection sites are recorded for this species (8 records), mostly recent and exclusively in caves (Machado 1945, Reboleira 2012). Due to its nature as a troglobiont species and the fact that Portuguese caves are relatively well sampled, the known record points should accurately reflect reality.

Min Elevation/Depth (m): 10

Max Elevation/Depth (m): 500

Range description: This spider has been recorded in three areas, the Algarve and two mountain ranges, Serra da Adiça in Alentejo and Serra da Arrábida in Setúbal (Machado 1945, Reboleira 2012).

#### New occurrences

#### Extent of occurrence

EOO (km2): 22681

Trend: Stable

Justification for trend: There are no currently known threats to the species.

Causes ceased?: Yes

Causes understood?: Yes

Causes reversible?: Yes

Extreme fluctuations?: No

#### Area of occupancy

Trend: Stable

Justification for trend: There are no currently known threats to the species.

Causes ceased?: Yes

Causes understood?: Yes

Causes reversible?: Yes

Extreme fluctuations?: No

AOO (km2): 32

#### Locations

Number of locations: Not applicable

Justification for number of locations: There are no currently known threats to the species.

Trend: Stable

Extreme fluctuations?: Unknown

#### Population

Number of individuals: Unknown

Trend: Stable

Justification for trend: There are no currently known threats to the species.

Causes ceased?: Yes

Causes understood?: Yes

Causes reversible?: Yes

Extreme fluctuations?: No

Population Information (Narrative): No estimates of population size exist.

#### Subpopulations

Number of subpopulations: Unknown

Trend: Stable

Justification for trend: There are no currently known threats to the species.

Extreme fluctuations?: No

Severe fragmentation?: Unknown

#### Habitat

System: Terrestrial

Habitat specialist: Yes

Habitat (narrative): This spider is a troglobiont species of highly restricted habitat found strictly within caves.

Trend in extent, area or quality?: Unknown

##### Habitat

Habitat importance: Major Importance

Habitats: 7.1. Caves and Subterranean Habitats (non-aquatic) - Caves

#### Habitat

Habitat importance: Major Importance

Habitats: 7.1. Caves and Subterranean Habitats (non-aquatic) - Caves

#### Ecology

Size: 1.98 - 2.36 mm

Generation length (yr): 1

Dependency of single sp?: No

Ecology and traits (narrative): Troglobiont species with eyes very reduced in size, sometimes absent. Pigmentation is completely absent. It builds flat webs often under rocks on the cave floor where it can be found sitting waiting for prey.

#### Threats

Justification for threats: The existence of threats is unknown for this species.

##### Threats

Threat type: Past

Threats: 12. Other options - Other threat

#### Threats

Threat type: Past

Threats: 12. Other options - Other threat

#### Conservation

Justification for conservation actions: One of the areas that this spider inhabits is both a protected area, the Arrábida Natural Park, as well as an area covered by the Natura 2000 network (PTCON0010).

##### Conservation actions

Conservation action type: In Place

Conservation actions: 1.1. Land/water protection - Site/area protection1.2. Land/water protection - Resource & habitat protection

#### Conservation actions

Conservation action type: In Place

Conservation actions: 1.1. Land/water protection - Site/area protection1.2. Land/water protection - Resource & habitat protection

#### Other

##### Use and trade

Use type: International

##### Ecosystem services

Ecosystem service type: Very important

##### Research needed

Research needed: 3.1. Monitoring - Population trends3.4. Monitoring - Habitat trends

Justification for research needed: Monitoring of population and habitat are important to confirm inferred trends.

#### Use and trade

Use type: International

#### Ecosystem services

Ecosystem service type: Very important

#### Research needed

Research needed: 3.1. Monitoring - Population trends3.4. Monitoring - Habitat trends

Justification for research needed: Monitoring of population and habitat are important to confirm inferred trends.

#### Viability analysis

### Bordea berlandi

#### Species information

Scientific name: Bordea berlandi

Species authority: Fage, 1931

Kingdom: Animalia

Phylum: Arthropoda

Class: Arachnida

Order: Araneae

Family: Linyphiidae

Region for assessment: Global

#### Geographic range

Biogeographic realm: Palearctic

Countries: Portugal

Map of records (Google Earth): Suppl. material 20

Basis of EOO and AOO: Species Distribution Model

Basis (narrative): Multiple collection sites are recorded for this species (30 records), mostly recent and in caves, albeit rarely it can also be found in pinewood forests (Fage 1931, Machado 1942, Cardoso 2004, Bosmans et al. 2010). It was possible to perform species distribution modelling to predict its potential range with confidence limits. See Methods for details.

Min Elevation/Depth (m): 0

Max Elevation/Depth (m): 894

Range description: This spider is a well-established presence in cave systems throughout north and central Portugal, with a single record existing for Monchique in the Algarve (Fage 1931, Machado 1942, Cardoso 2004, Bosmans et al. 2010). The species distribution modelling confirms its widespread nature, as well as predicting that the species could also occur in Spain's north-westernmost region of Galicia.

#### New occurrences

#### Extent of occurrence

EOO (km2): 53080 - 70571

Trend: Decline (inferred)

Justification for trend: Many of the underground habitats that this spider inhabits have been damaged or destroyed by pollution from human and agricultural activity (such as septic tanks and other forms of waste), infrastructure building (such as wind farms), quarries and overall disturbance from human presence, including the destruction and removal of geological structures that form this species' habitat.

Causes ceased?: No

Causes understood?: Yes

Causes reversible?: No

Extreme fluctuations?: No

#### Area of occupancy

Trend: Decline (inferred)

Justification for trend: Many of the underground habitats that this spider inhabits have been damaged or destroyed by pollution from human and agricultural activity (such as septic tanks and other forms of waste), infrastructure building (such as wind farms), quarries and overall disturbance from human presence, including the destruction and removal of geological structures that form this species' habitat.

Causes ceased?: No

Causes understood?: Yes

Causes reversible?: No

Extreme fluctuations?: No

AOO (km2): 19184 - 36776

#### Locations

Number of locations: Unknown

Justification for number of locations: The number of threats needed to completely cover the species range is unknown but, in any case, larger than 10.

Trend: Decline (inferred)

Justification for trend: Many of the underground habitats that this spider inhabits have been damaged or destroyed by pollution from human and agricultural activity (such as septic tanks and other forms of waste), infrastructure building (such as wind farms), quarries and overall disturbance from human presence, including the destruction and removal of geological structures that form this species' habitat.

Extreme fluctuations?: Unknown

#### Population

Number of individuals: Unknown

Trend: Decline (inferred)

Justification for trend: Many of the underground habitats that this spider inhabits have been damaged or destroyed by pollution from human and agricultural activity (such as septic tanks and other forms of waste), infrastructure building (such as wind farms), quarries and overall disturbance from human presence, including the destruction and removal of geological structures that form this species' habitat.

Basis for decline: (c) a decline in area of occupancy, extent of occurrence and/or quality of habitat

Causes ceased?: No

Causes understood?: Yes

Causes reversible?: No

Extreme fluctuations?: No

Population Information (Narrative): No estimates of population size exist.

#### Subpopulations

Number of subpopulations: Unknown

Trend: Decline (inferred)

Justification for trend: Many of the underground habitats that this spider inhabits have been damaged or destroyed by pollution from human and agricultural activity (such as septic tanks and other forms of waste), infrastructure building (such as wind farms), quarries and overall disturbance from human presence, including the destruction and removal of geological structures that form this species' habitat.

Extreme fluctuations?: No

Severe fragmentation?: No

#### Habitat

System: Terrestrial

Habitat specialist: No

Habitat (narrative): This spider is a troglophile species recorded mostly in caves (18). A fair amount of records possess no habitat information (9) and, less commonly, they possess information for pinewood forests (2) and a mine (1).

Trend in extent, area or quality?: Decline (inferred)

Justification for trend: Many of the underground habitats that this spider inhabits have been damaged or destroyed by pollution from human and agricultural activity (such as septic tanks and other forms of waste), infrastructure building (such as wind farms), quarries and overall disturbance from human presence, including the destruction and removal of geological structures that form this species' habitat.

##### Habitat

Habitat importance: Major Importance

Habitats: 1.4. Forest - Temperate7.1. Caves and Subterranean Habitats (non-aquatic) - Caves7.2. Caves and Subterranean Habitats (non-aquatic) - Other Subterranean Habitats

#### Habitat

Habitat importance: Major Importance

Habitats: 1.4. Forest - Temperate7.1. Caves and Subterranean Habitats (non-aquatic) - Caves7.2. Caves and Subterranean Habitats (non-aquatic) - Other Subterranean Habitats

#### Ecology

Size: 1.7 - 2.1 mm

Generation length (yr): 1

Dependency of single sp?: No

Ecology and traits (narrative): Troglophile species. A nocturnal ground-dwelling species that eats a variety of small invertebrates, capturing its prey through the use of a sheet web.

#### Threats

Justification for threats: Many of the underground habitats that this spider inhabits have been damaged or destroyed by pollution from human and agricultural activity (such as septic tanks and other forms of waste), infrastructure building (such as wind farms), quarries and overall disturbance from human presence, including the destruction and removal of geological structures that form this species' habitat.

##### Threats

Threat type: Ongoing

Threats: 2.1. Agriculture & aquaculture - Annual & perennial non-timber crops3.2. Energy production & mining - Mining & quarrying3.3. Energy production & mining - Renewable energy

#### Threats

Threat type: Ongoing

Threats: 2.1. Agriculture & aquaculture - Annual & perennial non-timber crops3.2. Energy production & mining - Mining & quarrying3.3. Energy production & mining - Renewable energy

#### Conservation

Justification for conservation actions: This spider is a widespread species. Some of the caves it inhabits are currently covered by protected areas namely the Serra de Aire e Candeeiros Natural Park, the Peneda-Gerês National Park and the Montejunto Regional Protected Landscape. It is therefore reasonable to be expected that it is covered at large by a variety of sites designated by the Natura 2000 network.

##### Conservation actions

Conservation action type: In Place

Conservation actions: 1.1. Land/water protection - Site/area protection1.2. Land/water protection - Resource & habitat protection

#### Conservation actions

Conservation action type: In Place

Conservation actions: 1.1. Land/water protection - Site/area protection1.2. Land/water protection - Resource & habitat protection

#### Other

##### Use and trade

Use type: International

##### Ecosystem services

Ecosystem service type: Very important

##### Research needed

Research needed: 3.1. Monitoring - Population trends3.4. Monitoring - Habitat trends

Justification for research needed: Monitoring of population and habitat are important to confirm inferred trends.

#### Use and trade

Use type: International

#### Ecosystem services

Ecosystem service type: Very important

#### Research needed

Research needed: 3.1. Monitoring - Population trends3.4. Monitoring - Habitat trends

Justification for research needed: Monitoring of population and habitat are important to confirm inferred trends.

#### Viability analysis

### Labulla machadoi

#### Species information

Scientific name: Labulla machadoi

Species authority: Hormiga & Scharff, 2005

Kingdom: Animalia

Phylum: Arthropoda

Class: Arachnida

Order: Araneae

Family: Linyphiidae

Region for assessment: Global

#### Geographic range

Biogeographic realm: Palearctic

Countries: Portugal

Map of records (Google Earth): Suppl. material 21

Basis of EOO and AOO: Species Distribution Model

Basis (narrative): Multiple collection sites are recorded for this species (5 records). It was possible to perform species distribution modelling to predict its potential range with confidence limits. See Methods for details.

Min Elevation/Depth (m): 11

Max Elevation/Depth (m): 754

Range description: This spider has been recorded only in northern Portugal, mostly recent but without much habitat information although at least one record was from a mixed oak forest (Hormiga and Scharff 2005, Cardoso et al. 2008b). The species distribution modelling predicts that its distribution could be scarce, restricted to small patches in the north-westernmost tip of Portugal.

#### New occurrences

#### Extent of occurrence

EOO (km2): 2742 - 3415

Trend: Stable

Justification for trend: There are no currently known threats to the species.

Causes ceased?: Yes

Causes understood?: Yes

Causes reversible?: Yes

Extreme fluctuations?: No

#### Area of occupancy

Trend: Stable

Justification for trend: There are no currently known threats to the species.

Causes ceased?: Yes

Causes understood?: Yes

Causes reversible?: Yes

Extreme fluctuations?: No

AOO (km2): 908 - 1512

#### Locations

Number of locations: Not applicable

Justification for number of locations: There are no currently known threats to the species.

Trend: Stable

Extreme fluctuations?: Unknown

#### Population

Number of individuals: Unknown

Trend: Stable

Justification for trend: There are no currently known threats to the species.

Causes ceased?: Yes

Causes understood?: Yes

Causes reversible?: Yes

Extreme fluctuations?: No

Population Information (Narrative): No estimates of population size exist.

#### Subpopulations

Number of subpopulations: Unknown

Trend: Stable

Justification for trend: There are no currently known threats to the species.

Extreme fluctuations?: No

Severe fragmentation?: No

#### Habitat

System: Terrestrial

Habitat specialist: Unknown

Habitat (narrative): Despite being recorded several times, only one of this spider's records includes habitat information for a mixed oak woodland containing *Quercus robur* and *Quercus pyrenaica* (Cardoso et al. 2008b).

Trend in extent, area or quality?: Unknown

Justification for trend: There are no currently known threats to the species.

##### Habitat

Habitat importance: Major Importance

Habitats: 1.4. Forest - Temperate

#### Habitat

Habitat importance: Major Importance

Habitats: 1.4. Forest - Temperate

#### Ecology

Size: 4.2 - 5.6 mm

Generation length (yr): 1

Dependency of single sp?: No

Ecology and traits (narrative): A nocturnal species that eats a variety of small invertebrates through the use of a sheet web in tree trunks, large branches and more secluded places.

#### Threats

Justification for threats: The existence of threats is unknown for this species.

##### Threats

Threat type: Past

Threats: 12. Other options - Other threat

#### Threats

Threat type: Past

Threats: 12. Other options - Other threat

#### Conservation

Justification for conservation actions: This spider has been recorded once inside the Peneda-Gerês National Park and another time in Paredes de Coura, close (~1.5 km) to the Corno do Bico Regional Protected Landscape. Both of these areas are covered by the Natura 2000 network (PTCON0001; PTCON0040; PTZPE0002).

##### Conservation actions

Conservation action type: In Place

Conservation actions: 1.1. Land/water protection - Site/area protection1.2. Land/water protection - Resource & habitat protection

#### Conservation actions

Conservation action type: In Place

Conservation actions: 1.1. Land/water protection - Site/area protection1.2. Land/water protection - Resource & habitat protection

#### Other

##### Use and trade

Use type: International

##### Ecosystem services

Ecosystem service type: Very important

##### Research needed

Research needed: 3.1. Monitoring - Population trends3.4. Monitoring - Habitat trends

Justification for research needed: Monitoring of population and habitat are important to confirm inferred trends.

#### Use and trade

Use type: International

#### Ecosystem services

Ecosystem service type: Very important

#### Research needed

Research needed: 3.1. Monitoring - Population trends3.4. Monitoring - Habitat trends

Justification for research needed: Monitoring of population and habitat are important to confirm inferred trends.

#### Viability analysis

### Maso douro

#### Species information

Scientific name: Maso douro

Species authority: Bosmans & Cardoso, 2010

Kingdom: Animalia

Phylum: Arthropoda

Class: Arachnida

Order: Araneae

Family: Linyphiidae

Region for assessment: Global

#### Geographic range

Biogeographic realm: Palearctic

Countries: Portugal

Map of records (Google Earth): Suppl. material 22

Basis of EOO and AOO: Unknown

Basis (narrative): Largely unknown as there is only one record for the species (Bosmans et al. 2010) in Douro Internacional Natural Park, Bragança.

Min Elevation/Depth (m): 690

Max Elevation/Depth (m): 690

Range description: This spider is known from only one site in a *Quercus pyrenaica* (Willd.) forest (Bosmans et al. 2010). The species' true range is therefore unknown and not possible to model with confidence.

#### New occurrences

#### Extent of occurrence

EOO (km2): Unknown

Trend: Unknown

Causes ceased?: Unknown

Causes understood?: Unknown

Causes reversible?: Unknown

Extreme fluctuations?: Unknown

#### Area of occupancy

Trend: Unknown

Causes ceased?: Unknown

Causes understood?: Unknown

Causes reversible?: Unknown

Extreme fluctuations?: Unknown

AOO (km2): Unknown

#### Locations

Number of locations: Unknown

Justification for number of locations: Data available (1 record) are not enough to estimate the number of locations.

Trend: Unknown

Extreme fluctuations?: Unknown

#### Population

Number of individuals: Unknown

Trend: Unknown

Causes ceased?: Unknown

Causes understood?: Unknown

Causes reversible?: Unknown

Extreme fluctuations?: No

Population Information (Narrative): No estimates of population size exist.

#### Subpopulations

Number of subpopulations: Unknown

Trend: Unknown

Justification for trend: Data available (1 record) are not enough to estimate the number of locations.

Extreme fluctuations?: Unknown

Severe fragmentation?: Unknown

#### Habitat

System: Terrestrial

Habitat specialist: Unknown

Habitat (narrative): This spider is known from only one site in a *Quercus pyrenaica* forest on a slope over a small river.

Trend in extent, area or quality?: Unknown

##### Habitat

Habitat importance: Major Importance

Habitats: 1.4. Forest - Temperate

#### Habitat

Habitat importance: Major Importance

Habitats: 1.4. Forest - Temperate

#### Ecology

Size: 1.1 mm

Generation length (yr): 1

Dependency of single sp?: No

Ecology and traits (narrative): A ground-dwelling species that is active during both night and day, actively hunting for a variety of small invertebrates.

#### Threats

Justification for threats: The existence of threats is unknown for this species.

##### Threats

Threat type: Past

Threats: 12. Other options - Other threat

#### Threats

Threat type: Past

Threats: 12. Other options - Other threat

#### Conservation

Justification for conservation actions: The species true range might be completely or at least partially covered by the Douro Internacional Natural Park.

##### Conservation actions

Conservation action type: In Place

Conservation actions: 1.1. Land/water protection - Site/area protection1.2. Land/water protection - Resource & habitat protection

#### Conservation actions

Conservation action type: In Place

Conservation actions: 1.1. Land/water protection - Site/area protection1.2. Land/water protection - Resource & habitat protection

#### Other

##### Use and trade

Use type: International

##### Ecosystem services

Ecosystem service type: Very important

##### Research needed

Research needed: 1.2. Research - Population size, distribution & trends1.3. Research - Life history & ecology1.5. Research - Threats

Justification for research needed: Research is needed on basic information such as distribution, ecology, life cycle and possible threats throughout the range (if any).

#### Use and trade

Use type: International

#### Ecosystem services

Ecosystem service type: Very important

#### Research needed

Research needed: 1.2. Research - Population size, distribution & trends1.3. Research - Life history & ecology1.5. Research - Threats

Justification for research needed: Research is needed on basic information such as distribution, ecology, life cycle and possible threats throughout the range (if any).

#### Viability analysis

### Parapelecopsis conimbricensis

#### Species information

Scientific name: Parapelecopsis conimbricensis

Species authority: Bosmans & Crespo, 2010

Kingdom: Animalia

Phylum: Arthropoda

Class: Arachnida

Order: Araneae

Family: Linyphiidae

Region for assessment: Global

#### Geographic range

Biogeographic realm: Palearctic

Countries: Portugal

Map of records (Google Earth): Suppl. material 23

Basis of EOO and AOO: None

Basis (narrative): Largely unknown, as there are only three records for the species (Bosmans et al. 2010, Crespo et al. 2010) in sites from the provinces of Portalegre and Coimbra.

Min Elevation/Depth (m): 10

Max Elevation/Depth (m): 50

Range description: This spider is known from only three sites (Bosmans et al. 2010, Crespo et al. 2010): one in Portalegre in an area of unspecified habitat, one in a marsh and the other in a botanical garden next to a river and a spring, these last two found both in Coimbra. The true range is therefore unknown and not possible to model with confidence.

#### New occurrences

#### Extent of occurrence

EOO (km2): Unknown

Trend: Unknown

Causes ceased?: Unknown

Causes understood?: Unknown

Causes reversible?: Unknown

Extreme fluctuations?: Unknown

#### Area of occupancy

Trend: Unknown

Causes ceased?: Unknown

Causes understood?: Unknown

Causes reversible?: Unknown

Extreme fluctuations?: Unknown

AOO (km2): Unknown

#### Locations

Number of locations: Unknown

Justification for number of locations: Data available (3 records) are not enough to estimate the number of locations.

Trend: Unknown

Extreme fluctuations?: Unknown

#### Population

Number of individuals: Unknown

Trend: Unknown

Causes ceased?: Unknown

Causes understood?: Unknown

Causes reversible?: Unknown

Extreme fluctuations?: Unknown

Population Information (Narrative): No estimates of population size exist.

#### Subpopulations

Number of subpopulations: Unknown

Trend: Unknown

Extreme fluctuations?: Unknown

Severe fragmentation?: Unknown

#### Habitat

System: Terrestrial

Habitat specialist: Unknown

Habitat (narrative): This spider is known from only three sites, one in a marsh and the other in a botanical garden next to a river and a spring.

Trend in extent, area or quality?: Unknown

##### Habitat

Habitat importance: Major Importance

Habitats: 5.4. Wetlands (inland) - Bogs, Marshes, Swamps, Fens, Peatlands

##### Habitat

Habitat importance: Suitable

Habitats: 14.5. Artificial/Terrestrial - Urban Areas

#### Habitat

Habitat importance: Major Importance

Habitats: 5.4. Wetlands (inland) - Bogs, Marshes, Swamps, Fens, Peatlands

#### Habitat

Habitat importance: Suitable

Habitats: 14.5. Artificial/Terrestrial - Urban Areas

#### Ecology

Size: 1.7 - 1.8 mm

Generation length (yr): 1

Dependency of single sp?: No

Ecology and traits (narrative): A ground-dwelling species that produces no web and hunts actively, consuming a variety of small invertebrates.

#### Threats

Justification for threats: The existence of threats is unknown for this species.

##### Threats

Threat type: Past

Threats: 12. Other options - Other threat

#### Threats

Threat type: Past

Threats: 12. Other options - Other threat

#### Conservation

Justification for conservation actions: This spider was collected in three sites, one of which was in the centre of the Paul de Arzila Natural Park, which in turn is located inside the Natura 2000 network (PTZPE0005, PTCON0005).

##### Conservation actions

Conservation action type: In Place

Conservation actions: 1.1. Land/water protection - Site/area protection1.2. Land/water protection - Resource & habitat protection

#### Conservation actions

Conservation action type: In Place

Conservation actions: 1.1. Land/water protection - Site/area protection1.2. Land/water protection - Resource & habitat protection

#### Other

##### Use and trade

Use type: International

##### Ecosystem services

Ecosystem service type: Very important

##### Research needed

Research needed: 1.2. Research - Population size, distribution & trends1.3. Research - Life history & ecology1.5. Research - Threats

Justification for research needed: Research is needed on basic information such as distribution, ecology, life cycle and possible threats throughout the range (if any).

#### Use and trade

Use type: International

#### Ecosystem services

Ecosystem service type: Very important

#### Research needed

Research needed: 1.2. Research - Population size, distribution & trends1.3. Research - Life history & ecology1.5. Research - Threats

Justification for research needed: Research is needed on basic information such as distribution, ecology, life cycle and possible threats throughout the range (if any).

#### Viability analysis

### Trichoncus similipes

#### Species information

Scientific name: Trichoncus similipes

Species authority: Denis, 1965

Kingdom: Animalia

Phylum: Arthropoda

Class: Arachnida

Order: Araneae

Family: Linyphiidae

Region for assessment: Global

#### Geographic range

Biogeographic realm: Palearctic

Countries: Portugal

Map of records (Google Earth): Suppl. material 24

Basis of EOO and AOO: Unknown

Basis (narrative): Largely unknown, as there are only two records for the species (Cardoso et al. 2008b, Denis 1965) in northern Portugal, in the provinces of Braga and Porto.

Min Elevation/Depth (m): 100

Max Elevation/Depth (m): 660

Range description: This spider is known from only two sites in Braga and Porto, north-western Portugal (Cardoso et al. 2008b, Denis 1965). Its true range is therefore unknown and not possible to model with confidence.

#### New occurrences

#### Extent of occurrence

EOO (km2): Unknown

Trend: Unknown

Causes ceased?: Unknown

Causes understood?: Unknown

Causes reversible?: Unknown

Extreme fluctuations?: Unknown

#### Area of occupancy

Trend: Unknown

Causes ceased?: Unknown

Causes understood?: Unknown

Causes reversible?: Unknown

Extreme fluctuations?: Unknown

AOO (km2): Unknown

#### Locations

Number of locations: Unknown

Justification for number of locations: Data available (2 records) are not enough to estimate the number of locations.

Trend: Unknown

Extreme fluctuations?: Unknown

#### Population

Number of individuals: Unknown

Trend: Unknown

Causes ceased?: Unknown

Causes understood?: Unknown

Causes reversible?: Unknown

Extreme fluctuations?: No

Population Information (Narrative): No estimates of population size exist.

#### Subpopulations

Number of subpopulations: Unknown

Trend: Unknown

Justification for trend: Data available (2 record) are not enough to estimate the number of subpopulations.

Extreme fluctuations?: Unknown

Severe fragmentation?: Unknown

#### Habitat

System: Terrestrial

Habitat specialist: Unknown

Habitat (narrative): One of the records (Cardoso et al. 2008b) is from Mata da Albergaria in a mixed oak woodland (*Quercus robur*, *Quercus pyrenaica*, amongst other native vegetation).

Trend in extent, area or quality?: Unknown

##### Habitat

Habitat importance: Major Importance

Habitats: 1.4. Forest - Temperate

#### Habitat

Habitat importance: Major Importance

Habitats: 1.4. Forest - Temperate

#### Ecology

Size: 1.7 - 2 mm

Generation length (yr): 1

Dependency of single sp?: No

Ecology and traits (narrative): An active hunter that builds no web and eats a variety of small invertebrates.

#### Threats

Justification for threats: The existence of threats is unknown for this species.

##### Threats

Threat type: Past

Threats: 12. Other options - Other threat

#### Threats

Threat type: Past

Threats: 12. Other options - Other threat

#### Conservation

Justification for conservation actions: This spider was collected in two sites, one of which, Mata da Albergaria, is located in the Peneda-Gerêz National Park and covered by the Natura 2000 network (PTZPE0002, very close to PTCON0024).

##### Conservation actions

Conservation action type: In Place

Conservation actions: 1.1. Land/water protection - Site/area protection1.2. Land/water protection - Resource & habitat protection

#### Conservation actions

Conservation action type: In Place

Conservation actions: 1.1. Land/water protection - Site/area protection1.2. Land/water protection - Resource & habitat protection

#### Other

##### Use and trade

Use type: International

##### Ecosystem services

Ecosystem service type: Very important

##### Research needed

Research needed: 1.2. Research - Population size, distribution & trends1.3. Research - Life history & ecology1.5. Research - Threats

Justification for research needed: Research is needed on basic information such as distribution, ecology, life cycle and possible threats throughout the range (if any).

#### Use and trade

Use type: International

#### Ecosystem services

Ecosystem service type: Very important

#### Research needed

Research needed: 1.2. Research - Population size, distribution & trends1.3. Research - Life history & ecology1.5. Research - Threats

Justification for research needed: Research is needed on basic information such as distribution, ecology, life cycle and possible threats throughout the range (if any).

#### Viability analysis

### Apostenus crespoi

#### Species information

Scientific name: Apostenus crespoi

Species authority: Lissner, 2017

Kingdom: Animalia

Phylum: Arthropoda

Class: Arachnida

Order: Araneae

Family: Liocranidae

Region for assessment: Global

#### Geographic range

Biogeographic realm: Palearctic

Countries: Portugal

Map of records (Google Earth): Suppl. material 25

Basis of EOO and AOO: Unknown

Basis (narrative): Largely unknown, as there is only one record for the species (Lissner 2017a), in a maquis near Azeitão, Setúbal.

Min Elevation/Depth (m): 195

Max Elevation/Depth (m): 195

Range description: This spider is known from only one site in a maquis in a limestone region (Lissner 2017a). Its true range is therefore unknown and not possible to model with confidence.

#### New occurrences

#### Extent of occurrence

EOO (km2): Unknown

Trend: Unknown

Causes ceased?: Unknown

Causes understood?: Unknown

Causes reversible?: Unknown

Extreme fluctuations?: Unknown

#### Area of occupancy

Trend: Unknown

Causes ceased?: Unknown

Causes understood?: Unknown

Causes reversible?: Unknown

Extreme fluctuations?: Unknown

AOO (km2): Unknown

#### Locations

Number of locations: Unknown

Justification for number of locations: Data available (1 record) are not enough to estimate the number of locations.

Trend: Unknown

Extreme fluctuations?: Unknown

#### Population

Number of individuals: Unknown

Trend: Unknown

Causes ceased?: Unknown

Causes understood?: Unknown

Causes reversible?: Unknown

Extreme fluctuations?: Unknown

Population Information (Narrative): No estimates of population size exist.

#### Subpopulations

Number of subpopulations: Unknown

Trend: Unknown

Justification for trend: Data available (1 record) are not enough to estimate the number of subpopulations.

Extreme fluctuations?: Unknown

Severe fragmentation?: Unknown

#### Habitat

System: Terrestrial

Habitat specialist: Unknown

Habitat (narrative): This spider is known from only one site in a maquis in a limestone region.

Trend in extent, area or quality?: Unknown

##### Habitat

Habitat importance: Major Importance

Habitats: 3.8. Shrubland - Mediterranean-type Shrubby Vegetation

#### Habitat

Habitat importance: Major Importance

Habitats: 3.8. Shrubland - Mediterranean-type Shrubby Vegetation

#### Ecology

Size: 3.32 mm

Generation length (yr): 1

Dependency of single sp?: No

Ecology and traits (narrative): A ground-level active hunter that constructs no web and hides under stones and litter during the day.

#### Threats

Justification for threats: The existence of threats is unknown for this species.

##### Threats

Threat type: Past

Threats: 12. Other options - Other threat

#### Threats

Threat type: Past

Threats: 12. Other options - Other threat

#### Conservation

Justification for conservation actions: Although the species' true range is unknown, its single record is currently inside the Arrábida Natural Park. Additionally, this spider's record is located in an area covered by the Natura 2000 network (PTCON0010).

##### Conservation actions

Conservation action type: In Place

Conservation actions: 1.1. Land/water protection - Site/area protection1.2. Land/water protection - Resource & habitat protection

#### Conservation actions

Conservation action type: In Place

Conservation actions: 1.1. Land/water protection - Site/area protection1.2. Land/water protection - Resource & habitat protection

#### Other

##### Use and trade

Use type: International

##### Ecosystem services

Ecosystem service type: Very important

##### Research needed

Research needed: 1.2. Research - Population size, distribution & trends1.3. Research - Life history & ecology1.5. Research - Threats

Justification for research needed: Research is needed on basic information such as distribution, ecology, life cycle and possible threats throughout the range.

#### Use and trade

Use type: International

#### Ecosystem services

Ecosystem service type: Very important

#### Research needed

Research needed: 1.2. Research - Population size, distribution & trends1.3. Research - Life history & ecology1.5. Research - Threats

Justification for research needed: Research is needed on basic information such as distribution, ecology, life cycle and possible threats throughout the range.

#### Viability analysis

### Nemesia bacelarae

#### Species information

Scientific name: Nemesia bacelarae

Species authority: Decae, Cardoso & Selden, 2007

Common names: Buraqueira-de-bacelar

Kingdom: Animalia

Phylum: Arthropoda

Class: Arachnida

Order: Araneae

Family: Nemesiidae

Region for assessment: Global

#### Geographic range

Biogeographic realm: Palearctic

Countries: Portugal

Map of records (Google Earth): Suppl. material 26

Basis of EOO and AOO: Species Distribution Model

Basis (narrative): Multiple collection sites are recorded for this species (17 records) mostly recent and in a variety of different habitats (Cardoso 2004, Decae et al. 2007, Tavares et al. 2007, Crespo et al. 2009). It was possible to perform species distribution modelling to predict its potential range with confidence limits. See Methods for details.

Min Elevation/Depth (m): 0

Max Elevation/Depth (m): 1656

Range description: This spider has been frequently recorded in northern and central Portugal (Cardoso 2004, Decae et al. 2007, Tavares et al. 2007, Crespo et al. 2009). The species distribution modelling predicts that this species could be widespread throughout north and central Portugal, both coastland and mainland. It could also be present in north-western Spain.

#### New occurrences

#### Extent of occurrence

EOO (km2): 56871 - 69882

Trend: Stable

Justification for trend: Despite the present threats to some of its subpopulations, the wide geographical and habitat range of this spider makes it plausible that the trend is mostly stable.

Causes ceased?: Yes

Causes understood?: Yes

Causes reversible?: Yes

Extreme fluctuations?: No

#### Area of occupancy

Trend: Stable

Justification for trend: Despite the present threats to some of its subpopulations, the wide geographical and habitat range of this spider makes it plausible that the trend is mostly stable.

Causes ceased?: Yes

Causes understood?: Yes

Causes reversible?: Yes

Extreme fluctuations?: No

AOO (km2): 26468 - 48928

#### Locations

Number of locations: Unknown

Justification for number of locations: The number of threats needed to completely cover the species range is unknown but, in any case, larger than 10.

Trend: Stable

Extreme fluctuations?: Unknown

#### Population

Number of individuals: Unknown

Trend: Stable

Justification for trend: Despite the present threats to some of its subpopulations, the wide geographical and habitat range of this spider makes it plausible that the trend is mostly stable.

Causes ceased?: Yes

Causes understood?: Yes

Causes reversible?: Yes

Extreme fluctuations?: No

Population Information (Narrative): No estimates of population size exist.

#### Subpopulations

Number of subpopulations: Unknown

Trend: Stable

Justification for trend: Despite the present threats to some of its subpopulations, the wide geographical and habitat range of this spider makes it plausible that the trend is mostly stable.

Extreme fluctuations?: No

Severe fragmentation?: No

#### Habitat

System: Terrestrial

Habitat specialist: No

Habitat (narrative): Highly diverse. This spider has been found in marshes, grasslands, shrublands (*Cytisus* and *Genista*), forests (*Quercus* spp.) and plantations (*Eucalyptus* sp. and *Pinus* spp.), most often in clay or compacted soil.

Trend in extent, area or quality?: Stable

##### Habitat

Habitat importance: Major Importance

Habitats: 1.4. Forest - Temperate3.8. Shrubland - Mediterranean-type Shrubby Vegetation4.4. Grassland - Temperate

##### Habitat

Habitat importance: Suitable

Habitats: 16. Introduced vegetation

#### Habitat

Habitat importance: Major Importance

Habitats: 1.4. Forest - Temperate3.8. Shrubland - Mediterranean-type Shrubby Vegetation4.4. Grassland - Temperate

#### Habitat

Habitat importance: Suitable

Habitats: 16. Introduced vegetation

#### Ecology

Size: 11.9 - 17.2 mm

Generation length (yr): 1

Dependency of single sp?: No

Ecology and traits (narrative): Males appear to be nocturnal. A ground-dwelling species that builds vertical burrows with a trapdoor entry and eats a variety of small invertebrates (mainly beetles).

#### Threats

Justification for threats: Several subpopulations in central Portugal (mostly in the Coimbra region) are threatened by urban development and have either been eradicated or severely depleted. Unsurpassable obstacles and lack of safe corridors present a challenge to male dispersal in urban and peri-urban populations, which have often been found dead or dying trying to overcome human infrastructures (volunteers have attempted to reduce this by rescuing males and returning them to the vicinities of known colonies).

##### Threats

Threat type: Ongoing

Threats: 1.1. Residential & commercial development - Housing & urban areas1.3. Residential & commercial development - Tourism & recreation areas

#### Threats

Threat type: Ongoing

Threats: 1.1. Residential & commercial development - Housing & urban areas1.3. Residential & commercial development - Tourism & recreation areas

#### Conservation

Justification for conservation actions: This spider has been recorded in several protected areas across its distribution, from its south-westernmost tip at the Arrábida Natural Park to its north-easternmost tip at the Douro International Natural Park. Due to its widespread nature, it is no doubt present in numerous areas protected by the Natura 2000 network.

##### Conservation actions

Conservation action type: In Place

Conservation actions: 1.1. Land/water protection - Site/area protection1.2. Land/water protection - Resource & habitat protection

#### Conservation actions

Conservation action type: In Place

Conservation actions: 1.1. Land/water protection - Site/area protection1.2. Land/water protection - Resource & habitat protection

#### Other

##### Use and trade

Use type: International

##### Ecosystem services

Ecosystem service type: Very important

##### Research needed

Research needed: 3.1. Monitoring - Population trends3.4. Monitoring - Habitat trends

Justification for research needed: Monitoring of population and habitat are important to confirm inferred trends.

#### Use and trade

Use type: International

#### Ecosystem services

Ecosystem service type: Very important

#### Research needed

Research needed: 3.1. Monitoring - Population trends3.4. Monitoring - Habitat trends

Justification for research needed: Monitoring of population and habitat are important to confirm inferred trends.

#### Viability analysis

### Nemesia berlandi

#### Species information

Scientific name: Nemesia berlandi

Species authority: Frade & Bacelar, 1931

Kingdom: Animalia

Phylum: Arthropoda

Class: Arachnida

Order: Araneae

Family: Nemesiidae

Region for assessment: Global

#### Geographic range

Biogeographic realm: Palearctic

Countries: Portugal

Map of records (Google Earth): Suppl. material 27

Basis of EOO and AOO: Unknown

Basis (narrative): There is only one old published record for the species (Frade and Bacelar 1931) attributed to the small village of Fagilde, Viseu and two other records for typical burrows with moults found in the vicinity of the type locality.

Min Elevation/Depth (m): 400

Max Elevation/Depth (m): 400

Range description: This spider is known from only one site (Frade and Bacelar 1931) and the area immediately adjacent to the small village of Fagilde, Central Portugal. The true range is therefore unknown and not possible to model with confidence.

#### New occurrences

#### Extent of occurrence

EOO (km2): Unknown

Trend: Decline (inferred)

Justification for trend: Few records and a lack of population data are not enough to estimate the species range or extinction risk trend. However, habitat loss in the area due to agriculture, urban infrastructure development and wildfires indicates the species is likely declining.

Causes ceased?: No

Causes understood?: Yes

Causes reversible?: Yes

Extreme fluctuations?: No

#### Area of occupancy

Trend: Decline (inferred)

Justification for trend: Few records and a lack of population data are not enough to estimate the species range or extinction risk trend. However, habitat loss in the area due to agriculture, urban infrastructure development and wildfires indicates the species is likely declining.

Causes ceased?: No

Causes understood?: Yes

Causes reversible?: Yes

Extreme fluctuations?: No

AOO (km2): Unknown

#### Locations

Number of locations: Unknown

Justification for number of locations: Until further information on this spider's distribution is recorded, the number of locations is unknown.

Trend: Decline (inferred)

Justification for trend: Habitat loss in the area due to agriculture, urban infrastructure development and wildfires indicates the species is likely declining.

Extreme fluctuations?: Unknown

#### Population

Number of individuals: Unknown

Trend: Decline (inferred)

Justification for trend: Few records and a lack of populational data are not enough to estimate the species extinction risk. However, habitat loss in the area due to agriculture, urban infrastructure development and wildfires indicates the species is likely declining.

Basis for decline: (c) a decline in area of occupancy, extent of occurrence and/or quality of habitat

Causes ceased?: No

Causes understood?: Yes

Causes reversible?: Yes

Extreme fluctuations?: Unknown

Population Information (Narrative): No estimates of population size exist.

#### Subpopulations

Number of subpopulations: Unknown

Trend: Decline (inferred)

Justification for trend: The data available are not enough to estimate the number of subpopulations. However, habitat loss in the area due to agriculture, urban infrastructure development and wildfires indicates the species is likely declining.

Extreme fluctuations?: No

Severe fragmentation?: Unknown

#### Habitat

System: Terrestrial

Habitat specialist: No

Habitat (narrative): This spider was originally found in an unspecified habitat, but horizontal burrows (a distinct burrow not present in other Iberian species) were found in leaf litter on the verge of local forests.

Trend in extent, area or quality?: Decline (inferred)

Justification for trend: Habitat loss in the area due to agriculture, urban infrastructure development and wildfires indicates the species habitat is likely declining in area and quality.

##### Habitat

Habitat importance: Major Importance

Habitats: 1.4. Forest - Temperate

#### Habitat

Habitat importance: Major Importance

Habitats: 1.4. Forest - Temperate

#### Ecology

Size: 22 mm

Generation length (yr): 1

Dependency of single sp?: No

Ecology and traits (narrative): A ground-dwelling species that builds vertical burrows with a trapdoor entry and eats a variety of small invertebrates (mainly beetles).

#### Threats

Justification for threats: Habitat loss in the area due to agriculture, urban infrastructure development and wildfires indicates the species is likely declining.

##### Threats

Threat type: Ongoing

Threats: 1.1. Residential & commercial development - Housing & urban areas1.3. Residential & commercial development - Tourism & recreation areas2.1. Agriculture & aquaculture - Annual & perennial non-timber crops7.1.1. Natural system modifications - Fire & fire suppression - Increase in fire frequency/intensity

#### Threats

Threat type: Ongoing

Threats: 1.1. Residential & commercial development - Housing & urban areas1.3. Residential & commercial development - Tourism & recreation areas2.1. Agriculture & aquaculture - Annual & perennial non-timber crops7.1.1. Natural system modifications - Fire & fire suppression - Increase in fire frequency/intensity

#### Conservation

Justification for conservation actions: This spider has not been recorded in areas inside or adjacent to protected areas. More records are needed in order to confirm or disprove this for the species' true range.

##### Conservation actions

Conservation action type: Needed

Conservation actions: 1.1. Land/water protection - Site/area protection1.2. Land/water protection - Resource & habitat protection

#### Conservation actions

Conservation action type: Needed

Conservation actions: 1.1. Land/water protection - Site/area protection1.2. Land/water protection - Resource & habitat protection

#### Other

##### Use and trade

Use type: International

##### Ecosystem services

Ecosystem service type: Very important

##### Research needed

Research needed: 1.2. Research - Population size, distribution & trends1.3. Research - Life history & ecology1.5. Research - Threats

Justification for research needed: Research is needed on basic information such as distribution, ecology, life cycle and possible threats throughout the range.

#### Use and trade

Use type: International

#### Ecosystem services

Ecosystem service type: Very important

#### Research needed

Research needed: 1.2. Research - Population size, distribution & trends1.3. Research - Life history & ecology1.5. Research - Threats

Justification for research needed: Research is needed on basic information such as distribution, ecology, life cycle and possible threats throughout the range.

#### Viability analysis

### Nemesia fagei

#### Species information

Scientific name: Nemesia fagei

Species authority: Frade & Bacelar, 1931

Common names: Buraqueira-de-fage

Kingdom: Animalia

Phylum: Arthropoda

Class: Arachnida

Order: Araneae

Family: Nemesiidae

Region for assessment: Global

#### Geographic range

Biogeographic realm: Palearctic

Countries: Portugal

Map of records (Google Earth): Suppl. material 28

Basis of EOO and AOO: Species Distribution Model

Basis (narrative): Multiple collection sites are recorded for this species (12 records), mostly recent (Bacelar 1937, Main 1949, Decae et al. 2007). It was possible to perform species distribution modelling to predict its potential range with confidence limits. See Methods for details.

Min Elevation/Depth (m): 0

Max Elevation/Depth (m): 532

Range description: This spider has been recorded almost exclusively in the Algarve, being recorded only once in the nearby region of southern Alentejo (Bacelar 1937, Main 1949, Decae et al. 2007). The species distribution modelling suggests that it might also be present in south-western Spain, between the border with Portugal and the Guadalquivir’s delta – a similar prediction to that present in Decae et al. 2007.

#### New occurrences

#### Extent of occurrence

EOO (km2): 17013 - 20967

Trend: Decline (inferred)

Justification for trend: Several subpopulations have been eradicated by urban development and many more are often disturbed or depleted by trampling, mostly in touristic areas, to access the coast line. Severe wildfires have recently devastated known occurrence regions, affecting a considerable number of subpopulations. A few subpopulations have been eradicated in the past due to habitat destruction caused by eucalyptus plantations, but this threat appears to have been halted in recent years.

Causes ceased?: No

Causes understood?: Yes

Causes reversible?: No

Extreme fluctuations?: No

#### Area of occupancy

Trend: Decline (inferred)

Justification for trend: Several subpopulations have been eradicated by urban development and many more are often disturbed or depleted by trampling, mostly in touristic areas, to access the coast line. Severe wildfires have recently devastated known occurrence regions, affecting a considerable number of subpopulations. A few subpopulations have been eradicated in the past due to habitat destruction caused by eucalyptus plantations, but this threat appears to have been halted in recent years.

Causes ceased?: No

Causes understood?: Yes

Causes reversible?: No

Extreme fluctuations?: No

AOO (km2): 5028 - 7520

#### Locations

Number of locations: Unknown

Justification for number of locations: The number of threats needed to completely cover the species range is unknown but, in any case, larger than 10.

Trend: Decline (inferred)

Extreme fluctuations?: No

#### Population

Number of individuals: Unknown

Trend: Decline (inferred)

Justification for trend: Several subpopulations have been erradicated by urban development and many more are often disturbed or depleted by trampling, mostly in touristic areas, to access the coast line. Severe wildfires have recently devastated known occurrence regions, affecting a considerable number of subpopulations. A few subpopulations have been eradicated in the past due to habitat destruction caused by eucalyptus plantations, but this threat appears to have been halted in recent years.

Basis for decline: (c) a decline in area of occupancy, extent of occurrence and/or quality of habitat

Causes ceased?: No

Causes understood?: Yes

Causes reversible?: No

Extreme fluctuations?: No

Population Information (Narrative): No estimates of population size exist.

#### Subpopulations

Number of subpopulations: Unknown

Trend: Decline (inferred)

Justification for trend: Several subpopulations have been eradicated by urban development and many more are often disturbed or depleted by trampling, mostly in touristic areas, to access the coast line. Severe wildfires have recently devastated known occurrence regions, affecting a considerable number of subpopulations. A few subpopulations have been eradicated in the past due to habitat destruction caused by eucalyptus plantations, but this threat appears to have been halted in recent years.

Extreme fluctuations?: No

Severe fragmentation?: Unknown

#### Habitat

System: Terrestrial

Habitat specialist: No

Habitat (narrative): Habitat information was recorded only twice in bushlands (dominated by *Cystus* sp.). Expert knowledge suggests that it is found in coastal areas (open areas, plateaus, small cliffs and roadsides), as well as mountainous regions of oak forest (*Quercus suber*) in the Algarve.

Trend in extent, area or quality?: Decline (inferred)

Justification for trend: Several subpopulations have been eradicated by urban development and many more are often disturbed or depleted by trampling, mostly in touristic areas, to access the coast line. Severe wildfires have recently devastated known occurrence regions, affecting a considerable number of subpopulations. A few subpopulations have been eradicated in the past due to habitat destruction caused by eucalyptus plantations, but this threat appears to have been halted in recent years.

##### Habitat

Habitat importance: Major Importance

Habitats: 3.8. Shrubland - Mediterranean-type Shrubby Vegetation6. Rocky areas (e.g. inland cliffs, mountain peaks)13.1. Marine Coastal/Supratidal - Sea Cliffs and Rocky Offshore Islands

#### Habitat

Habitat importance: Major Importance

Habitats: 3.8. Shrubland - Mediterranean-type Shrubby Vegetation6. Rocky areas (e.g. inland cliffs, mountain peaks)13.1. Marine Coastal/Supratidal - Sea Cliffs and Rocky Offshore Islands

#### Ecology

Size: 7 - 17.2 mm

Generation length (yr): 1

Dependency of single sp?: No

Ecology and traits (narrative): A ground-dwelling, nocturnal spider that builds a tube web and eats a variety of small invertebrates (mainly beetles).

#### Threats

Justification for threats: Several subpopulations have been eradicated by urban development and many more are often disturbed or depleted by trampling, mostly in touristic areas, to access the coast line. Severe wildfires have recently devastated known occurrence regions, affecting a considerable number of subpopulations. A few subpopulations have been eradicated in the past due to habitat destruction caused by eucalyptus plantations, but this threat appears to have been halted in recent years.

##### Threats

Threat type: Ongoing

Threats: 1.1. Residential & commercial development - Housing & urban areas1.3. Residential & commercial development - Tourism & recreation areas7.1.1. Natural system modifications - Fire & fire suppression - Increase in fire frequency/intensity

##### Threats

Threat type: Past

Threats: 2.2.1. Agriculture & aquaculture - Wood & pulp plantations - Small-holder plantations2.2.2. Agriculture & aquaculture - Wood & pulp plantations - Agro-industry plantations

#### Threats

Threat type: Ongoing

Threats: 1.1. Residential & commercial development - Housing & urban areas1.3. Residential & commercial development - Tourism & recreation areas7.1.1. Natural system modifications - Fire & fire suppression - Increase in fire frequency/intensity

#### Threats

Threat type: Past

Threats: 2.2.1. Agriculture & aquaculture - Wood & pulp plantations - Small-holder plantations2.2.2. Agriculture & aquaculture - Wood & pulp plantations - Agro-industry plantations

#### Conservation

Justification for conservation actions: This spider has been recorded inside the Sudoeste Alentejano e Costa Vicentina Natural Park, as well as close to the Sapal de Castro Marim e Vila Real de Santo António Natural Reserve, where it is predicted to occur.

##### Conservation actions

Conservation action type: In Place

Conservation actions: 1.1. Land/water protection - Site/area protection1.2. Land/water protection - Resource & habitat protection

#### Conservation actions

Conservation action type: In Place

Conservation actions: 1.1. Land/water protection - Site/area protection1.2. Land/water protection - Resource & habitat protection

#### Other

##### Use and trade

Use type: International

##### Ecosystem services

Ecosystem service type: Very important

##### Research needed

Research needed: 1.5. Research - Threats3.1. Monitoring - Population trends3.4. Monitoring - Habitat trends

Justification for research needed: Monitoring of population and habitat are important to confirm inferred trends.

#### Use and trade

Use type: International

#### Ecosystem services

Ecosystem service type: Very important

#### Research needed

Research needed: 1.5. Research - Threats3.1. Monitoring - Population trends3.4. Monitoring - Habitat trends

Justification for research needed: Monitoring of population and habitat are important to confirm inferred trends.

#### Viability analysis

### Domitius lusitanicus

#### Species information

Scientific name: Domitius lusitanicus

Species authority: Fage, 1931

Kingdom: Animalia

Phylum: Arthropoda

Class: Arachnida

Order: Araneae

Family: Nesticiidae

Region for assessment: Global

#### Geographic range

Biogeographic realm: Palearctic

Countries: Portugal

Map of records (Google Earth): Suppl. material 29

Basis of EOO and AOO: Observed

Basis (narrative): Multiple collection sites are recorded for this species (22 records), mostly recent and all of them in caves (Fage 1931, Ribera 1988, Ribera and López-Pancorbo 2011, Reboleira 2012). Due to its nature as a troglobiont species and the fact that Portuguese caves are relatively well sampled, the known record points should accurately reflect reality.

Min Elevation/Depth (m): 100

Max Elevation/Depth (m): 440

Range description: This spider has been recorded in central Portugal, exclusively inhabiting caves belonging to the Maciço Calcário Estremenho (MCE), one of the largest limestone areas of the country.

#### New occurrences

#### Extent of occurrence

EOO (km2): 199936

Trend: Decline (inferred)

Justification for trend: Local limestone quarries cover large areas and may be reducing available habitat.

Causes ceased?: No

Causes understood?: Yes

Causes reversible?: No

Extreme fluctuations?: No

#### Area of occupancy

Trend: Decline (inferred)

Justification for trend: Local limestone quarries cover large areas and may be reducing available habitat.

Causes ceased?: No

Causes understood?: Yes

Causes reversible?: No

Extreme fluctuations?: No

AOO (km2): 88

#### Locations

Number of locations: Unknown

Justification for number of locations: The number of threats needed to completely cover the species range is unknown but, in any case, larger than 10.

Trend: Decline (inferred)

Justification for trend: Local limestone quarries cover large areas and may be reducing available habitat.

Extreme fluctuations?: Unknown

#### Population

Number of individuals: Unknown

Trend: Decline (inferred)

Justification for trend: Local limestone quarries cover large areas and may be reducing available habitat.

Basis for decline: (c) a decline in area of occupancy, extent of occurrence and/or quality of habitat

Causes ceased?: No

Causes understood?: Yes

Causes reversible?: No

Extreme fluctuations?: No

Population Information (Narrative): No estimates of population size exist.

#### Subpopulations

Number of subpopulations: Unknown

Trend: Decline (inferred)

Justification for trend: Local limestone quarries cover large areas and may be reducing available habitat.

Extreme fluctuations?: No

Severe fragmentation?: No

#### Habitat

System: Terrestrial

Habitat specialist: Yes

Habitat (narrative): This spider is a troglobiont species of highly restricted habitat, found exclusively in caves of the Maciço Calcário Estremenho (MCE).

Trend in extent, area or quality?: Unknown

##### Habitat

Habitat importance: Major Importance

Habitats: 7.1. Caves and Subterranean Habitats (non-aquatic) - Caves

#### Habitat

Habitat importance: Major Importance

Habitats: 7.1. Caves and Subterranean Habitats (non-aquatic) - Caves

#### Ecology

Size: 2.9 - 3.5 mm

Generation length (yr): 1

Dependency of single sp?: No

Ecology and traits (narrative): This spider belongs to a recently described genus that seems to be constituted entirely by troglobiont species. Its eyes are absent and it captures small invertebrates with a space web positioned on cave walls and often inside crevices.

#### Threats

Justification for threats: Local limestone quarries cover large areas and may be reducing available habitat.

##### Threats

Threat type: Ongoing

Threats: 3.2. Energy production & mining - Mining & quarrying

#### Threats

Threat type: Ongoing

Threats: 3.2. Energy production & mining - Mining & quarrying

#### Conservation

Justification for conservation actions: The majority of the caves, in which this spider occurs, are currently located inside the Serra de Aire e Candeeiros Natural Park, as well as the Natura 2000 network (PTCON0015).

##### Conservation actions

Conservation action type: In Place

Conservation actions: 1.1. Land/water protection - Site/area protection1.2. Land/water protection - Resource & habitat protection

#### Conservation actions

Conservation action type: In Place

Conservation actions: 1.1. Land/water protection - Site/area protection1.2. Land/water protection - Resource & habitat protection

#### Other

##### Use and trade

Use type: International

##### Ecosystem services

Ecosystem service type: Very important

##### Research needed

Research needed: 3.1. Monitoring - Population trends3.4. Monitoring - Habitat trends

Justification for research needed: Monitoring of population and habitat are important to confirm inferred trends.

#### Use and trade

Use type: International

#### Ecosystem services

Ecosystem service type: Very important

#### Research needed

Research needed: 3.1. Monitoring - Population trends3.4. Monitoring - Habitat trends

Justification for research needed: Monitoring of population and habitat are important to confirm inferred trends.

#### Viability analysis

### Pseudomogrus algarvensis

#### Species information

Scientific name: Pseudomogrus algarvensis

Species authority: Logunov & Marusik, 2003

Kingdom: Animalia

Phylum: Arthropoda

Class: Arachnida

Order: Araneae

Family: Salticidae

Region for assessment: Global

#### Geographic range

Biogeographic realm: Palearctic

Countries: Portugal

Map of records (Google Earth): Suppl. material 30

Basis of EOO and AOO: Unknown

Basis (narrative): There is only one record for the species (Logunov and Marusik 2003), attributed to Monte Gordo, Faro.

Min Elevation/Depth (m): 0

Max Elevation/Depth (m): 0

Range description: This spider is known from only one site in Monte Gordo, Algarve, Southern Portugal (Logunov and Marusik 2003). The true range is therefore unknown and not possible to model with confidence.

#### New occurrences

#### Extent of occurrence

EOO (km2): Unknown

Trend: Unknown

Causes ceased?: Unknown

Causes understood?: Unknown

Causes reversible?: Unknown

Extreme fluctuations?: Unknown

#### Area of occupancy

Trend: Unknown

Causes ceased?: Unknown

Causes understood?: Unknown

Causes reversible?: Unknown

Extreme fluctuations?: Unknown

AOO (km2): Unknown

#### Locations

Number of locations: Unknown

Justification for number of locations: Data available (1 record) are not enough to estimate the number of locations.

Trend: Unknown

Extreme fluctuations?: Unknown

#### Population

Number of individuals: Unknown

Trend: Unknown

Causes ceased?: Unknown

Causes understood?: Unknown

Causes reversible?: Unknown

Extreme fluctuations?: Unknown

Population Information (Narrative): No estimates of population size exist.

#### Subpopulations

Number of subpopulations: Unknown

Trend: Unknown

Justification for trend: Data available (1 record) are not enough to estimate the number of subpopulations.

Extreme fluctuations?: Unknown

Severe fragmentation?: Unknown

#### Habitat

System: Terrestrial

Habitat specialist: Unknown

Habitat (narrative): This spider is known from only one site in dunes and it is impossible to know if it is exclusive to it, using available data.

Trend in extent, area or quality?: Unknown

##### Habitat

Habitat importance: Major Importance

Habitats: 13.3. Marine Coastal/Supratidal - Coastal Sand Dunes

#### Habitat

Habitat importance: Major Importance

Habitats: 13.3. Marine Coastal/Supratidal - Coastal Sand Dunes

#### Ecology

Size: 4 mm

Generation length (yr): 1

Dependency of single sp?: No

Ecology and traits (narrative): Diurnal active hunters that reside in both ground and vegetation levels. They eat a variety of small invertebrates and construct no web.

#### Threats

Justification for threats: The existence of threats is unknown for this species.

##### Threats

Threat type: Past

Threats: 12. Other options - Other threat

#### Threats

Threat type: Past

Threats: 12. Other options - Other threat

#### Conservation

Justification for conservation actions: It is unknown exactly where this spider was collected but it is fair to assume that its true range might be partially or completely covered by the Natura 2000 network (PTZPE0047 and PTCON0018). It might also be totally or partially covered by the Sapal de Castro Marim e Vila Real de Santo António Natural Reserve.

##### Conservation actions

Conservation action type: In Place

Conservation actions: 1.1. Land/water protection - Site/area protection1.2. Land/water protection - Resource & habitat protection

#### Conservation actions

Conservation action type: In Place

Conservation actions: 1.1. Land/water protection - Site/area protection1.2. Land/water protection - Resource & habitat protection

#### Other

##### Use and trade

Use type: International

##### Ecosystem services

Ecosystem service type: Very important

##### Research needed

Research needed: 1.2. Research - Population size, distribution & trends1.3. Research - Life history & ecology1.5. Research - Threats

Justification for research needed: Research is needed on basic information such as distribution, ecology, life cycle and possible threats throughout the range.

#### Use and trade

Use type: International

#### Ecosystem services

Ecosystem service type: Very important

#### Research needed

Research needed: 1.2. Research - Population size, distribution & trends1.3. Research - Life history & ecology1.5. Research - Threats

Justification for research needed: Research is needed on basic information such as distribution, ecology, life cycle and possible threats throughout the range.

#### Viability analysis

### Ariadna inops

#### Species information

Scientific name: Ariadna inops

Species authority: Wunderlich, 2011

Kingdom: Animalia

Phylum: Arthropoda

Class: Arachnida

Order: Araneae

Family: Segestriidae

Region for assessment: Global

#### Geographic range

Biogeographic realm: Palearctic

Countries: Portugal

Map of records (Google Earth): Suppl. material 31

Basis of EOO and AOO: Unknown

Basis (narrative): There is only one record for the species (Wunderlich 2011). The true range is therefore unknown and not possible to model with confidence.

Min Elevation/Depth (m): 0

Max Elevation/Depth (m): 0

Range description: This spider is known from only one site, located on a beach on a peninsula near Manta Rota, Faro (Wunderlich 2011).

#### New occurrences

#### Extent of occurrence

EOO (km2): Unknown

Trend: Unknown

Causes ceased?: Unknown

Causes understood?: Unknown

Causes reversible?: Unknown

Extreme fluctuations?: Unknown

#### Area of occupancy

Trend: Unknown

Causes ceased?: Unknown

Causes understood?: Unknown

Causes reversible?: Unknown

Extreme fluctuations?: Unknown

AOO (km2): Unknown

#### Locations

Number of locations: Unknown

Justification for number of locations: Data available (1 record) are not enough to estimate the number of locations.

Trend: Unknown

Extreme fluctuations?: Unknown

#### Population

Number of individuals: Unknown

Trend: Unknown

Causes ceased?: Unknown

Causes understood?: Unknown

Causes reversible?: Unknown

Extreme fluctuations?: Unknown

Population Information (Narrative): No estimates of population size exist.

#### Subpopulations

Number of subpopulations: Unknown

Trend: Unknown

Justification for trend: Data available (1 record) are not enough to estimate the number of subpopulations.

Extreme fluctuations?: Unknown

Severe fragmentation?: Unknown

#### Habitat

System: Terrestrial

Habitat specialist: Unknown

Habitat (narrative): This spider is known from only one site, located on a beach.

Trend in extent, area or quality?: Unknown

##### Habitat

Habitat importance: Major Importance

Habitats: 13.3. Marine Coastal/Supratidal - Coastal Sand Dunes

#### Habitat

Habitat importance: Major Importance

Habitats: 13.3. Marine Coastal/Supratidal - Coastal Sand Dunes

#### Ecology

Size: 5.0 mm

Generation length (yr): 1

Dependency of single sp?: No

Ecology and traits (narrative): If similar to congeners, a ground tube-web builder that is active during both night and day.

#### Threats

Justification for threats: The existence of threats is unknown for this species.

##### Threats

Threat type: Past

Threats: 12. Other options - Other threat

#### Threats

Threat type: Past

Threats: 12. Other options - Other threat

#### Conservation

Justification for conservation actions: It is unknown exactly where this spider was collected, but it is fair to assume that its true range might be completely or at least partially covered by the Natura 2000 network (PTZPE0017 and PTCON0013). It might also be totally or partially covered by the Ria Formosa Natural Park.

##### Conservation actions

Conservation action type: In Place

Conservation actions: 1.1. Land/water protection - Site/area protection1.2. Land/water protection - Resource & habitat protection

#### Conservation actions

Conservation action type: In Place

Conservation actions: 1.1. Land/water protection - Site/area protection1.2. Land/water protection - Resource & habitat protection

#### Other

##### Use and trade

Use type: International

##### Ecosystem services

Ecosystem service type: Very important

##### Research needed

Research needed: 1.2. Research - Population size, distribution & trends1.3. Research - Life history & ecology1.5. Research - Threats

Justification for research needed: Research is needed on basic information such as distribution, ecology, life cycle and possible threats throughout the range.

#### Use and trade

Use type: International

#### Ecosystem services

Ecosystem service type: Very important

#### Research needed

Research needed: 1.2. Research - Population size, distribution & trends1.3. Research - Life history & ecology1.5. Research - Threats

Justification for research needed: Research is needed on basic information such as distribution, ecology, life cycle and possible threats throughout the range.

#### Viability analysis

### Lasaeola algarvensis

#### Species information

Scientific name: Lasaeola algarvensis

Species authority: Wunderlich, 2011

Kingdom: Animalia

Phylum: Arthropoda

Class: Arachnida

Order: Araneae

Family: Theridiidae

Region for assessment: Global

#### Geographic range

Biogeographic realm: Palearctic

Countries: Portugal

Map of records (Google Earth): Suppl. material 32

Basis of EOO and AOO: Unknown

Basis (narrative): There is only one record for the species (Wunderlich 2011) near a beach in Aljezur, Faro. The true range is therefore unknown and not possible to model with confidence.

Min Elevation/Depth (m): 20

Max Elevation/Depth (m): 20

Range description: This spider is known from only one site located next to a beach in Aljezur, Algarve, Southern Portugal (Wunderlich 2011).

#### New occurrences

#### Extent of occurrence

EOO (km2): Unknown

Trend: Unknown

Causes ceased?: Unknown

Causes understood?: Unknown

Causes reversible?: Unknown

Extreme fluctuations?: Unknown

#### Area of occupancy

Trend: Unknown

Causes ceased?: Unknown

Causes understood?: Unknown

Causes reversible?: Unknown

Extreme fluctuations?: Unknown

AOO (km2): Unknown

#### Locations

Number of locations: Unknown

Justification for number of locations: Data available (1 record) are not enough to estimate the number of locations.

Trend: Unknown

Extreme fluctuations?: No

#### Population

Number of individuals: Unknown

Trend: Unknown

Causes ceased?: Unknown

Causes understood?: Unknown

Causes reversible?: Unknown

Extreme fluctuations?: Unknown

Population Information (Narrative): No estimates of population size exist.

#### Subpopulations

Number of subpopulations: Unknown

Trend: Unknown

Extreme fluctuations?: Unknown

Severe fragmentation?: Unknown

#### Habitat

System: Terrestrial

Habitat specialist: Unknown

Habitat (narrative): This spider is known from only one site located next to a beach. The habitat of the site itself, where the specimen was found, is not specified, but it is assumed based on its location to be sand dunes.

Trend in extent, area or quality?: Unknown

##### Habitat

Habitat importance: Major Importance

Habitats: 13.3. Marine Coastal/Supratidal - Coastal Sand Dunes

#### Habitat

Habitat importance: Major Importance

Habitats: 13.3. Marine Coastal/Supratidal - Coastal Sand Dunes

#### Ecology

Size: 1.3 - 1.5 m

Generation length (yr): 1

Dependency of single sp?: No

Ecology and traits (narrative): If similar to congeners with close distribution, it inhabits dry places. Could limit itself to low vegetation in the dunes like *Lasaeola armona*, but more records could also reveal a preference for sandy pine groves. Could possess a capture web like *Lasaeola armona* or construct no web like *Lasaeola convexa*. Period of activity is unknown. Unknown if euryphagous or stenophagous.

#### Threats

Justification for threats: The existence of threats is unknown for this species.

##### Threats

Threat type: Past

Threats: 12. Other options - Other threat

#### Threats

Threat type: Past

Threats: 12. Other options - Other threat

#### Conservation

Justification for conservation actions: It is unknown exactly where this spider was collected, but it is fair to assume that its true range might be completely or at least partially covered by the Sudoeste Alentejano e Costa Vicentina Natural Park. It might also be totally or partially covered by the Natura 2000 network (PTZPE0015 and PTCON0012).

##### Conservation actions

Conservation action type: In Place

Conservation actions: 1.1. Land/water protection - Site/area protection1.2. Land/water protection - Resource & habitat protection

#### Conservation actions

Conservation action type: In Place

Conservation actions: 1.1. Land/water protection - Site/area protection1.2. Land/water protection - Resource & habitat protection

#### Other

##### Use and trade

Use type: International

##### Ecosystem services

Ecosystem service type: Very important

##### Research needed

Research needed: 1.2. Research - Population size, distribution & trends1.3. Research - Life history & ecology1.5. Research - Threats

Justification for research needed: Research is needed on basic information such as distribution, ecology, life cycle and possible threats throughout the range.

#### Use and trade

Use type: International

#### Ecosystem services

Ecosystem service type: Very important

#### Research needed

Research needed: 1.2. Research - Population size, distribution & trends1.3. Research - Life history & ecology1.5. Research - Threats

Justification for research needed: Research is needed on basic information such as distribution, ecology, life cycle and possible threats throughout the range.

#### Viability analysis

### Theridion bernardi

#### Species information

Scientific name: Theridion bernardi

Species authority: Lecigne, 2017

Kingdom: Animalia

Phylum: Arthropoda

Class: Arachnida

Order: Araneae

Family: Theridiidae

Region for assessment: Global

#### Geographic range

Biogeographic realm: Palearctic

Countries: Portugal

Map of records (Google Earth): Suppl. material 33

Basis of EOO and AOO: Unknown

Basis (narrative): There is only one record for the species (Lecigne 2017). The true range is therefore unknown and not possible to model with confidence.

Min Elevation/Depth (m): 17

Max Elevation/Depth (m): 23

Range description: This spider is known from only one site of unspecified habitat, at the village of Olhos de Água, Algarve, Southern Portugal (Lecigne 2017).

#### New occurrences

#### Extent of occurrence

EOO (km2): Unknown

Trend: Unknown

Causes ceased?: Unknown

Causes understood?: Unknown

Causes reversible?: Unknown

Extreme fluctuations?: Unknown

#### Area of occupancy

Trend: Unknown

Causes ceased?: Unknown

Causes understood?: Unknown

Causes reversible?: Unknown

Extreme fluctuations?: Unknown

AOO (km2): Unknown

#### Locations

Number of locations: Unknown

Justification for number of locations: Data available (1 record) are not enough to estimate the number of locations.

Trend: Unknown

Extreme fluctuations?: Unknown

#### Population

Number of individuals: Unknown

Trend: Unknown

Causes ceased?: Unknown

Causes understood?: Unknown

Causes reversible?: Unknown

Extreme fluctuations?: Unknown

Population Information (Narrative): No estimates of population size exist.

#### Subpopulations

Number of subpopulations: Unknown

Trend: Unknown

Extreme fluctuations?: Unknown

Severe fragmentation?: Unknown

#### Habitat

System: Terrestrial

Habitat specialist: Unknown

Habitat (narrative): This spider is known from only one site: a hotel complex next to shrubland in the village of Olhos de Água. The holotype was collected by beating bushes in the shrubland immediately adjacent to the hotel complex where the paratype was found, standing on a wall.

Trend in extent, area or quality?: Unknown

Justification for trend: There are no currently known threats to the species.

##### Habitat

Habitat importance: Major Importance

Habitats: 3.8. Shrubland - Mediterranean-type Shrubby Vegetation14.5. Artificial/Terrestrial - Urban Areas

#### Habitat

Habitat importance: Major Importance

Habitats: 3.8. Shrubland - Mediterranean-type Shrubby Vegetation14.5. Artificial/Terrestrial - Urban Areas

#### Ecology

Size: 2.82 - 3.47 mm

Generation length (yr): 1

Dependency of single sp?: No

Ecology and traits (narrative): In general, species of this genus build tridimensional webs to capture their prey.

#### Threats

Justification for threats: The existence of threats is unknown for this species.

##### Threats

Threat type: Past

Threats: 12. Other options - Other threat

#### Threats

Threat type: Past

Threats: 12. Other options - Other threat

#### Conservation

Justification for conservation actions: This spider has not been recorded in areas inside or adjacent to protected areas. More records are needed in order to confirm or disprove this for the species' true range.

##### Conservation actions

Conservation action type: Needed

Conservation actions: 1.1. Land/water protection - Site/area protection1.2. Land/water protection - Resource & habitat protection

#### Conservation actions

Conservation action type: Needed

Conservation actions: 1.1. Land/water protection - Site/area protection1.2. Land/water protection - Resource & habitat protection

#### Other

##### Use and trade

Use type: International

##### Ecosystem services

Ecosystem service type: Very important

##### Research needed

Research needed: 1.2. Research - Population size, distribution & trends1.3. Research - Life history & ecology1.5. Research - Threats

Justification for research needed: Research is needed on basic information such as distribution, ecology, life cycle and possible threats throughout the range.

#### Use and trade

Use type: International

#### Ecosystem services

Ecosystem service type: Very important

#### Research needed

Research needed: 1.2. Research - Population size, distribution & trends1.3. Research - Life history & ecology1.5. Research - Threats

Justification for research needed: Research is needed on basic information such as distribution, ecology, life cycle and possible threats throughout the range.

#### Viability analysis

### Amphiledorus ungoliantae

#### Species information

Scientific name: Amphiledorus ungoliantae

Species authority: Pekár & Cardoso, 2005

Common names: Aranha-de-tolkien

Kingdom: Animalia

Phylum: Arthropoda

Class: Arachnida

Order: Araneae

Family: Zodariidae

Region for assessment: Global

#### Geographic range

Biogeographic realm: Palearctic

Countries: Portugal

Map of records (Google Earth): Suppl. material 34

Basis of EOO and AOO: Unknown

Basis (narrative): There is only one record for the species (Pekár and Cardoso 2005) from the small village of Corte da Velha, in the Vale of Guadiana Natural Park, south-eastern Portugal. The true range is therefore unknown and not possible to model with confidence.

Min Elevation/Depth (m): 170

Max Elevation/Depth (m): 170

Range description: This spider is known from only one scrubland dominated by *Cystus* sp. close to the small village of Corte da Velha, in the Vale of Guadiana Natural Park, south-eastern Portugal (Pekár and Cardoso 2005).

#### New occurrences

#### Extent of occurrence

EOO (km2): Unknown

Trend: Unknown

Causes ceased?: Unknown

Causes understood?: Unknown

Causes reversible?: Unknown

Extreme fluctuations?: Unknown

#### Area of occupancy

Trend: Unknown

Causes ceased?: Unknown

Causes understood?: Unknown

Causes reversible?: Unknown

Extreme fluctuations?: Unknown

AOO (km2): Unknown

#### Locations

Number of locations: Unknown

Justification for number of locations: Data available (1 record) are not enough to estimate the number of locations.

Trend: Unknown

Extreme fluctuations?: Unknown

#### Population

Number of individuals: Unknown

Trend: Unknown

Causes ceased?: Unknown

Causes understood?: Unknown

Causes reversible?: Unknown

Extreme fluctuations?: Unknown

Population Information (Narrative): No estimates of population size exist.

#### Subpopulations

Number of subpopulations: Unknown

Trend: Unknown

Extreme fluctuations?: Unknown

Severe fragmentation?: Unknown

#### Habitat

System: Terrestrial

Habitat specialist: Unknown

Habitat (narrative): This spider is only known from a scrubland dominated by *Cystus* sp.

Trend in extent, area or quality?: Unknown

##### Habitat

Habitat importance: Major Importance

Habitats: 3.8. Shrubland - Mediterranean-type Shrubby Vegetation

#### Habitat

Habitat importance: Major Importance

Habitats: 3.8. Shrubland - Mediterranean-type Shrubby Vegetation

#### Ecology

Size: 7.8 - 8.71 mm

Generation length (yr): 1

Dependency of single sp?: Unknown

Ecology and traits (narrative): The genus Amphiledorus has been recently described so not much information is available. However, a single species, *Amphiledorus histrionicus* (Simon, 1884) was once part of genus Selamia, members of which are described as hiding during the day in sand-covered silken retreats that serve at the same time as both hiding corners and capturing devices, with prey being ambushed from underneath the retreat (Jocqué and Bosmans 2001).

#### Threats

Justification for threats: The existence of threats is unknown for this species.

##### Threats

Threat type: Past

Threats: 12. Other options - Other threat

#### Threats

Threat type: Past

Threats: 12. Other options - Other threat

#### Conservation

Justification for conservation actions: The only known locality for this spider is inside the Vale do Guadiana Natural Park and its true range might be partially or completely covered by the Natura 2000 network (PTZPE0047 and PTCON0036).

##### Conservation actions

Conservation action type: In Place

Conservation actions: 1.1. Land/water protection - Site/area protection1.2. Land/water protection - Resource & habitat protection

#### Conservation actions

Conservation action type: In Place

Conservation actions: 1.1. Land/water protection - Site/area protection1.2. Land/water protection - Resource & habitat protection

#### Other

##### Use and trade

Use type: International

##### Ecosystem services

Ecosystem service type: Very important

##### Research needed

Research needed: 1.2. Research - Population size, distribution & trends1.3. Research - Life history & ecology1.5. Research - Threats

Justification for research needed: Research is needed on basic information such as distribution, ecology, life cycle and possible threats throughout the range.

#### Use and trade

Use type: International

#### Ecosystem services

Ecosystem service type: Very important

#### Research needed

Research needed: 1.2. Research - Population size, distribution & trends1.3. Research - Life history & ecology1.5. Research - Threats

Justification for research needed: Research is needed on basic information such as distribution, ecology, life cycle and possible threats throughout the range.

#### Viability analysis

### Zodarion alentejanum

#### Species information

Scientific name: Zodarion alentejanum

Species authority: Pekár & Carvalho, 2011

Kingdom: Animalia

Phylum: Arthropoda

Class: Arachnida

Order: Araneae

Family: Zodariidae

Region for assessment: Global

#### Geographic range

Biogeographic realm: Palearctic

Countries: Portugal

Map of records (Google Earth): Suppl. material 35

Basis of EOO and AOO: Unknown

Basis (narrative): There is only one record for the species (Pekár et al. 2011). The true range is therefore unknown and not possible to model with confidence.

Min Elevation/Depth (m): 10

Max Elevation/Depth (m): 10

Range description: This spider is known from only one site, in sand dunes (Pekár et al. 2011) in southwest Portugal. The true range is therefore unknown and not possible to model with confidence.

#### New occurrences

#### Extent of occurrence

EOO (km2): Unknown

Trend: Unknown

Causes ceased?: Unknown

Causes understood?: Unknown

Causes reversible?: Unknown

Extreme fluctuations?: Unknown

#### Area of occupancy

Trend: Unknown

Causes ceased?: Unknown

Causes understood?: Unknown

Causes reversible?: Unknown

Extreme fluctuations?: Unknown

AOO (km2): Unknown

#### Locations

Number of locations: Unknown

Justification for number of locations: Data available (1 record) are not enough to estimate the number of locations.

Trend: Unknown

Extreme fluctuations?: Unknown

#### Population

Number of individuals: Unknown

Trend: Unknown

Causes ceased?: Unknown

Causes understood?: Unknown

Causes reversible?: Unknown

Extreme fluctuations?: Unknown

Population Information (Narrative): No estimates of population size exist.

#### Subpopulations

Number of subpopulations: Unknown

Trend: Unknown

Extreme fluctuations?: Unknown

Severe fragmentation?: Unknown

#### Habitat

System: Terrestrial

Habitat specialist: Unknown

Habitat (narrative): This spider is known from only one site in sand dunes.

Trend in extent, area or quality?: Unknown

##### Habitat

Habitat importance: Major Importance

Habitats: 13.3. Marine Coastal/Supratidal - Coastal Sand Dunes

#### Habitat

Habitat importance: Major Importance

Habitats: 13.3. Marine Coastal/Supratidal - Coastal Sand Dunes

#### Ecology

Size: 3.3 - 4.1 mm

Generation length (yr): 1

Dependency of single sp?: Unknown

Ecology and traits (narrative): An ant-eating species that produces no web and uses specialised predator behaviour and mimicry in order to capture its prey.

#### Threats

Justification for threats: The existence of threats is unknown for this species.

##### Threats

Threat type: Past

Threats: 12. Other options - Other threat

#### Threats

Threat type: Past

Threats: 12. Other options - Other threat

#### Conservation

Justification for conservation actions: Although the species' true range is unknown, it was recorded once in the Lagoas de Santo André Natural Reserve, an area covered by the Natura 2000 network (PTZPE0014; PTCON0034).

##### Conservation actions

Conservation action type: In Place

Conservation actions: 1.1. Land/water protection - Site/area protection1.2. Land/water protection - Resource & habitat protection

#### Conservation actions

Conservation action type: In Place

Conservation actions: 1.1. Land/water protection - Site/area protection1.2. Land/water protection - Resource & habitat protection

#### Other

##### Use and trade

Use type: International

##### Ecosystem services

Ecosystem service type: Very important

##### Research needed

Research needed: 1.2. Research - Population size, distribution & trends1.3. Research - Life history & ecology1.5. Research - Threats

Justification for research needed: Research is needed on basic information such as distribution, ecology, life cycle and possible threats throughout the range.

#### Use and trade

Use type: International

#### Ecosystem services

Ecosystem service type: Very important

#### Research needed

Research needed: 1.2. Research - Population size, distribution & trends1.3. Research - Life history & ecology1.5. Research - Threats

Justification for research needed: Research is needed on basic information such as distribution, ecology, life cycle and possible threats throughout the range.

#### Viability analysis

### Zodarion algarvense

#### Species information

Scientific name: Zodarion algarvense

Species authority: Bosmans, 1994

Kingdom: Animalia

Phylum: Arthropoda

Class: Arachnida

Order: Araneae

Family: Zodariidae

Region for assessment: Global

#### Geographic range

Biogeographic realm: Palearctic

Countries: Portugal

Map of records (Google Earth): Suppl. material 36

Basis of EOO and AOO: Species Distribution Model

Basis (narrative): There are only three collection sites recorded for this species (3 records), mostly recent and always in sand dunes (Bosmans 1994, Carvalho et al. 2011, Pekár et al. 2011). The true range is therefore unknown and not possible to model with confidence.

Min Elevation/Depth (m): 0

Max Elevation/Depth (m): 376

Range description: This spider is known from only three sites in southern Portugal, always in sand dunes (Bosmans 1994, Carvalho et al. 2011, Pekár et al. 2011).

#### New occurrences

#### Extent of occurrence

EOO (km2): Unknown

Trend: Unknown

Causes ceased?: Unknown

Causes understood?: Unknown

Causes reversible?: Unknown

Extreme fluctuations?: Unknown

#### Area of occupancy

Trend: Unknown

Causes ceased?: Unknown

Causes understood?: Unknown

Causes reversible?: Unknown

Extreme fluctuations?: Unknown

AOO (km2): Unknown

#### Locations

Number of locations: Unknown

Justification for number of locations: Data available (3 sites) are not enough to estimate the number of locations.

Trend: Unknown

Extreme fluctuations?: Unknown

#### Population

Number of individuals: Unknown

Trend: Decline (inferred)

Justification for trend: The sand dunes, in which this species has been found, are delicate habitats that are threatened by habitat loss and possible increase in number of extreme weather events due to climate change.

Basis for decline: (c) a decline in area of occupancy, extent of occurrence and/or quality of habitat

Causes ceased?: No

Causes understood?: Yes

Causes reversible?: No

Extreme fluctuations?: No

Population Information (Narrative): No estimates of population size exist.

#### Subpopulations

Number of subpopulations: Unknown

Trend: Decline (inferred)

Justification for trend: The sand dunes, in which this species has been found, are delicate habitats that are threatened by habitat loss and possible increase in number of extreme weather events due to climate change.

Extreme fluctuations?: No

Severe fragmentation?: No

#### Habitat

System: Terrestrial

Habitat specialist: Yes

Habitat (narrative): This spider has been found so far exclusively on sand dunes. All records are associated with sand dune habitats except for the record from the Algarve which, while possessing no habitat information, is located on a coastal area.

Trend in extent, area or quality?: Decline (inferred)

Justification for trend: The sand dunes, in which this species has been found, are delicate habitats that are threatened by habitat loss and possible increase in number of extreme weather events due to climate change.

##### Habitat

Habitat importance: Major Importance

Habitats: 13.3. Marine Coastal/Supratidal - Coastal Sand Dunes

#### Habitat

Habitat importance: Major Importance

Habitats: 13.3. Marine Coastal/Supratidal - Coastal Sand Dunes

#### Ecology

Size: 2.4 - 3.6 mm

Generation length (yr): 1

Dependency of single sp?: Unknown

Ecology and traits (narrative): An ant-eating species that produces no web and uses specialised predator behaviour and mimicry in order to capture its prey.

#### Threats

Justification for threats: The sand dunes, in which this species is found, are delicate habitats that are threatened by habitat loss and possible increase in number of extreme weather events due to climate change.

##### Threats

Threat type: Ongoing

Threats: 1.1. Residential & commercial development - Housing & urban areas1.2. Residential & commercial development - Commercial & industrial areas1.3. Residential & commercial development - Tourism & recreation areas11.4. Climate change & severe weather - Storms & flooding

#### Threats

Threat type: Ongoing

Threats: 1.1. Residential & commercial development - Housing & urban areas1.2. Residential & commercial development - Commercial & industrial areas1.3. Residential & commercial development - Tourism & recreation areas11.4. Climate change & severe weather - Storms & flooding

#### Conservation

Justification for conservation actions: This spider has two of its records inside the Lagoas de Santo André e Sancha Natural Park and the areas, in which it has currently been recorded, are inside the Natura 2000 network (PTCON0034; PTZPE0014; PTZPE0013). Additionally, all beaches in Portugal are governed by the European Water Framework Directive (directive 2000/60/EC) enacted through land-use plans that conserve and defend coastal ecosystems (decree-law Nº 130/2012).

##### Conservation actions

Conservation action type: In Place

Conservation actions: 1.1. Land/water protection - Site/area protection1.2. Land/water protection - Resource & habitat protection

#### Conservation actions

Conservation action type: In Place

Conservation actions: 1.1. Land/water protection - Site/area protection1.2. Land/water protection - Resource & habitat protection

#### Other

##### Use and trade

Use type: International

##### Ecosystem services

Ecosystem service type: Very important

##### Research needed

Research needed: 3.1. Monitoring - Population trends3.4. Monitoring - Habitat trends

Justification for research needed: Research is needed on basic information such as distribution, ecology, life cycle and possible threats throughout the range.

#### Use and trade

Use type: International

#### Ecosystem services

Ecosystem service type: Very important

#### Research needed

Research needed: 3.1. Monitoring - Population trends3.4. Monitoring - Habitat trends

Justification for research needed: Research is needed on basic information such as distribution, ecology, life cycle and possible threats throughout the range.

#### Viability analysis

### Zodarion bacelarae

#### Species information

Scientific name: Zodarion bacelarae

Species authority: Pekár, 2003

Kingdom: Animalia

Phylum: Arthropoda

Class: Arachnida

Order: Araneae

Family: Zodariidae

Region for assessment: Global

#### Geographic range

Biogeographic realm: Palearctic

Countries: Portugal

Map of records (Google Earth): Suppl. material 37

Basis of EOO and AOO: Unknown

Basis (narrative): There is only one record for the species (Pekár et al. 2003). The true range is therefore unknown and not possible to model with confidence.

Min Elevation/Depth (m): 400

Max Elevation/Depth (m): 400

Range description: This spider is known from only one site of unspecified habitat in the Torre de Moncorvo municipality, north-eastern Portugal.

#### New occurrences

#### Extent of occurrence

EOO (km2): Unknown

Trend: Unknown

Causes ceased?: Unknown

Causes understood?: Unknown

Causes reversible?: Unknown

Extreme fluctuations?: Unknown

#### Area of occupancy

Trend: Unknown

Causes ceased?: Unknown

Causes understood?: Unknown

Causes reversible?: Unknown

Extreme fluctuations?: Unknown

AOO (km2): Unknown

#### Locations

Number of locations: Unknown

Justification for number of locations: Data available (1 record) are not enough to estimate the number of locations.

Trend: Unknown

Extreme fluctuations?: Unknown

#### Population

Number of individuals: Unknown

Trend: Unknown

Causes ceased?: Unknown

Causes understood?: Unknown

Causes reversible?: Unknown

Extreme fluctuations?: Unknown

Population Information (Narrative): No estimates of population size exist.

#### Subpopulations

Number of subpopulations: Unknown

Trend: Unknown

Justification for trend: Data available (1 record) are not enough to estimate the number of subpopulations.

Extreme fluctuations?: Unknown

Severe fragmentation?: Unknown

#### Habitat

System: Terrestrial

Habitat specialist: Unknown

Habitat (narrative): Habitat is unknown. This spider has been recorded only once in an area of unspecified habitat.

Trend in extent, area or quality?: Unknown

##### Habitat

Habitat importance: Suitable

Habitats: 18. Unknown

#### Habitat

Habitat importance: Suitable

Habitats: 18. Unknown

#### Ecology

Size: 4.6

Generation length (yr): 1

Dependency of single sp?: Unknown

Ecology and traits (narrative): An ant-eating species that produces no web and uses specialised predator behaviour and mimicry in order to capture its prey.

#### Threats

Justification for threats: The existence of threats is unknown for this species.

##### Threats

Threat type: Past

Threats: 12. Other options - Other threat

#### Threats

Threat type: Past

Threats: 12. Other options - Other threat

#### Conservation

Justification for conservation actions: This spider has not been recorded in areas inside or adjacent to protected areas, but it is close to the Douro International Natural Park. More records are needed in order to confirm or disprove this for the species' true range.

##### Conservation actions

Conservation action type: Needed

Conservation actions: 1.1. Land/water protection - Site/area protection1.2. Land/water protection - Resource & habitat protection

#### Conservation actions

Conservation action type: Needed

Conservation actions: 1.1. Land/water protection - Site/area protection1.2. Land/water protection - Resource & habitat protection

#### Other

##### Use and trade

Use type: International

##### Ecosystem services

Ecosystem service type: Very important

##### Research needed

Research needed: 1.2. Research - Population size, distribution & trends1.3. Research - Life history & ecology1.5. Research - Threats

Justification for research needed: Research is needed on basic information such as distribution, ecology, life cycle and possible threats throughout the range.

#### Use and trade

Use type: International

#### Ecosystem services

Ecosystem service type: Very important

#### Research needed

Research needed: 1.2. Research - Population size, distribution & trends1.3. Research - Life history & ecology1.5. Research - Threats

Justification for research needed: Research is needed on basic information such as distribution, ecology, life cycle and possible threats throughout the range.

#### Viability analysis

### Zodarion bosmansi

#### Species information

Scientific name: Zodarion bosmansi

Species authority: Pekár & Cardoso, 2005

Kingdom: Animalia

Phylum: Arthropoda

Class: Arachnida

Order: Araneae

Family: Zodariidae

Region for assessment: Global

#### Geographic range

Biogeographic realm: Palearctic

Countries: Portugal

Map of records (Google Earth): Suppl. material 38

Basis of EOO and AOO: Species Distribution Model

Basis (narrative): Multiple collection sites (5 records) are recorded for this species, mostly recent, but with scarce habitat information (Pekár et al. 2003). It was possible to perform species distribution modelling to predict its potential range with confidence limits. See Methods for details.

Min Elevation/Depth (m): 0

Max Elevation/Depth (m): 353

Range description: This spider has been recorded in central and southern Portugal. The species distribution modelling predicts that this species could be widespread throughout the region and could be present in Spain along its southern border with Portugal.

#### New occurrences

#### Extent of occurrence

EOO (km2): 24484 - 37597

Trend: Stable

Justification for trend: There are no currently known threats to the species.

Causes ceased?: Yes

Causes understood?: Yes

Causes reversible?: Yes

Extreme fluctuations?: No

#### Area of occupancy

Trend: Stable

Justification for trend: There are no currently known threats to the species.

Causes ceased?: Yes

Causes understood?: Yes

Causes reversible?: Yes

Extreme fluctuations?: No

AOO (km2): 10812 - 19692

#### Locations

Number of locations: Not applicable

Justification for number of locations: There are no currently known threats to the species.

Trend: Stable

Extreme fluctuations?: Unknown

#### Population

Number of individuals: Unknown

Trend: Stable

Justification for trend: There are no currently known threats to the species.

Causes ceased?: Yes

Causes understood?: Yes

Causes reversible?: Yes

Extreme fluctuations?: No

Population Information (Narrative): No estimates of population size exist.

#### Subpopulations

Number of subpopulations: Unknown

Trend: Stable

Justification for trend: There are no currently known threats to the species.

Extreme fluctuations?: No

Severe fragmentation?: Unknown

#### Habitat

System: Terrestrial

Habitat specialist: No

Habitat (narrative): Habitat information has only been recorded twice for this spider, highlighting woods or grasslands, dominated by *Quercus ilex* and *Cystus* sp.

Trend in extent, area or quality?: Stable

Justification for trend: There are no currently known threats to the species.

##### Habitat

Habitat importance: Major Importance

Habitats: 1.4. Forest - Temperate4.4. Grassland - Temperate

#### Habitat

Habitat importance: Major Importance

Habitats: 1.4. Forest - Temperate4.4. Grassland - Temperate

#### Ecology

Size: 4.08 - 4.84 mm

Generation length (yr): 1

Dependency of single sp?: Unknown

Ecology and traits (narrative): An ant-eating species that produces no web and uses specialised predator behaviour and mimicry in order to capture its prey.

#### Threats

Justification for threats: The existence of threats is unknown for this species.

##### Threats

Threat type: Past

Threats: 12. Other options - Other threat

#### Threats

Threat type: Past

Threats: 12. Other options - Other threat

#### Conservation

Justification for conservation actions: This spider has been recorded twice inside the Vale do Guadiana Natural Park, an area covered by the Natura 2000 network (PTCON0036; PTZPE0047). Species distribution modelling predicts that it could be present in more protected areas.

##### Conservation actions

Conservation action type: In Place

Conservation actions: 1.1. Land/water protection - Site/area protection1.2. Land/water protection - Resource & habitat protection

#### Conservation actions

Conservation action type: In Place

Conservation actions: 1.1. Land/water protection - Site/area protection1.2. Land/water protection - Resource & habitat protection

#### Other

##### Use and trade

Use type: International

##### Ecosystem services

Ecosystem service type: Very important

##### Research needed

Research needed: 3.1. Monitoring - Population trends3.4. Monitoring - Habitat trends

Justification for research needed: Monitoring of population and habitat are important to confirm inferred trends.

#### Use and trade

Use type: International

#### Ecosystem services

Ecosystem service type: Very important

#### Research needed

Research needed: 3.1. Monitoring - Population trends3.4. Monitoring - Habitat trends

Justification for research needed: Monitoring of population and habitat are important to confirm inferred trends.

#### Viability analysis

### Zodarion costapratae

#### Species information

Scientific name: Zodarion costapratae

Species authority: Pekár, 2011

Kingdom: Animalia

Phylum: Chelicerata

Class: Arachnida

Order: Araneae

Family: Zodariidae

Region for assessment: Global

#### Geographic range

Biogeographic realm: Palearctic

Countries: Portugal

Map of records (Google Earth): Suppl. material 39

Basis of EOO and AOO: Species Distribution Model

Basis (narrative): Multiple collection sites are recorded for this species (6 records), mostly recent and in three distinct habitats (Pekár et al. 2011, Crespo 2008). It was possible to perform species distribution modelling to predict its potential range with confidence limits. See Methods for details.

Min Elevation/Depth (m): 0

Max Elevation/Depth (m): 567

Range description: This spider has been recorded in north and central Portugal along the coast. Species distribution modelling predicts that this species could cover the entirety of Portugal's north and central coast, from Lisbon to Viana do Castelo.

#### New occurrences

#### Extent of occurrence

EOO (km2): 22300 - 27556

Trend: Stable

Justification for trend: There are no currently known threats to the species.

Causes ceased?: Yes

Causes understood?: Yes

Causes reversible?: Yes

Extreme fluctuations?: No

#### Area of occupancy

Trend: Stable

Justification for trend: There are no currently known threats to the species.

Causes ceased?: Yes

Causes understood?: Yes

Causes reversible?: Yes

Extreme fluctuations?: No

AOO (km2): 9932 - 13396

#### Locations

Number of locations: Not applicable

Justification for number of locations: There are no currently known threats to the species.

Trend: Stable

Extreme fluctuations?: Unknown

#### Population

Number of individuals: Unknown

Trend: Stable

Justification for trend: There are no currently known threats to the species.

Causes ceased?: Yes

Causes understood?: Yes

Causes reversible?: Yes

Extreme fluctuations?: No

Population Information (Narrative): No estimates of population size exist.

#### Subpopulations

Number of subpopulations: Unknown

Trend: Stable

Justification for trend: There are no currently known threats to the species.

Extreme fluctuations?: No

Severe fragmentation?: Unknown

#### Habitat

System: Terrestrial

Habitat specialist: No

Habitat (narrative): This spider has been recorded in three distinct habitats: pinewood forests, sand dunes and marshes.

Trend in extent, area or quality?: Unknown

##### Habitat

Habitat importance: Major Importance

Habitats: 1.4. Forest - Temperate5.4. Wetlands (inland) - Bogs, Marshes, Swamps, Fens, Peatlands13.3. Marine Coastal/Supratidal - Coastal Sand Dunes

#### Habitat

Habitat importance: Major Importance

Habitats: 1.4. Forest - Temperate5.4. Wetlands (inland) - Bogs, Marshes, Swamps, Fens, Peatlands13.3. Marine Coastal/Supratidal - Coastal Sand Dunes

#### Ecology

Size: 2.2 - 3.7 mm

Generation length (yr): 1

Dependency of single sp?: Unknown

Ecology and traits (narrative): An ant-eating species that produces no web and uses specialised predator behaviour and mimicry in order to capture its prey.

#### Threats

Justification for threats: The existence of threats is unknown for this species.

##### Threats

Threat type: Past

Threats: 12. Other options - Other threat

#### Threats

Threat type: Past

Threats: 12. Other options - Other threat

#### Conservation

Justification for conservation actions: This spider has been recorded inside the Paul de Arzila Natural Reserve and the Serras de Aire e Candeeiros Natural Park, both areas covered by the Natura 2000 network (PTCON0005; PTZPE0005; PTCON0015). It could be present in numerous other protected areas.

##### Conservation actions

Conservation action type: In Place

Conservation actions: 1.1. Land/water protection - Site/area protection1.2. Land/water protection - Resource & habitat protection

#### Conservation actions

Conservation action type: In Place

Conservation actions: 1.1. Land/water protection - Site/area protection1.2. Land/water protection - Resource & habitat protection

#### Other

##### Use and trade

Use type: International

##### Ecosystem services

Ecosystem service type: Very important

##### Research needed

Research needed: 3.1. Monitoring - Population trends3.4. Monitoring - Habitat trends

Justification for research needed: Monitoring of population and habitat are important to confirm inferred trends.

#### Use and trade

Use type: International

#### Ecosystem services

Ecosystem service type: Very important

#### Research needed

Research needed: 3.1. Monitoring - Population trends3.4. Monitoring - Habitat trends

Justification for research needed: Monitoring of population and habitat are important to confirm inferred trends.

#### Viability analysis

### Zodarion duriense

#### Species information

Scientific name: Zodarion duriense

Species authority: Cardoso, 2003

Kingdom: Animalia

Phylum: Arthropoda

Class: Arachnida

Order: Araneae

Family: Zodariidae

Region for assessment: Global

#### Geographic range

Biogeographic realm: Palearctic

Countries: Portugal

Map of records (Google Earth): Suppl. material 40

Basis of EOO and AOO: Species Distribution Model

Basis (narrative): Multiple collection sites are recorded for this species (11 records), mostly recent and in a variety of habitats (Pekár et al. 2003, Cardoso 2004, Sousa 2006, Pekár et al. 2011, Benhadi-Marin et al. 2018). It was possible to perform species distribution modelling to predict its potential range with confidence limits. See Methods for details.

Min Elevation/Depth (m): 50

Max Elevation/Depth (m): 2000

Range description: This spider has been recorded several times throughout northern Portugal, most of which were in the northeast. The species distribution modelling predicts that the species could occupy areas closer to the coast, as well as to be present in north-western Spain.

#### New occurrences

#### Extent of occurrence

EOO (km2): 69440 - 79270

Trend: Stable

Justification for trend: There are no currently known threats to the species.

Causes ceased?: Yes

Causes understood?: Yes

Causes reversible?: Yes

Extreme fluctuations?: No

#### Area of occupancy

Trend: Stable

Justification for trend: There are no currently known threats to the species.

Causes ceased?: Yes

Causes understood?: Yes

Causes reversible?: Yes

Extreme fluctuations?: No

AOO (km2): 45920 - 55088

#### Locations

Number of locations: Not applicable

Justification for number of locations: There are no currently known threats to the species.

Trend: Stable

Extreme fluctuations?: Unknown

#### Population

Number of individuals: Unknown

Trend: Stable

Causes ceased?: Yes

Causes understood?: Yes

Causes reversible?: Yes

Extreme fluctuations?: No

Population Information (Narrative): No estimates of population size exist.

#### Subpopulations

Trend: Stable

Justification for trend: There are no currently known threats to the species.

Extreme fluctuations?: No

Severe fragmentation?: No

#### Habitat

System: Terrestrial

Habitat specialist: No

Habitat (narrative): This spider has been found in a variety of habitats including olive groves, shrublands (with *Cystus* sp., *Cytisus* sp. or *Genista* sp.), forests (*Quercus* spp.) and plantations (*Eucalyptus* sp., *Pinus* spp.).

Trend in extent, area or quality?: Stable

Justification for trend: There are no currently known threats to the species.

##### Habitat

Habitat importance: Major Importance

Habitats: 1.4. Forest - Temperate3.8. Shrubland - Mediterranean-type Shrubby Vegetation

##### Habitat

Habitat importance: Suitable

Habitats: 16. Introduced vegetation

#### Habitat

Habitat importance: Major Importance

Habitats: 1.4. Forest - Temperate3.8. Shrubland - Mediterranean-type Shrubby Vegetation

#### Habitat

Habitat importance: Suitable

Habitats: 16. Introduced vegetation

#### Ecology

Size: 3.4 - 6.1 mm

Generation length (yr): 1

Dependency of single sp?: Unknown

Ecology and traits (narrative): An ant-eating species that produces no web and uses specialised predator behaviour and mimicry in order to capture its prey.

#### Threats

Justification for threats: The existence of threats is unknown for this species.

##### Threats

Threat type: Past

Threats: 12. Other options - Other threat

#### Threats

Threat type: Past

Threats: 12. Other options - Other threat

#### Conservation

Justification for conservation actions: This spider has been recorded in areas located inside the Serra da Estrela Natural Park and the Douro Internacional Natural Park. These areas are in turn currently covered by the Natura 2000 network (PTCON0014; ES0000118; ES4150096). The species could, however, be more widespread and occupy other protected areas in Portugal and Spain.

##### Conservation actions

Conservation action type: In Place

Conservation actions: 1.1. Land/water protection - Site/area protection1.2. Land/water protection - Resource & habitat protection

#### Conservation actions

Conservation action type: In Place

Conservation actions: 1.1. Land/water protection - Site/area protection1.2. Land/water protection - Resource & habitat protection

#### Other

##### Use and trade

Use type: International

##### Ecosystem services

Ecosystem service type: Very important

##### Research needed

Research needed: 3.1. Monitoring - Population trends3.4. Monitoring - Habitat trends

Justification for research needed: Monitoring of population and habitat are important to confirm inferred trends.

#### Use and trade

Use type: International

#### Ecosystem services

Ecosystem service type: Very important

#### Research needed

Research needed: 3.1. Monitoring - Population trends3.4. Monitoring - Habitat trends

Justification for research needed: Monitoring of population and habitat are important to confirm inferred trends.

#### Viability analysis

### Zodarion guadianense

#### Species information

Scientific name: Zodarion guadianense

Species authority: Cardoso, 2003

Kingdom: Animalia

Phylum: Arthropoda

Class: Arachnida

Order: Araneae

Family: Zodariidae

Region for assessment: Global

#### Geographic range

Biogeographic realm: Palearctic

Countries: Portugal

Map of records (Google Earth): Suppl. material 41

Basis of EOO and AOO: Unknown

Basis (narrative): Unknown, as there is only one record for the species (Pekár et al. 2003) in Vale do Guadiana Natural Park, Beja. The species' true range is, therefore, unknown and not possible to model with confidence.

Min Elevation/Depth (m): 120

Max Elevation/Depth (m): 120

Range description: This spider is known from only one scrubland site, dominated by *Cistus* sp. in south-eastern Portugal.

#### New occurrences

#### Extent of occurrence

EOO (km2): Unknown

Trend: Unknown

Causes ceased?: Unknown

Causes understood?: Unknown

Causes reversible?: Unknown

Extreme fluctuations?: Unknown

#### Area of occupancy

Trend: Unknown

Causes ceased?: Unknown

Causes understood?: Unknown

Causes reversible?: Unknown

Extreme fluctuations?: Unknown

AOO (km2): Unknown

#### Locations

Number of locations: Unknown

Justification for number of locations: Data available (1 record) are not enough to estimate the number of locations.

Trend: Unknown

Extreme fluctuations?: Unknown

#### Population

Number of individuals: Unknown

Trend: Unknown

Causes ceased?: Unknown

Causes understood?: Unknown

Causes reversible?: Unknown

Extreme fluctuations?: Unknown

Population Information (Narrative): No estimates of population size exist.

#### Subpopulations

Number of subpopulations: Unknown

Trend: Unknown

Justification for trend: Data available (1 record) are not enough to estimate the number of subpopulations.

Extreme fluctuations?: Unknown

Severe fragmentation?: Unknown

#### Habitat

System: Terrestrial

Habitat specialist: Unknown

Habitat (narrative): This spider has currently been recorded only once in a shrubland (*Cystus* sp.).

Trend in extent, area or quality?: Unknown

##### Habitat

Habitat importance: Major Importance

Habitats: 3.8. Shrubland - Mediterranean-type Shrubby Vegetation

#### Habitat

Habitat importance: Major Importance

Habitats: 3.8. Shrubland - Mediterranean-type Shrubby Vegetation

#### Ecology

Size: 4.3 mm

Generation length (yr): 1

Dependency of single sp?: Unknown

Ecology and traits (narrative): An ant-eating species that produces no web and uses specialised predator behaviour and mimicry in order to capture its prey.

#### Threats

Justification for threats: The existence of threats is unknown for this species.

##### Threats

Threat type: Past

Threats: 12. Other options - Other threat

#### Threats

Threat type: Past

Threats: 12. Other options - Other threat

#### Conservation

Justification for conservation actions: The only locality, where this spider was collected, is in the Vale do Guadiana Natural Park and covered by the Natura 2000 network (PTZPE0047 and PTCON0036).

##### Conservation actions

Conservation action type: In Place

Conservation actions: 1.1. Land/water protection - Site/area protection1.2. Land/water protection - Resource & habitat protection

#### Conservation actions

Conservation action type: In Place

Conservation actions: 1.1. Land/water protection - Site/area protection1.2. Land/water protection - Resource & habitat protection

#### Other

##### Use and trade

Use type: International

##### Ecosystem services

Ecosystem service type: Very important

##### Research needed

Research needed: 1.2. Research - Population size, distribution & trends1.3. Research - Life history & ecology1.5. Research - Threats

Justification for research needed: Research is needed on basic information such as distribution, ecology, life cycle and possible threats throughout the range.

#### Use and trade

Use type: International

#### Ecosystem services

Ecosystem service type: Very important

#### Research needed

Research needed: 1.2. Research - Population size, distribution & trends1.3. Research - Life history & ecology1.5. Research - Threats

Justification for research needed: Research is needed on basic information such as distribution, ecology, life cycle and possible threats throughout the range.

#### Viability analysis

### Zodarion viduum

#### Species information

Scientific name: Zodarion viduum

Species authority: Denis, 1937

Kingdom: Animalia

Phylum: Arthropoda

Class: Arachnida

Order: Araneae

Family: Zodariidae

Region for assessment: Global

#### Geographic range

Biogeographic realm: Palearctic

Countries: Portugal

Map of records (Google Earth): Suppl. material 42

Basis of EOO and AOO: Species Distribution Model

Basis (narrative): Multiple collection sites are recorded for this species (15 records), mostly recent and in sand dunes (Bosmans 1994, Carvalho et al. 2011, Pekár et al. 2011). It was possible to perform species distribution modelling to predict its potential range with confidence limits. See Methods for details.

Min Elevation/Depth (m): 0

Max Elevation/Depth (m): 36

Range description: This spider is a common species in sand dunes in the coasts of central and northern Portugal. The species distribution modelling suggests that it could be widespread in dune areas from Porto to Leiria regions.

#### New occurrences

#### Extent of occurrence

EOO (km2): 1023 - 1700

Trend: Decline (inferred)

Justification for trend: The sand dunes, in which this species has been found, are delicate habitats that are threatened by habitat loss and possible increase in number of extreme weather events due to climate change.

Causes ceased?: No

Causes understood?: Yes

Causes reversible?: No

Extreme fluctuations?: No

#### Area of occupancy

Trend: Decline (inferred)

Justification for trend: The sand dunes in which this species has been found, are delicate habitats that are threatened by habitat loss and possible increase in number of extreme weather events due to climate change.

Causes ceased?: No

Causes understood?: Yes

Causes reversible?: No

Extreme fluctuations?: No

AOO (km2): 496 - 720

#### Locations

Number of locations: Unknown

Justification for number of locations: The number of threats needed to completely cover the species range is unknown but, in any case, larger than 10.

Trend: Decline (inferred)

Justification for trend: The sand dunes, in which this species has been found, are delicate habitats that are threatened by habitat loss and possible increase in number of extreme weather events due to climate change.

Extreme fluctuations?: No

#### Population

Number of individuals: Unknown

Trend: Decline (inferred)

Justification for trend: The sand dunes, in which this species has been found, are delicate habitats that are threatened by habitat loss and possible increase in number of extreme weather events due to climate change.

Basis for decline: (c) a decline in area of occupancy, extent of occurrence and/or quality of habitat

Causes ceased?: No

Causes understood?: Yes

Causes reversible?: No

Extreme fluctuations?: No

Population Information (Narrative): No estimates of population size exist.

#### Subpopulations

Number of subpopulations: Unknown

Trend: Decline (inferred)

Justification for trend: The sand dunes, in which this species has been found, are delicate habitats that are threatened by habitat loss and possible increase in number of extreme weather events due to climate change.

Extreme fluctuations?: No

Severe fragmentation?: Unknown

#### Habitat

System: Terrestrial

Habitat specialist: Yes

Habitat (narrative): Exclusive to coastal sand dunes in various Portuguese provinces.

Trend in extent, area or quality?: Decline (inferred)

Justification for trend: The sand dunes, in which this species has been found, are delicate habitats that are threatened by habitat loss and possible increase in number of extreme weather events due to climate change.

##### Habitat

Habitat importance: Major Importance

Habitats: 13.3. Marine Coastal/Supratidal - Coastal Sand Dunes

#### Habitat

Habitat importance: Major Importance

Habitats: 13.3. Marine Coastal/Supratidal - Coastal Sand Dunes

#### Ecology

Size: 2.5 - 3.5 mm

Generation length (yr): 1

Dependency of single sp?: Unknown

Ecology and traits (narrative): An ant-eating species that produces no web and uses specialised predator behaviour and mimicry in order to capture its prey.

#### Threats

Justification for threats: The sand dunes, in which this species has been found, are delicate habitats that are threatened by habitat loss and possible increase in number of extreme weather events due to climate change.

##### Threats

Threat type: Ongoing

Threats: 1.1. Residential & commercial development - Housing & urban areas1.2. Residential & commercial development - Commercial & industrial areas1.3. Residential & commercial development - Tourism & recreation areas11.4. Climate change & severe weather - Storms & flooding

#### Threats

Threat type: Ongoing

Threats: 1.1. Residential & commercial development - Housing & urban areas1.2. Residential & commercial development - Commercial & industrial areas1.3. Residential & commercial development - Tourism & recreation areas11.4. Climate change & severe weather - Storms & flooding

#### Conservation

Justification for conservation actions: This spider is found in beaches across north and central Portugal, being recorded once in the Dunas de São Jacinto Natural Reserve along an area also covered by the Natura 2000 network (PTCON0055; PTCON0061; PTZPE0004). Additionally, all beaches in Portugal are governed by the European Water Framework Directive (directive 2000/60/EC), being protected by means of land-use plans that preserve coastal ecosystems (decree-law Nº 130/2012).

##### Conservation actions

Conservation action type: In Place

Conservation actions: 1.1. Land/water protection - Site/area protection1.2. Land/water protection - Resource & habitat protection

#### Conservation actions

Conservation action type: In Place

Conservation actions: 1.1. Land/water protection - Site/area protection1.2. Land/water protection - Resource & habitat protection

#### Other

##### Use and trade

Use type: International

##### Ecosystem services

Ecosystem service type: Very important

##### Research needed

Research needed: 3.1. Monitoring - Population trends3.4. Monitoring - Habitat trends

Justification for research needed: Monitoring of population and habitat are important to confirm inferred trends.

#### Use and trade

Use type: International

#### Ecosystem services

Ecosystem service type: Very important

#### Research needed

Research needed: 3.1. Monitoring - Population trends3.4. Monitoring - Habitat trends

Justification for research needed: Monitoring of population and habitat are important to confirm inferred trends.

#### Viability analysis

## Discussion

**Figure 1.**
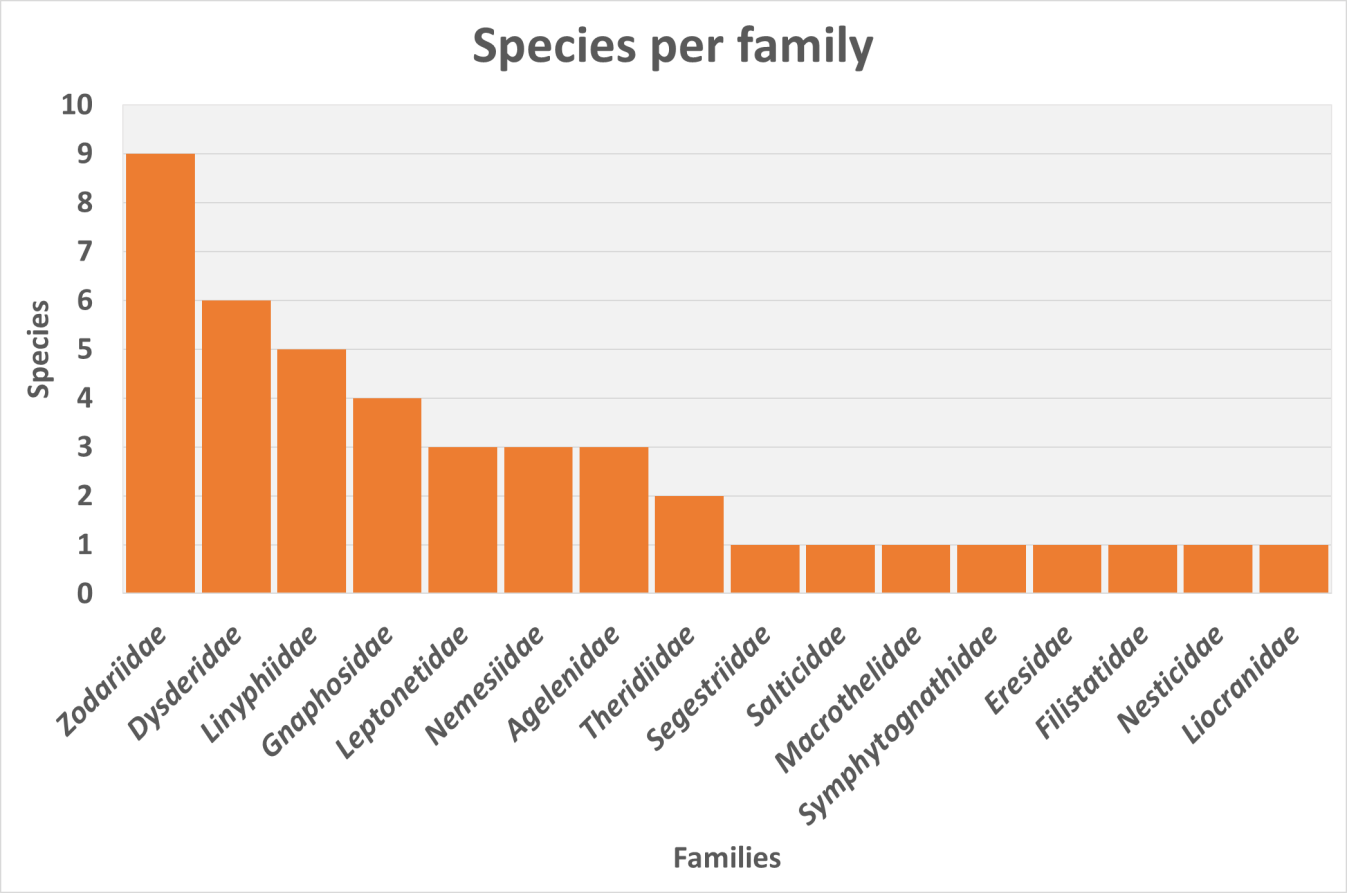
Number of assessed species per family.

The 43 assessed species belong to 15 families, the richest being Zodariidae (9 species, 21.4%), Dysderidae (6, 14.2%), Linyphiidae (5, 12.0%), Gnaphosidae (4, 9.5%), Agelenidae, Leptonetidae and Nemesiidae (3 species each, 7.1%) (Fig. 1). This is consistent with the general patterns for the Iberian Peninsula and the Mediterranean as a whole, with Zodariidae, Dysderidae and Nemesiidae, in particular, having large proportions of endemic species (Branco et al. 2019).

**Figure 2.**
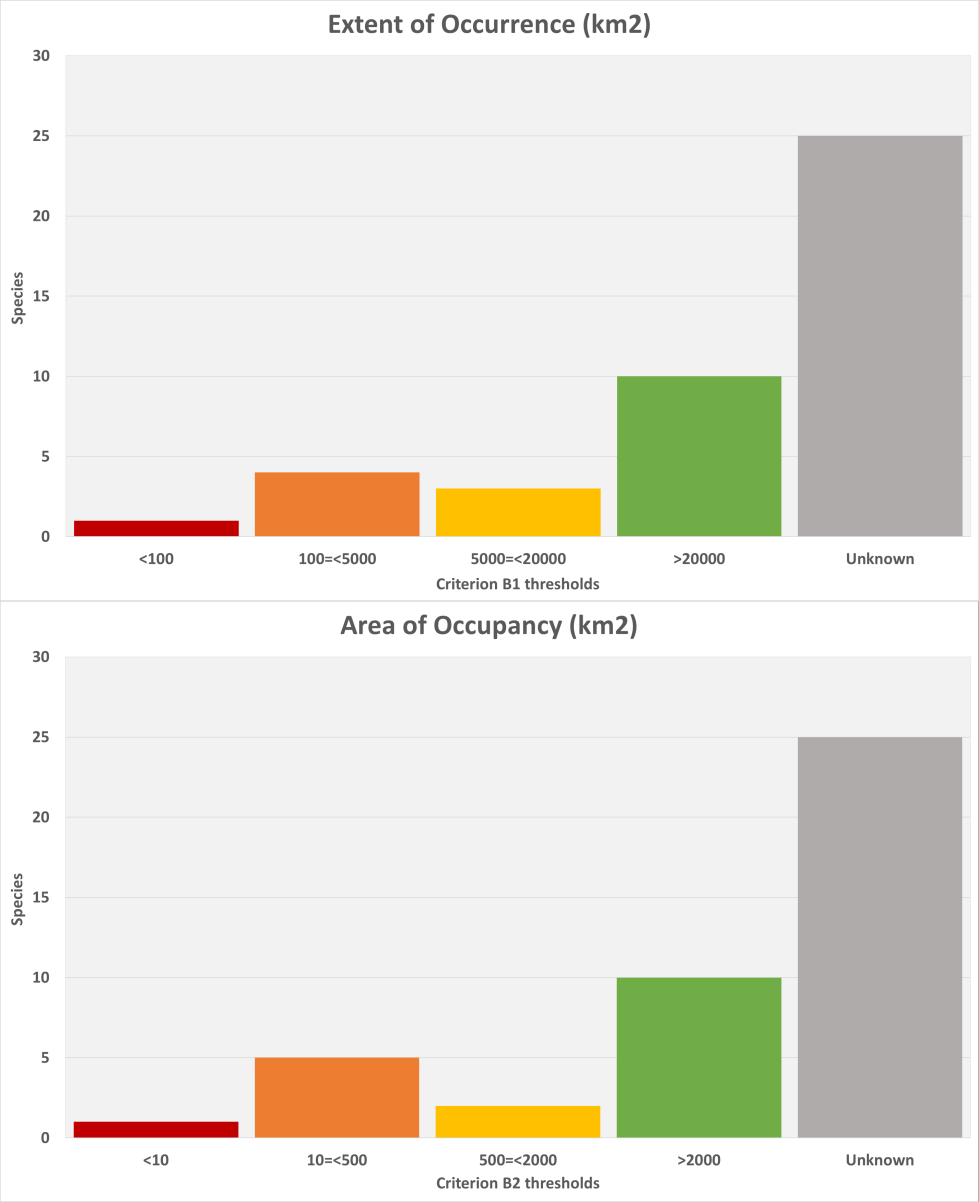
Extent of Occurrence (EOO) and Area of Occupancy (AOO) amongt all species.

From the 43 species evaluated, only 18 had enough data to allow their EOO and AOO to be quantified. Of these, we modelled the distribution of 14 epigean species, eight of which were found to be widespread (both EOO > 20 000 km^2^ and AOO > 2000 km^2^). The remaining six fulfilled at least one of the criteria for threatened species. Four species are troglobiont, whose distribution was assumed to be well known and therefore assessed using the known occurrence points: *Anapistula ataecina*, *Domitius lusitanicus*, *Harpactea stalitoides*, *Teloleptoneta synthetica*. As expected from species with such life history, all of them fulfil the EOO and AOO thresholds for threatened species. The remaining 25 Portuguese endemics (59.5%) had no reliable information on their range (Fig. 2). Even though the last decade has seen a large increase in the knowledge of Iberian spiders (Branco et al. 2019), the available information is still far from satisfactory and it is not possible to assess the majority of species for their risk of extinction.

**Figure 3.**
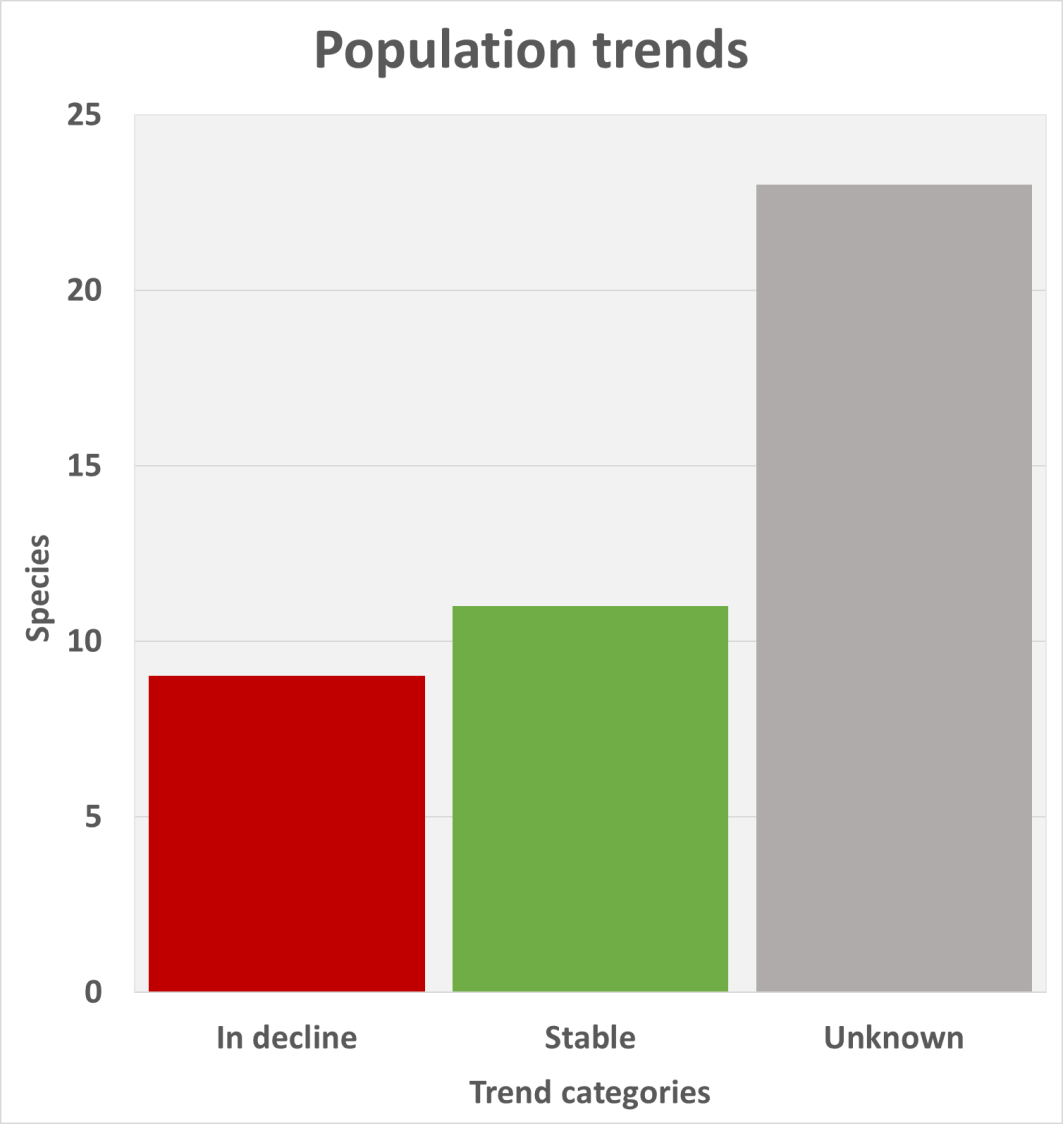
Population trends amongt all species.

**Figure 4.**
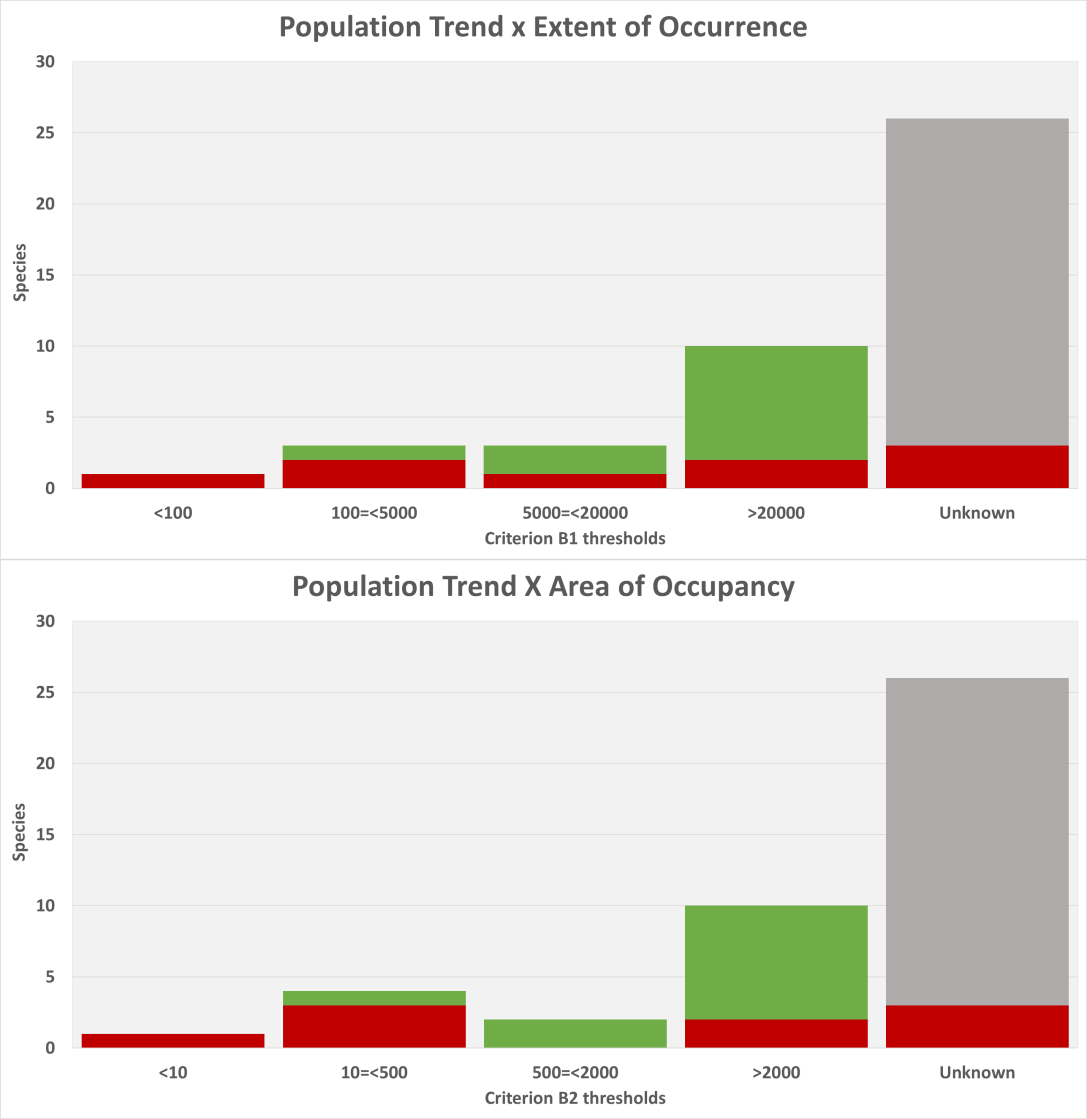
Range (EOO and AOO) categories of all species, arranged according to their population trends. Species classified as "In decline" in red, species classified as "Stable" in green, species classified as "Unknown" in grey.

Population trends equally suffer greatly from lack of information. From the 43 species assessed, only nine are estimated to be in decline, 11 stable, with the majority of species having no information on trends (23 species, Fig. 3). Although there is a larger number of species with a restricted EOO amongst those declining than those with stable population numbers, this difference is not very noticeable. It is, however, observable that stable species are far more likely to have larger EOO (>20000 km2). A difference between both trend groups is much more observable when comparing AOO values, with species occupying less than 500 km2 being more commonly declining (Fig. 4).

**Figure 5.**
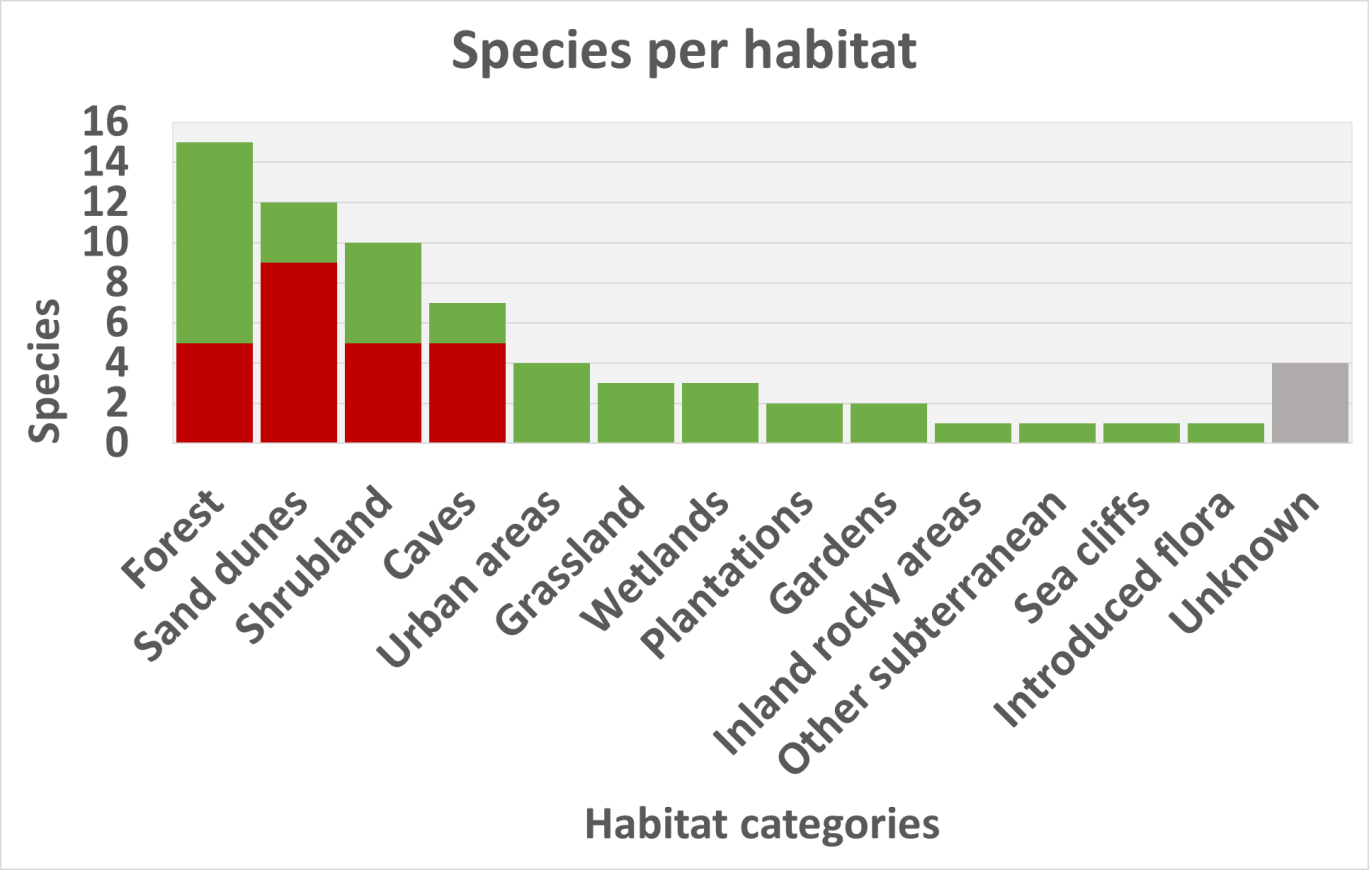
Habitat categories amongst all 42 species assessed. Species exclusive to each habitat in red.

Portuguese endemics occupy a variety of habitats. Forest areas (15 species), sand dunes (12 species), shrublands (10) and caves (6) host the majority of species (Fig. 5). All four most represented habitats are inhabited by a number of species exclusive to them. From all four, sand dunes have the largest count of exclusive species (9) and forests have the largest count of non-exclusive species (10).

Forests vary considerably in their composition of dominant trees, according to the biogeographic region in question and are frequently both natural and semi-natural systems. Besides the threat of man-made wildfires, increasingly important during this era of accelerated climate change, endemic Portuguese forests and shrublands face an additional, ongoing threat to their extension: the mass production of *Eucalyptus globosum* (Labill). Often poorly managed plantations may not only be exacerbating the occurrence of wildfires (Fernandes et al. 2011), but also seem to have direct consequences in invertebrate diversity (Cammell et al. 1996, Zahn et al. 2009, Corcuera et al. 2015) that should be addressed.

Species occurring on sand dunes are significantly represented, with a majority being exclusive to this habitat. However, given most of these species have no reliable information on their range, future research may prove this to be at least partly biased, either due to recent work targeting this habitat (Carvalho et al. 2011) or many foreign scholars having collected preferentially in this habitat type while on vacation. Regardless, the importance of protecting sand dune habitats in Portugal is indisputable and these areas must continue to be considered important conservation areas in both national environmental legislation as well as local legislation, such as municipal land use plans (PDMs). As Portugal expands to accommodate its growing tourism industry, efforts must be made to direct this growth inwards instead of continuously stressing a coastal region with an outstanding natural value (Deharveng et al. 2000, Cuttelod et al. 2008).

When considering *Anapistula ataecina* (Cardoso 2010), troglobiont species represent 9.5% of endemic species. Cave systems are often home to species with very restricted distributions and these habitats are more sensitive to perturbation than most other habitat types. Current limestone quarries, in areas like Serra da Arrábida, pose a serious threat to several species. A change in the opening of a cave system can modify its temperature, airflow, humidity and completely wipe out a subpopulation in a way that is irreversible without structural work being performed post-occupation (Cardoso and Scharff 2009). One should note, though, that often conservation priorities are in conflict and habitat recovery might imperil some species. Gruta do Zambujal is such an example: structural changes to the cave's entrance have led to increased airflow and temperature changes inside, reducing endemic spider fauna while simultaneously allowing the colonisation by protected bat species.

**Figure 6.**
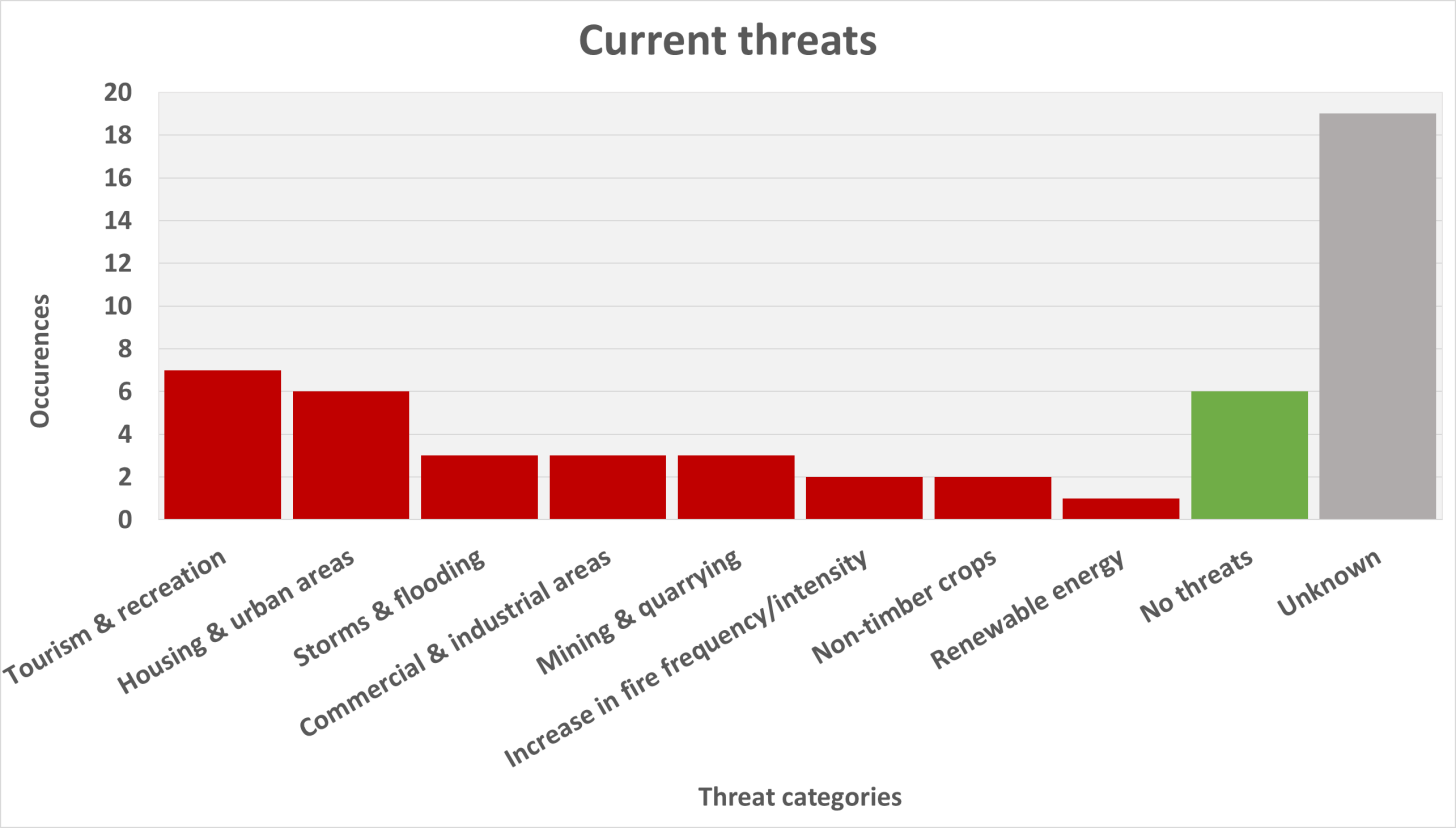
Threat categories amongst all species.

The threats to Portuguese endemics reflect the diversity of habitats they occupy. Urbanisation and climate change seem to be the most important threats to these species (Fig. 6), although other factors are also important and represented across the data.

A considerable proportion of the currently known Portuguese endemic richness can be found in national protected areas, with special focus on the natural park system. Out of 234 records used, 112 were located inside protected areas (47.8%). The Serras de Aire e Candeeiros Natural Park has the largest number of records within it (31), followed by Douro Internacional Natural Park (16), Vale do Guadiana Natural Park (12), Sudoeste Alentejano e Costa Vicentina Natural Park (11) and Arrábida Natural Park (10). Of all species considered here, 29 (69.0%) possess at least one record in an area belonging to the National Protected Area Network (RNAP). Although this might seem encouraging and somehow indicating that protected areas are well located, most of these numbers might be due to the fact that these specific protected areas in Portugal were relatively well sampled during the recent decades (Cardoso 2004, Cardoso et al. 2007, Carvalho et al. 2011).

Presence in the Natura 2000 network is also common. The (PTCON0015) Serras de Aire e Candeeiros site has the most records (29), followed by (PTZPE0047) Vale do Guadiana (12), the (PTCON0012/PTZPE0015) Costa Sudoeste and (PTCON0010) Arrábida / Espichel (10) sites, (PTCON0061/PTZPE0004) Ria de Aveiro (8) and the (PTCON0049) Barrocal and (PTCON0048) Serra de Montejunto (7) sites. Out of 234 records, 169 are located inside areas of the Natura 2000 network (72.2%). Of all species considered in this study, 31 (73.8%) possess at least one record in an area belonging to the Natura 2000 network.

Finally, regarding conservation measures needed to protect endemic spider species, site/area protection and/or resource & habitat protection were invariably found to be most important. This is especially critical since no spider endemic to mainland Portugal is protected by law, national or international and hence their protection has never been considered in conservation plans and only when they coincide with protected areas or other protected species, is it possible to somehow safeguard them. As mentioned previously, the only spider protected in the country is not endemic, *Macrothele calpeiana*and should be assessed as Least Concern, given its wide range and high adaptability to different habitat types.

Discussion

## Supplementary Material

95378C4B-9B25-5F6E-B8BE-BF34469728E2 10.3897/BDJ.7.e39315.suppl1 Supplementary material 1 Distribution of Eratigena barrientosi (Bolzern, Crespo & Cardoso, 2009) Data type: Distribution
File: oo_312917.kml https://binary.pensoft.net/file/312917 Branco, V.; Cardoso, P.

C41709B8-1482-513B-B5A1-365638B55FB6 10.3897/BDJ.7.e39315.suppl2 Supplementary material 2 Distribution of Eratigena incognita (Bolzern, Crespo & Cardoso, 2009) Data type: Distribution
File: oo_277476.kml https://binary.pensoft.net/file/277476 Branco, V.; Cardoso, P.

7228EA7B-083A-5EB2-8BF3-85F3A2DED4A5 10.3897/BDJ.7.e39315.suppl3 Supplementary material 3 Distribution of Malthonica oceanica (Barrientos & Cardoso, 2007) Data type: Distribution
File: oo_277482.kml https://binary.pensoft.net/file/277482 Branco, V.; Cardoso, P.

D91F7B67-1488-56A1-8A4B-922302204744 10.3897/BDJ.7.e39315.suppl4 Supplementary material 4 Distribution of Dysdera alentejana (Ferrández, 1996) Data type: Distribution
File: oo_277790.kml https://binary.pensoft.net/file/277790 Branco, V.; Cardoso, P.

538E9CCB-E572-591F-A5E5-6DFF34591259 10.3897/BDJ.7.e39315.suppl5 Supplementary material 5 Distribution of Harpactea algarvensis (Ferrández, 1990) Data type: Distribution
File: oo_277792.kml https://binary.pensoft.net/file/277792 Branco, V.; Cardoso, P.

005AA85B-47F8-5B77-B7A9-3FAA90F80823 10.3897/BDJ.7.e39315.suppl6 Supplementary material 6 Distribution of Harpactea magnibulbi (Machado & Ferrández, 1991) Data type: Distribution
File: oo_312913.kml https://binary.pensoft.net/file/312913 Branco, V.; Cardoso, P.

51561791-9448-5931-915A-782636037BFB 10.3897/BDJ.7.e39315.suppl7 Supplementary material 7 Distribution of Harpactea proxima (Ferrández, 1990) Data type: Distribution
File: oo_278248.kml https://binary.pensoft.net/file/278248 Branco, V.; Cardoso, P.

06E982F5-AACC-532C-916D-97C84A4A7E1A 10.3897/BDJ.7.e39315.suppl8 Supplementary material 8 Distribution of Harpactea stalitoides (Ribera, 1993) Data type: Distribution
File: oo_277868.kml https://binary.pensoft.net/file/277868 Branco, V.; Cardoso, P.

213D6113-5870-519B-A4FA-D374E1711B40 10.3897/BDJ.7.e39315.suppl9 Supplementary material 9 Distribution of Harpactea subiasi (Ferrández, 1990) Data type: Distribution
File: oo_277869.kml https://binary.pensoft.net/file/277869 Branco, V.; Cardoso, P.

A87412AE-1309-55E1-9646-CA278C659E78 10.3897/BDJ.7.e39315.suppl10 Supplementary material 10 Distribution of Adonea algarvensis (Wunderlich, 2017) Data type: Distribution
File: oo_277876.kml https://binary.pensoft.net/file/277876 Branco, V.; Cardoso, P.

40068570-7A2C-5AA9-ADEB-B7D28B8EDF2B 10.3897/BDJ.7.e39315.suppl11 Supplementary material 11 Distribution of Filistata pygmaea (Zonstein, Marusik & Grabolle, 2018) Data type: Distribution
File: oo_277896.kml https://binary.pensoft.net/file/277896 Branco, V.; Cardoso, P.

8E2999AB-8C37-54BC-B847-F18482233BE4 10.3897/BDJ.7.e39315.suppl12 Supplementary material 12 Distribution of Scotophaeus dolanskyi (Lissner, 2017) Data type: Distribution
File: oo_277900.kml https://binary.pensoft.net/file/277900 Branco, V.; Cardoso, P.

5573F367-4E91-5FCC-B8D3-AEAED36E166A 10.3897/BDJ.7.e39315.suppl13 Supplementary material 13 Distribution of Scotophaeus nanoides (Wunderlich, 2011) Data type: Distribution
File: oo_309143.kml https://binary.pensoft.net/file/309143 Branco, V.; Cardoso, P.

8ECBC6E8-F3CE-501F-842E-055A5D86481B 10.3897/BDJ.7.e39315.suppl14 Supplementary material 14 Distribution of Trachyzelotes minutus (Crespo, 2010) Data type: Distribution
File: oo_277903.kml https://binary.pensoft.net/file/277903 Branco, V.; Cardoso, P.

36393888-7BD7-5A2E-90EC-D847B097C81F 10.3897/BDJ.7.e39315.suppl15 Supplementary material 15 Distribution of Zelotes fuzeta (Wunderlich, 2011) Data type: Distribution
File: oo_277906.kml https://binary.pensoft.net/file/277906 Branco, V.; Cardoso, P.

AC955E84-DB47-5457-B101-421564ED6922 10.3897/BDJ.7.e39315.suppl16 Supplementary material 16 Distribution of Macrothele calpeiana (Walckenaer, 1805) Data type: Distribution
File: oo_302701.kml https://binary.pensoft.net/file/302701 Branco, V.; Cardoso, P.

A3AB268E-A9FC-57AF-BC84-EC1F6895E5B4 10.3897/BDJ.7.e39315.suppl17 Supplementary material 17 Distribution of Leptoneta berlandi (Machado & Ribera, 1986) Data type: Distribution
File: oo_277910.kml https://binary.pensoft.net/file/277910 Branco, V.; Cardoso, P.

8E415503-D7D2-5B5F-AEEE-A1E8AE084ED2 10.3897/BDJ.7.e39315.suppl18 Supplementary material 18 Distribution of Leptoneta conimbricensis (Machado & Ribera, 1986) Data type: Distribution
File: oo_277913.kml https://binary.pensoft.net/file/277913 Branco, V.; Cardoso, P.

963CBBE0-F13E-51D1-BAAD-7B25FFBA732D 10.3897/BDJ.7.e39315.suppl19 Supplementary material 19 Distribution of Teloleptoneta synthetica (Machado, 1951) Data type: Distribution
File: oo_277951.kml https://binary.pensoft.net/file/277951 Branco, V.; Cardoso, P.

BB96B9E3-475A-52FA-B492-B72A3D9A96F3 10.3897/BDJ.7.e39315.suppl20 Supplementary material 20 Distribution of Bordea berlandi (Fage, 1931) Data type: Distribution
File: oo_277952.kml https://binary.pensoft.net/file/277952 Branco, V.; Cardoso, P.

67011C81-6D2E-535C-81BB-58384A335FD4 10.3897/BDJ.7.e39315.suppl21 Supplementary material 21 Distribution of Labulla machadoi (Hormiga & Scharff, 2005) Data type: Distribution
File: oo_277963.kml https://binary.pensoft.net/file/277963 Branco, V.; Cardoso, P.

788D9087-9A06-5033-BA8F-DF1E534C081A 10.3897/BDJ.7.e39315.suppl22 Supplementary material 22 Distribution of Maso douro (Bosmans & Cardoso, 2010) Data type: Distribution
File: oo_278007.kml https://binary.pensoft.net/file/278007 Branco, V.; Cardoso, P.

15D39E5B-C5F9-5379-95CA-B2B272A1C929 10.3897/BDJ.7.e39315.suppl23 Supplementary material 23 Distribution of Parapelecopsis conimbricensis (Bosmans & Crespo, 2010) Data type: Distribution
File: oo_278013.kml https://binary.pensoft.net/file/278013 Branco, V.; Cardoso, P.

CB268B1A-06E1-50B0-AA8A-AD4FCFB5B6EF 10.3897/BDJ.7.e39315.suppl24 Supplementary material 24 Distribution of Trichoncus similipes (Denis, 1965) Data type: Distribution
File: oo_278016.kml https://binary.pensoft.net/file/278016 Branco, V.; Cardoso, P.

71E33B44-4AC9-5971-BD72-1AE856F5D6C7 10.3897/BDJ.7.e39315.suppl25 Supplementary material 25 Distribution of Apostenus crespoi (Lissner, 2017) Data type: Distribution
File: oo_278020.kml https://binary.pensoft.net/file/278020 Branco, V.; Cardoso, P.

6E59FD14-D291-552B-9815-322F0A6B8E40 10.3897/BDJ.7.e39315.suppl26 Supplementary material 26 Distribution of Nemesia bacelarae (Decae, Cardoso & Selden, 2007) Data type: Distribution
File: oo_278025.kml https://binary.pensoft.net/file/278025 Branco, V.; Cardoso, P.

9B20C386-41BF-5D73-8CDA-C175F2C0818C 10.3897/BDJ.7.e39315.suppl27 Supplementary material 27 Distribution of Nemesia berlandi (Frade & Bacelar, 1931) Data type: Distribution
File: oo_278041.kml https://binary.pensoft.net/file/278041 Branco, V.; Cardoso, P.

BD8D3B01-8434-565C-A7CE-601B24CF3AD6 10.3897/BDJ.7.e39315.suppl28 Supplementary material 28 Distribution of Nemesia fagei (Frade & Bacelar, 1931) Data type: Distribution
File: oo_278042.kml https://binary.pensoft.net/file/278042 Branco, V.; Cardoso, P.

FBB78BFC-468D-549A-ADD7-2BA68352AC85 10.3897/BDJ.7.e39315.suppl29 Supplementary material 29 Distribution of Domitius lusitanicus (Fage, 1931) Data type: Distribution
File: oo_278059.kml https://binary.pensoft.net/file/278059 Branco, V.; Cardoso, P.

6BF12287-0835-5F2F-B406-08BD585D3188 10.3897/BDJ.7.e39315.suppl30 Supplementary material 30 Distribution of Pseudomogrus algarvensis (Logunov & Marusik, 2003) Data type: Distribution
File: oo_278171.kml https://binary.pensoft.net/file/278171 Branco, V.; Cardoso, P.

1F87D13D-4A1D-5BBD-AC9E-6D8EE00485C1 10.3897/BDJ.7.e39315.suppl31 Supplementary material 31 Distribution of Ariadna inops (Wunderlich, 2011) Data type: Distribution
File: oo_278174.kml https://binary.pensoft.net/file/278174 Branco, V.; Cardoso, P.

E0C818ED-1DBD-5075-ABAC-B21BC21F3CC2 10.3897/BDJ.7.e39315.suppl32 Supplementary material 32 Distribution of Lasaeola algarvensis (Wunderlich, 2011) Data type: Distribution
File: oo_278175.kml https://binary.pensoft.net/file/278175 Branco, V.; Cardoso, P.

DAD68BFE-01EF-5229-804A-A2A921E5EE44 10.3897/BDJ.7.e39315.suppl33 Supplementary material 33 Distribution of Theridion bernardi (Lecigne, 2017) Data type: Distribution
File: oo_278189.kml https://binary.pensoft.net/file/278189 Branco, V.; Cardoso, P.

75CB3362-032F-5C2B-BD25-6A4043D12C12 10.3897/BDJ.7.e39315.suppl34 Supplementary material 34 Distribution of Amphiledorus ungoliantae (Pekár & Cardoso, 2005) Data type: Distribution
File: oo_278193.kml https://binary.pensoft.net/file/278193 Branco, V.; Cardoso, P.

4FE47533-EFE9-59F9-9DF7-F27A20D14A61 10.3897/BDJ.7.e39315.suppl35 Supplementary material 35 Distribution of Zodarion alentejanum (Pekár & Carvalho, 2011) Data type: Distribution
File: oo_325177.kml https://binary.pensoft.net/file/325177 Branco, V.; Cardoso, P.

44697375-3E45-5388-A11C-09910A1C64E7 10.3897/BDJ.7.e39315.suppl36 Supplementary material 36 Distribution of Zodarion algarvense (Bosmans, 1994) Data type: Distribution
File: oo_325176.kml https://binary.pensoft.net/file/325176 Branco, V.; Cardoso, P.

1F96B941-125D-5491-AA0D-45B269A5B3C8 10.3897/BDJ.7.e39315.suppl37 Supplementary material 37 Distribution of Zodarion bacelarae (Pekár, 2003) Data type: Distribution
File: oo_278201.kml https://binary.pensoft.net/file/278201 Branco, V.; Cardoso, P.

FE9D553F-8B35-5FC7-81C0-EE96E00F4A4E 10.3897/BDJ.7.e39315.suppl38 Supplementary material 38 Distribution of Zodarion bosmansi (Pekár & Cardoso, 2005) Data type: Distribution
File: oo_278211.kml https://binary.pensoft.net/file/278211 Branco, V.; Cardoso, P.

66B0CB66-F5CF-590A-B144-167CAF3CFC09 10.3897/BDJ.7.e39315.suppl39 Supplementary material 39 Distribution of Zodarion costapratae (Pekár, 2011) Data type: Distribution
File: oo_278222.kml https://binary.pensoft.net/file/278222 Branco, V.; Cardoso, P.

2589ED5E-A3D1-589D-AEA0-F8279ADB9216 10.3897/BDJ.7.e39315.suppl40 Supplementary material 40 Distribution of Zodarion duriense (Cardoso, 2003) Data type: Distribution
File: oo_278226.kml https://binary.pensoft.net/file/278226 Branco, V.; Cardoso, P.

DED644EB-CC5F-5013-9B64-619B9FCC0233 10.3897/BDJ.7.e39315.suppl41 Supplementary material 41 Distribution of Zodarion guadianense (Cardoso, 2003) Data type: Distribution
File: oo_278228.kml https://binary.pensoft.net/file/278228 Branco, V.; Cardoso, P.

4D3F82C5-DB42-5F38-976A-D5CDD9A9F355 10.3897/BDJ.7.e39315.suppl42 Supplementary material 42 Distribution of Zodarion viduum (Denis, 1937) Data type: Distribution
File: oo_325307.kml https://binary.pensoft.net/file/325307 Branco, V.; Cardoso, P.

## References

[B4996742] Bacelar A. (1937). Trap-door spiders from Algarve (South of Portugal). Comptes Rendus du XII Conges International de Zoologie.

[B4996682] Barrientos J., Cardoso P. (2007). The genus *Malthonica* Simon, 1898 in the Iberian Peninsula (Araneae, Agelenidae). Zootaxa.

[B4998299] Bellvert A., Arnedo M. (2016). Threatened or threatening? Evidence for independent introductions of *Macrothele
calpeiana* (Araneae: Hexathelidae) and first observation of reproduction outside its natural distribution range. Arachnology.

[B4996807] Benhadi-Marin Jacinto, Pereira Jose A., Barrientos Jose A., Sousa Jose P., Santos Sonia A. P. (2018). Stones on the ground in olive groves promote the presence of spiders (Araneae). European Journal of Entomology.

[B4997155] Bolzern A., Crespo L C, Cardoso P. (2009). Two new *Tegenaria* species (Araneae: Agelenidae) from Portugal. Zootaxa.

[B4996712] Bosmans R. (1994). Revision of the genus *Zodarion* Walckenaer, 1833 in the Iberian peninsula and Balearic islands (Araneae, Zodariidae). Eos.

[B4996436] Bosmans ROBERT, Cardoso PEDRO, Crespo LUIS C (2010). A review of the linyphiid spiders of Portugal, with the description of six new species (Araneae: Linyphiidae). Zootaxa.

[B5008519] Branco V V, Morano E, Cardoso P (2019). An update to the Iberian spider checklist (Araneae). Zootaxa.

[B5009496] Breiner Frank T., Nobis Michael P., Bergamini Ariel, Guisan Antoine (2018). Optimizing ensembles of small models for predicting the distribution of species with few occurrences. Methods in Ecology and Evolution.

[B5309777] Cammell M. E., Way M. J., Paiva M. R. (1996). Diversity and structure of ant communities associated with oak, pine, eucalyptus and arable habitats in Portugal. Insectes Sociaux.

[B4996447] Cardoso Pedro (2004). The use of arachnids (Class Arachnida) in biodiversity evaluation and monitoring of natural areas.

[B5309314] Cardoso Pedro, Silva Israel, De Oliveira Nuno G, Serrano Artur R M (2007). Seasonality of spiders (Araneae) in Mediterranean ecosystems and its implications in the optimum sampling period. Ecological Entomology.

[B4998320] Cardoso P. (2008). Biodiversity and conservation of Iberian spiders: past, present and future. Boletin de la Sociedad Entomologica Aragonesa.

[B4996963] Cardoso Pedro, Gaspar Clara, Pereira Luis C., Silva Israel, Henriques Sérgio S., da Silva Ricardo R., Sousa Pedro (2008). Assessing spider species richness and composition in Mediterranean cork oak forests. Acta Oecologica.

[B4997029] Cardoso P., Scharff N., Gasper C., Henriques S., Carvalho Rui, Castro P., Schmidt J., Silva I., Szüts T., Castro A., Crespo L C (2008). Rapid biodiversity assessment of spiders (Araneae) using semi-quantitative sampling: a case study in a Mediterranean forest. Insect Conservation and Diversity.

[B5309224] Cardoso Pedro, Scharff Nikolaj (2009). First record of the spider family Symphytognathidae in Europe and description of *Anapistula
ataecina* sp. n. (Araneae). Zootaxa.

[B4996929] Cardoso P., Henriques S. S., Gaspar C., Crespo L. C., Carvalho R., Schmidt J. B., Sousa P., Szüts T. (2009). Species richness and composition assessment of spiders in a Mediterranean scrubland. Journal of Insect Conservation.

[B5008892] Cardoso P *Anapistula
ataecina*. The IUCN Red List of Threatened Species 2010: e.T176265A7207415.

[B5008873] Cardoso Pedro (2017). red - an R package to facilitate species red list assessments according to the IUCN criteria. Biodiversity Data Journal.

[B4996641] Carvalho José C., Cardoso Pedro, Crespo Luís C., Henriques Sérgio, Carvalho Rui, Gomes Pedro (2011). Biogeographic patterns of spiders in coastal dunes along a gradient of mediterraneity. Biodiversity and Conservation.

[B5309787] Corcuera Pablo, Valverde Pedro Luis, Jiménez María Luisa, Ponce-Mendoza Alejandro, De la Rosa Gabriela, Nieto Gisela (2015). Ground spider guilds and functional diversity in native pine woodlands and eucalyptus plantations. Environmental Entomology.

[B4996953] Crespo L C (2008). Contribution to the knowledge of the Portuguese spider (Arachnida: Araneae) fauna: seven new additions to the Portuguese checklist. Boletín de la Sociedad Entomológica Aragonesa.

[B4996496] Crespo L C, Cardoso P., Carvalho R., Henriques S., Rufino A. (2009). Spiders (Arachnida, Araneae) from the Paul de Arzila Natural Reserve (Portugal). Boletín de la Sociedad Entomológica Aragonesa.

[B4997165] Crespo L C, Mendes S. (2010). Trachyzelotes
minutus, a new zelotine ground spider (Araneae: Gnaphosidae: Zavattaricinae) species from southern Portugal. Journal of Arachnology.

[B4996659] Crespo L C, Rufino A., Videira S., Cardoso P. (2010). Trabalho de campo efectuado no Parque Natural da Serra de São Mamede. Maio 2010. Report.

[B5309363] Cuttelod A., García N., Abdul Malak D., Temple H., Katariya V., Hilton-Taylor C, Stuart SN (2008). The Mediterranean: a biodiversityhotspot under threat. The 2008 review of the IUCN Red List of threatened species..

[B4996507] Decae A. E., Cardoso P., Selden P. A. (2007). Taxonomic review of the Portuguese Nemesiidae (Araneae, Mygalomorphae). Revista Ibérica de Aracnologia.

[B5309521] Deharveng L., Dalens H., Drugmand D., Simon-Benito J. C., Da Gama M. M., Sousa P., Gers C., Bedos A. (2000). Endemism mapping and biodiversity conservation in western Europe: An arthropod perspective. Belgian Journal of Entomology.

[B4997760] Denis J. (1965). Notes sur les Erigonides. XXVIII. Le genre *Triehoneus* (Araneae). Annales de la Société Entomologique de France.

[B5008728] Agency European Environment Biogeographical regions in Europe. https://www.eea.europa.eu/ds_resolveuid/9AFE2A4D-ADF9-45CD-A5A9-26E34640D494.

[B4998520] Fage L., Duthiers H L (1931). Araneae, 5e série, précédée d'un essai sur l'évolution souterraine et son déterminisme. Biospeologica, LV.

[B5309799] Fernandes P., Loureiro C., Palheiro P., Vale-Gonçalves HF., Fernandes M., Cruz M. (2011). Fuels and fire hazard in blue gum (*Eucalyptus
globulus*) stands in Portugal. Boletín del CIDEU.

[B4997019] Ferrández M. (1990). Notas sobre los disderidos ibericos VII. Descripcion de tres nuevas especies de *Harpactea* Bristowe, 1939 (Araneae: Dysderidae) del Sur de Portugal. Boletin de la Real Sociedad Española de Historia Natural (Seccion de Biologia).

[B4996919] Ferrández M. (1996). Notas sobre los disderidos ibericos VIII. Nuevas especies del genero *Dysdera* Latreille, 1804 (Araneae, Dysderidae). Boletin de la Real Sociedad Española de Historia Natural (Seccion de Biologia).

[B5008911] Fick S E, Hijmans R J (2017). Worldclim 2: New 1-km spatial resolution climate surfaces for global land areas. International Journal of Climatology.

[B4997871] Frade M F, Bacelar A. (1931). Révision des Nemesia de la faune ibérique et description d'espèces nouvelles de ce genre. Bulletin de Museum d'Histoire Naturelle de Paris.

[B5008844] Freire S, Santos T, Tenedório J (2009). Recent urbanization and land use/land cover change in Portugal - the influence of coastline and coastal urban centers. Journal of Coastal Research.

[B4998289] González-Moliné A. (2018). Catalogue of the spiders (Araneae) of Huelva Province, Iberian Peninsula. Revista Ibérica de Aracnología.

[B5008434] Helsdingen P J, Decae A E (1992). Ecology, distribution and vulnerability of *Macrothele
calpeiana* (Walckenaer) (Araneae, Hexathelidae).. Tijdschrift Voor Entomologie.

[B4997046] Hormiga G., Scharff N. (2005). Monophyly and phylogenetic placement of the spider genus *Labulla* Simon, 1884 (Araneae, Linyphiidae) and description of the new genus *Pecado*.. Zoological Journal of the Linnean Society.

[B5008854] IPCC (2014). Climate Change 2014: Synthesis Report. Contribuition of Working Groups I, II and III to the Fifth Assessment Report of the Intergovernmental Panel on Climate Change.

[B5297365] Jocqué R., Bosmans R. (2001). A revision of the genus *Selamia* with the description of *Amphiledorus* gen. n. (Araneae, Zodariidae).. Bulletin de l'Institut Royal des Sciences Naturelles de Belgique, Entomologie.

[B4997814] Lecigne S. (2017). Contribution to the spider (Araneae) survey of the Algarve (Portugal). Description of *Theridion
bernardi* n. sp. (Araneae: Theridiidae) and rediscovery of *Ozyptila
perplexa* Simon, 1875 (Araneae: Thomisidae). Revista Ibérica de Aracnología.

[B4998279] Lissner Jørgen (2017). A contribution to the knowledge of *Rhomphaea* L. Koch, 1872 (Araneae: Theridiidae) in the Mediterranean and Macaronesian Regions. Arachnology.

[B5169143] Lissner Jørgen (2017). New records of spiders (Araneae) from Portugal. Arachnologische Mitteilungen.

[B4998225] Logunov D., Marusik Y. (2003). A revision of the genus *Yllenus* Simon, 1868 (Arachnida, Araneae, Salticidae).

[B5009506] Lomba A., Pellissier L., Randin C., Vicente J., Moreira F., Honrado J., Guisan A. (2010). Overcoming the rare species modelling paradox: A novel hierarchical framework applied to an Iberian endemic plant. Biological Conservation.

[B4997780] Machado A. (1937). Aranhas novas para a fauna Portuguesa. Memorias e Estudos do Museu de Zoologia da Universidade de Coimbra.

[B4997096] Machado A. (1941). Araignees nouvelles pour la faune portugaise (II). Memorias e Estudos do Museu de Zoologia da Universidade de Coimbra.

[B4996456] Machado A. (1942). A coleçao de aranhas cavernícolas do Museo Nacional de Ciências Naturais de Madrid. Anales de la Asociacion Espanola para el Progreso de las Ciencias.

[B4996943] Machado A. (1945). A propos de l'appareil respiratoire des "Leptonetidae" (Araneae). Publicações do Museu de Zoologia do Porto.

[B4997116] Machado A., Ribera C. (1986). Araneidos cavernícolas de Portugal: familia Leptonetidae (Araneae). Actas X Congreso Internacional de Aracnologia.

[B4997076] Machado A., Ferrández M. (1991). *Harpactea
magnibulbi* n. sp. un nuevo disderido (Araneae, Dysderidae), del sur de Portugal. Boletin de la Real Sociedad Espanola de Historia Natural (Seccion de Biologia).

[B4996722] Main H. (1949). Some trap-door spiders from Algarve (S. Portugal). Proceedings of the South London Entomological & Natural History Society.

[B4998477] Morano E., Branco V V, Carillo J., Cardoso P. Iberian Spider Catalogue (v4.0). http://www.biodiversityresearch.org/iberia.

[B5008747] Nunes Adélia N., de Almeida António C., Coelho Celeste O. A. (2011). Impacts of land use and cover type on runoff and soil erosion in a marginal area of Portugal. Applied Geography.

[B4996828] Pekár S., Cardoso P., Meierrose C. (2003). Additions to the knowledge of Portuguese zodariid spiders (Araneae: Zodariidae). Bulletin of the British Arachnological Society.

[B4996702] Pekár Stano, Cardoso Pedro (2005). Ant-eating spiders (Araneae: Zodariidae) of Portugal: additions to the current knowledge. Zootaxa.

[B4996692] Pekár Stano, Cardoso Pedro, Barriga Javier C., Carvalho José C. (2011). Update to the zodariid spider fauna of the Iberian Peninsula And Madeira (Araneae: Zodariidae). Zenodo.

[B5008959] Phillips Steven, Anderson Robert, Schapire Robert (2006). Maximum entropy modeling of species geographic distributions. Ecological Modelling.

[B5008883] Republic Portuguese Natura 2000 Network - Decree-Law n 140/99. https://data.dre.pt/eli/dec-lei/140/1999/p/cons/20131108/pt/html.

[B5008863] Pryke James S., Samways Michael J. (2011). Importance of using many taxa and having adequate controls for monitoring impacts of fire for arthropod conservation. Journal of Insect Conservation.

[B4998310] Pulido L. P., del Pozo B. S. (2010). Nuevas citas de *Macrothele
calpeiana* (Walckenaer, 1805) en la provincia de Jaén (España). Revista Iberica de Aracnologia.

[B4997141] Reboleira A., Borges P., Gonçalves F., Serrano A., Oromí P. (2011). The subterranean fauna of a biodiversity hotspot region - Portugal: an overview and its conservation. International Journal of Speleology.

[B4996591] Reboleira Ana (2012). Biodiversity and conservation of subterranean fauna of Portuguese karst.

[B4996611] Ribera C. (1988). Descripción del macho de *Nesticus
lusitanicus* Fage, 1931 (Araneae, Nesticidae). Publicaciones del Departamiento de Zoología de Barcelona.

[B4997131] Ribera C. (1993). *Dysdera
caeca* n. sp. y *Harpactea
stalitoides* n. sp. (Araneae), dos nuevas especies cavernícoles de Marruecos y Portugal.. Revue Arachnologique.

[B4996601] Ribera Carles, López-Pancorbo Alberto (2011). *Nesticus
baeticus* sp. n., a new troglobitic spider species from south-west Europe (Araneae, Nesticidae). ZooKeys.

[B4996818] Sousa P. (2006). Caracterizacao da fauna de aracnideos do Parque Natural da Serra da Estrela. Inventario, distribuicao altitudinal, ecologia e cartografia. Universidade do Porto.

[B5309698] Tavares C., Gouveia A., Crespo L., Mateus C., Rebelo M. (2007). Spider (Arachnida: Araneae) on pear orchards in the "Oeste" region of Portugal.

[B4997853] Wunderlich J. (2011). Fossile und Heutige Spinnen.

[B4998234] Wunderlich J. (2017). Descriptions, notes and synonyms of some mainly Mediterranean and Macaronesian spiders (Araneae) of various families.. Beiträge zur Araneologie.

[B5309754] Zahn A., Rainho A., Rodrigues L., Palmeirim JM. (2009). Low macro-arthropod abundance in exotic *Eucalyptus* plantations in the Mediterranean. Applied Ecology and Environmental Research.

[B4998269] Zonstein S., Marusik Y., Grabolle A. (2018). A remarkably small new species of *Filistata* (Aranei: Filistatidae) from Portugal.. Arthropoda Selecta.

